# Venom Complexity in a Pitviper Produced by Facultative Parthenogenesis

**DOI:** 10.1038/s41598-018-29791-y

**Published:** 2018-08-01

**Authors:** J. J. Calvete, N. R. Casewell, U. Hernández-Guzmán, S. Quesada-Bernat, L. Sanz, D. R. Rokyta, D. Storey, L.-O. Albulescu, W. Wüster, C. F. Smith, G. W. Schuett, W. Booth

**Affiliations:** 1 Evolutionary and Translational Venomics Laboratory, CSIC, Valencia, Spain; 2 Alistair Reid Venom Research Unit, Parasitology Department, Liverpool School of Tropical Medicine, Pembroke Place, Liverpool, L3 5QA UK; 3 Laboratorio de Química de Biomacromoléculas, Instituto de Química, Universidad Nacional Autónoma de México, Ciudad Universitaria, Delegación Coyoacán C.P, 04510 Ciudad de México, Mexico; 4 Department of Biological Science, Florida State University, Tallahassee, FL USA; 5 Molecular Ecology and Fisheries Genetics Laboratory, School of Biological Sciences, Environment Centre Wales, Bangor University, Bangor, LL57 2UW UK; 6 Chiricahua Desert Museum, P.O. Box 376, Rodeo, NM USA; 7 The Copperhead Institute, P.O. Box 6755, Spartanburg, SC USA; 8 Department of Biology, Wofford College, 429 North Church Street, Spartanburg, SC USA; 9 Department of Biology and Neuroscience Institute, Georgia State University, Atlanta, GA USA; 10 Department of Biological Science, The University of Tulsa, Tulsa, OK USA

## Abstract

Facultative parthenogenesis (FP) is asexual reproduction in plant and animal species that would otherwise reproduce sexually. This process in vertebrates typically results from automictic development (likely terminal fusion) and is phylogenetically widespread. In squamate reptiles and chondrichthyan fishes, FP has been reported to occur in nature and can result in the production of reproductively viable offspring; suggesting that it is of ecological and evolutionary significance. However, terminal fusion automixis is believed to result in near genome-wide reductions in heterozygosity; thus, FP seems likely to affect key phenotypic characters, yet this remains almost completely unstudied. Snake venom is a complex phenotypic character primarily used to subjugate prey and is thus tightly linked to individual fitness. Surprisingly, the composition and function of venom produced by a parthenogenetic pitviper exhibits a high degree of similarity to that of its mother and conspecifics from the same population. Therefore, the apparent loss of allelic diversity caused by FP appears unlikely to have a significant impact on the prey-capturing ability of this snake. Accordingly, the pitviper offspring produced by FP retained complex phenotypic characteristics associated with fitness. This result reinforces the potential ecological and evolutionary importance of FP and questions our understanding of the inheritance of venom-associated genes.

## Introduction

**Figure 1:**
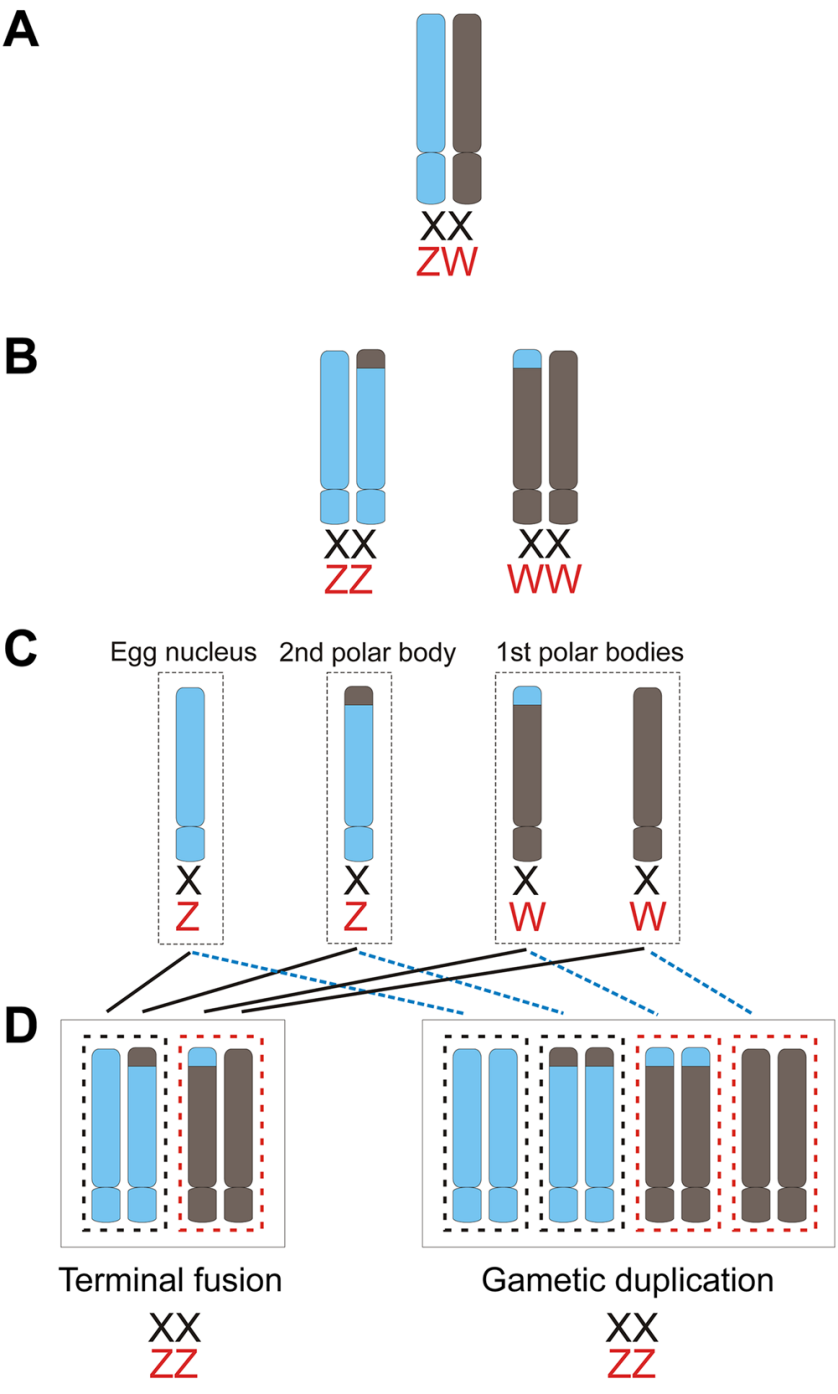
Mechanisms of automixis in snakes. Black = *Boa* and *Python* spp., Red = caenophidian snakes (including *A. contortrix*). (**A**) Primordial germ cell. (**B**) Meiotic products following DNA replication and recombination during first cell division. (**C**) Meiotic products following second cell division. (**D**) Potential chromosomal arrangements following terminal fusion (black lines) and gametic duplication (blue dotted lines). Note that WW arrangements (depicted in red dashed boxes) are considered non-viable^4^.

Facultative parthenogenesis (FP) has been reported across the animal kingdom, including all major lineages of jawed vertebrates except mammals^1–4^. In these lineages, it has been shown that females have the potential to switch from sexual to asexual (FP) reproduction^5,6^ and produce consecutive parthenogenetic births^5–11^. Furthermore, second generation FP has been observed in both sharks^12^ and snakes (Booth W, unpublished data). Progeny resulting from FP suffer from near genome-wide reductions in heterozygosity due to automictic development^13^ (Fig. 1) (but see^14^ and response^15^), and are essentially half-clones of their mother due to the fusion of the second polar body with the egg nucleus. Because FP in vertebrates was originally only observed in captive situations, it was relegated by some authorities to be an outcome of “reproductive error” and consequently of limited evolutionary significance. However, the plethora of recent reports in birds, lizards, snakes, and sharks, its discovery in natural populations, and recent evidence of the reproductive viability of parthenogens, has altered this initial perspective^4,6,13,16–19^; highlighting the need for focused research into the ecological and evolutionary significance of FP in vertebrates.

In this study we assess the implications that the dramatic loss of genetic diversity resulting from automictic development might have on free-living parthenogens. We accomplish this by measuring the compositional and functional phenotypes of venom from the copperhead snake, *Agkistrodon contortrix*, a common and widespread North American pitviper species. Facultative parthenogenesis has been reported from a number of different pitviper species, suggesting that this mode of reproduction may occur frequently in this group of snakes^4,17,20^. To date, however, the only studies that report the phenotypic consequences relating to FP in vertebrates have focused on colour and patterns, with parthenogenetic offspring exhibiting homozygosity, regardless of whether that specific trait is inherited recessively or through incomplete dominance^5,15^.

In contrast to those previous examples, snake venoms are complex mixtures of protein and peptide components (circa 20–200 per species), commonly referred to as toxins, and this phenotype is therefore underpinned by dozens of gene loci^21^. Moreover, venom is variable, with toxin constituents reported to vary at every taxonomic level, including intra-specifically between geographically distinct populations or intra-individually during ontogenetic development^22–24^. Crucially, while venom may be deployed defensively, it is primarily used to subjugate prey and thus it is clearly and tightly linked to individual fitness. Although past studies have suggested that venom components are heritable (e.g.^25–29^), no direct comparisons have been made among related *vs*. unrelated individuals. If venom traits of snakes show Mendelian inheritance, the progeny resulting from FP should theoretically possess venoms that lack a paternal contribution and therefore exhibit reduced heterozygosity and phenotypic complexity. If borne out, such FP progeny might be at a disadvantage in regards to venom function and incur loss of fitness.

## Results

**Figure 2:**
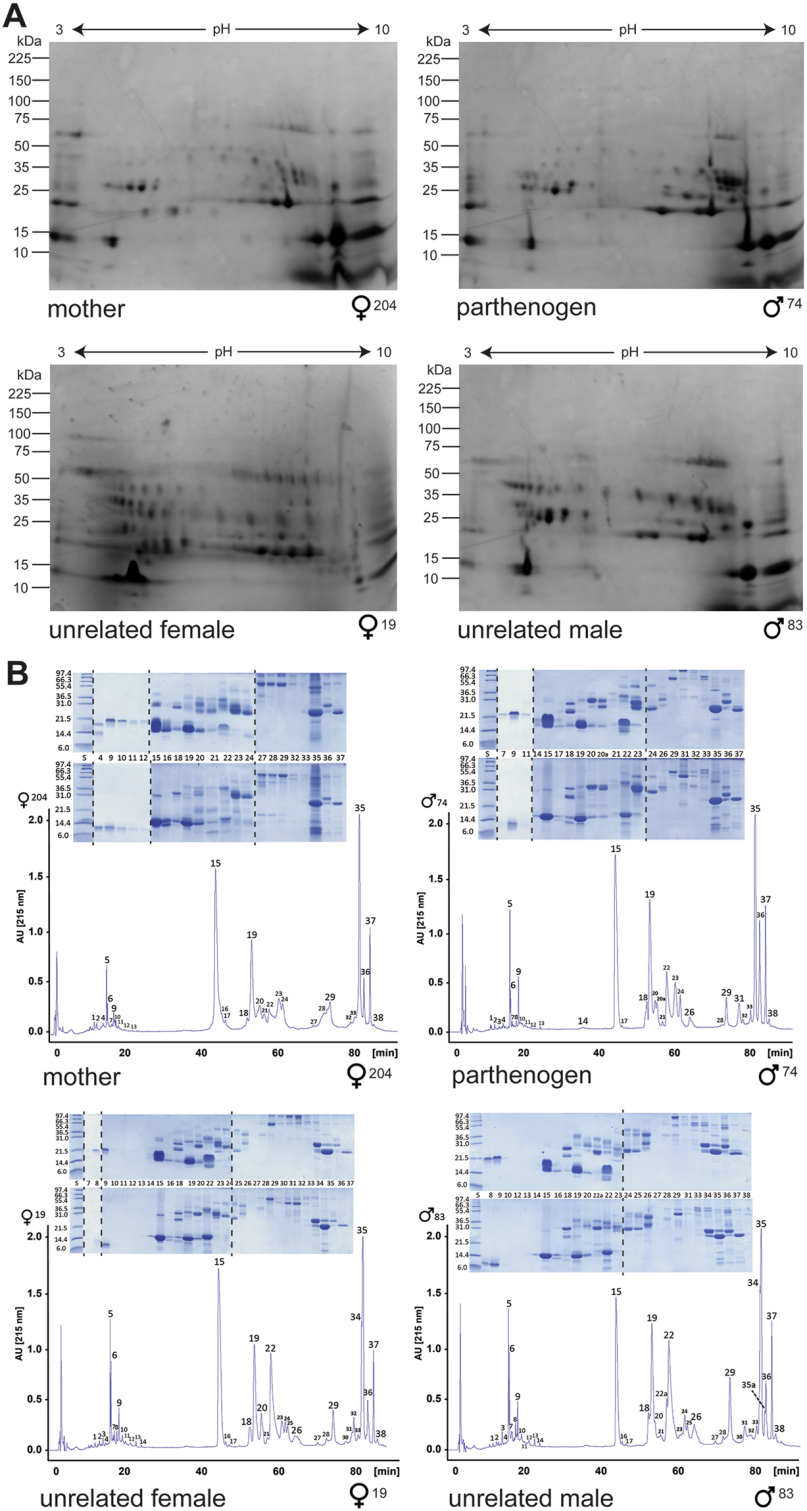
Proteomic 2D gel electrophoretic and RP-HPLC chromatographic profiles of the parthenogen, mother and unrelated snakes. (**A**) 2D SDS-PAGE gel electrophoresis profiles of venom from the wild-caught parthenogen mother (204F), her parthenogen male offspring (74M), and unrelated female (19F) and male (83M) individuals of the same age as the parthenogen from the same population. (**B**) RP-HPLC separations of venom from the same individuals. Chromatographic peaks were collected manually and analysed by SDS-PAGE (insets) under non-reduced (upper panels) and reduced (lower panels) conditions. Dashed lines in insets indicated where different SDS-PAGE gels were used to visualise the various fractions. Protein bands were excised and characterized by LC-nESI-MS/MS (Tables S1–S4).

To assess the consequences of FP on this crucial phenotype, we collected venom from an adult male copperhead snake produced through FP (terminal fusion automixis) from a wild-collected pregnant female (see^17^), and compared its venom composition and function with that of its mother and two conspecifics (one male and one female, unrelated to each other) of the same age as the parthenogen and from the same population^30^. We first used two-dimensional gel electrophoresis (2D-E) to provide a broad comparative overview of the venom proteome profiles of these four snakes. Our results demonstrate that the parthenogen exhibits considerable complexity in venom composition, with a wide diversity of venom proteins being detected at varying molecular weights and isoelectric points (Fig. 2A). Most importantly, the venom profile is comparable with that of its mother, with similar levels of venom toxin complexity. Additionally, these venom profiles are similar to those from the two unrelated individuals, although these venom profiles appear to have a number of additional proteins absent from both the parthenogen and its mother, and also show minor variation in toxin composition when compared with each other (Fig. 2A).

To provide a higher resolution analysis, venom proteins found in each of the four venoms were separated using a combination of reverse-phase high-performance liquid chromatography (RP-HPLC) and one-dimensional SDS-PAGE gel electrophoresis of the isolated chromatographic fractions. We then identified the resulting venom constituents by mass spectrometric ‘venomic’ analyses^31^, underpinned by a species-specific venom gland transcriptome database (Tables S1–S4). Corresponding with our 2D-E analysis, the four venoms exhibited conserved chromatographic and electrophoretic profiles (Fig. 2B) that were also similar to previously reported *Agkistrodon* venoms, including *A. contortrix*^32^.

**Figure 3:**
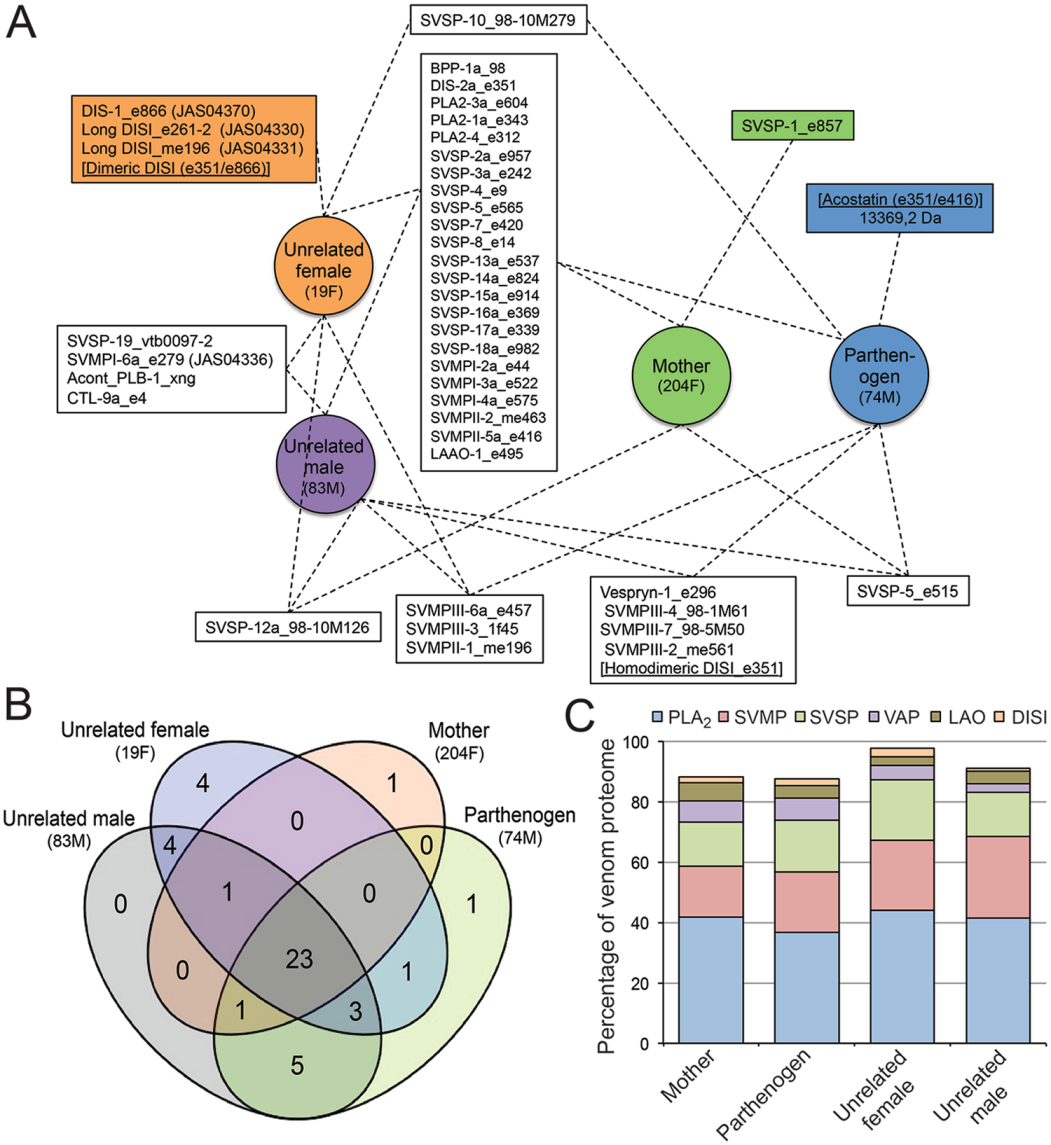
Distribution of toxin genes between the venoms of the parthenogen, mother and unrelated snakes. (**A**) Boxes linked by discontinuous lines to multiple individuals show proteins shared between their venoms, with the central box showing those shared across all four individuals. Proteins only found in the venom of a single individual are highlighted by coloured boxes. (**B**) Venn diagram highlighting the number of unique and shared venom proteins between the mother (204F), parthenogen (74M) and the unrelated female (19F) and male (83M) individuals of the same age as the parthenogen. (**C**) Toxin proteins shared across all four of the sampled *A. contortrix* individuals account for the vast majority of the protein abundance in each venom. Bar charts show the summed abundance of those toxins found in all four individuals (central box in A) as a percentage of the total toxin abundance in each species. Each bar is broken down by toxin family: PLA_2_ – phospholipase A_2_; SVMP – snake venom metalloproteinase; SVSP – snake venom serine protease; VAP – vasoactive peptides; LAO – L-amino acid oxidase; DISI - disintegrin.

**Figure 4:**
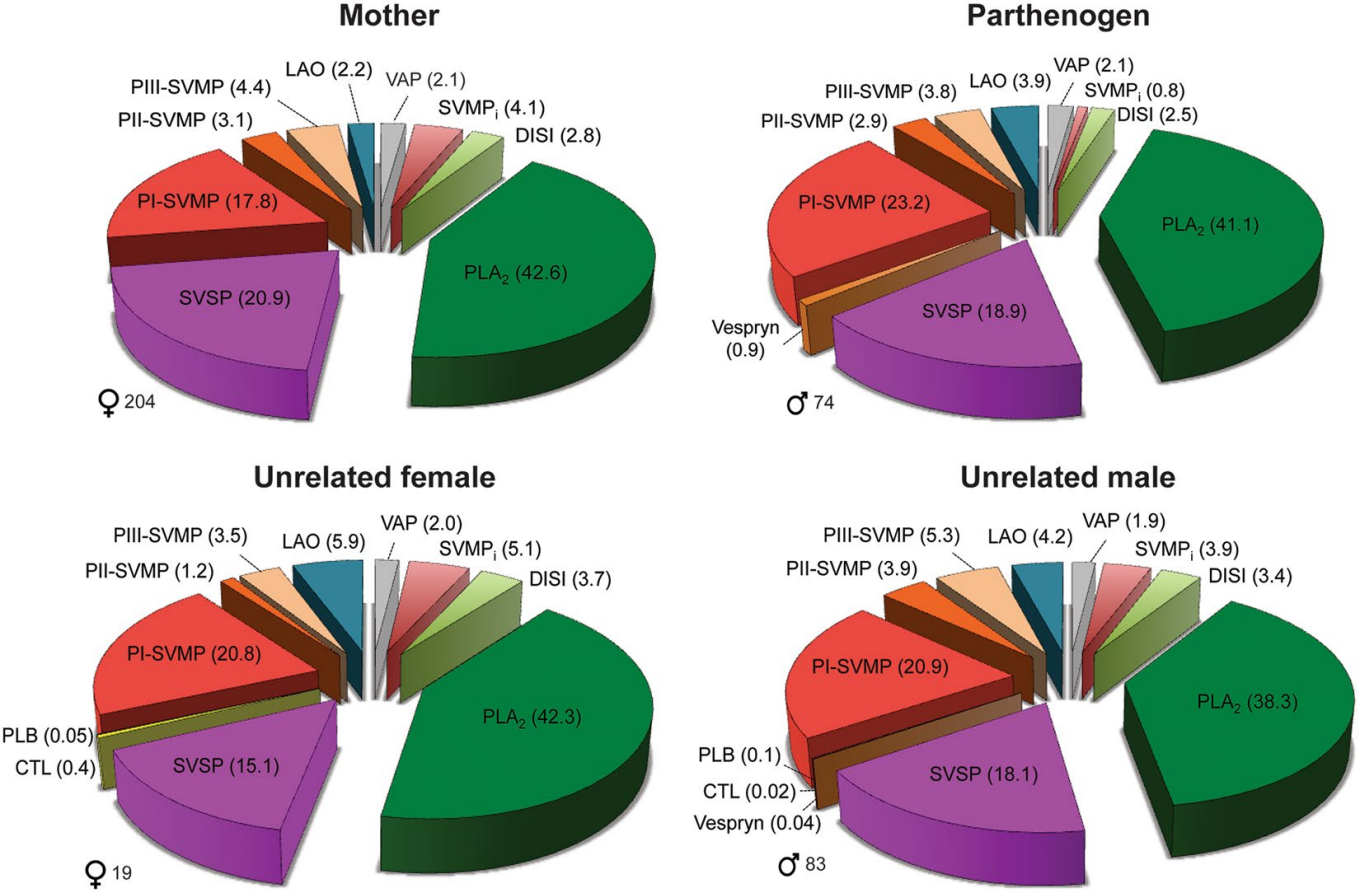
The proteomic representation of toxin families found in the venom of the mother, parthenogen and unrelated snakes. Numbers in parentheses represent percentages of toxin proteins identified in venom by reverse-phase HPLC, SDS-PAGE and mass spectrometry experiments. Acronyms represent the following snake venom toxin families: PLA_2_ – phospholipases A_2_; SVSP – snake venom serine proteases; CTL – C-type lectins; PLB – phospholipase B; P-I, P-II and P-III SVMP – P-I, P-II and P-III classes of snake venom metalloproteinases; LAO – L-amino acid oxidase; VAP – vasoactive peptides; SVMPi – snake venom metalloproteinase inhibitors; DISI – disintegrins.

Despite apparent conservation in terms of venom profiles, a detailed venomic analysis revealed a complex pattern of similarities and differences among the four venoms (Figs 3 and 4 and Table S5). The majority (23) of the 42 toxin gene products unambiguously assigned by mass spectrometry were found across all four venoms, demonstrating a degree of conservation in venom composition across the sampled individuals. Importantly, these 23 toxin gene products account for between 87.5% and 97.7% of the total toxin abundance detected in each of the venoms, demonstrating that the degree of intra-specific venom variation that exists is broadly restricted to toxins of low abundance (Fig. 3). All venoms contained typical pitviper venom toxin types, such as vasoactive peptides (VAP [bradykinin potentiating and inhibiting peptides]), disintegrins, phospholipases A_2_ (PLA_2_), snake venom serine proteases (SVSP), snake venom metalloproteinases (SVMP), and L-amino acid oxidases (LAO). Furthermore, in the case of PLA_2_, SVSP and SVMPs, multiple isoforms of those toxin types were found in all four venoms (Fig. 4). These three toxin families are the most abundant in terms of their proteomic representation – PLA_2_s account for 38.3–42.6% of the total toxin abundance across all four venoms, SVMPs 25.3–30.1% and SVSPs 15.1–20.9% (Fig. 4). In contrast, C-type lectin (CTL), vespryn, and phospholipase-B toxin types were only found in the venom of certain individuals, although their proteomic representation was low in each case (<1.0%) (Fig. 4).

Remarkably, the parthenogen contained more distinct venom toxins than its mother (34 and 26 respectively), and comparable numbers to the two unrelated congeners (36 and 37 toxins), demonstrating that reproduction by FP did not result in a detectable loss of venom toxin-encoding gene diversity or a reduction in the venom compositional phenotype. However, we did detect two venom toxins from the mother that were not found in the venom of the parthenogen (SVSP-1 and SVSP-12a) (Fig. 3), although each of these constituents was found to be a very minor component of the mother’s venom proteome (representing 0.8% and 0.2% of all toxins respectively).

Conversely, we found ten toxins present in the venom of the parthenogen that were not detected in that of its mother (Fig. 3). On the surface this is, perhaps, a surprising result, given that FP is expected to result in near-genome-wide homozygosity, and we would not anticipate major distinctions in venom composition of the offspring to that of its mother. However, it is possible that these differences may be underpinned by the non-expression of recessive loci in the heterozygous mother, which are then inherited in a homozygous manner in the parthenogenetic offspring. Alternatively, previous work has demonstrated that changes in venom composition can occur over the lifetime of snakes, with different toxins being differentially regulated at different life stages^22,24,33–35^. It is therefore possible that, despite being a young adult (~3 years of age upon sampling), the presence of these toxins might be associated with the parthenogen’s age in comparison to that of its mother (unknown age but at least 6 years old). This is supported by evidence that nine of these 10 toxins absent from the mother’s venom were detected in at least one of the two venoms sampled from the two unrelated snakes, both of which are age-matched to the parthenogen and sampled from the same population (Fig. 3). Irrespective of the mechanism, in combination, these toxins contribute less than 5% of the total toxin abundance found in the venom of the parthenogen, reinforcing the fact that the vast majority of abundant venom toxins are shared between mother and FP offspring (Fig. 3). Acostatin^36,37^ (a heterodimeric disintegrin with an isotope-averaged molecular mass of 13,369.7 Da) was the only venom constituent found to be unique to the parthenogen copperhead (Fig. 3), although its proteomic abundance was very low (0.11% of all toxins).

In total, these data show that the venom of the parthenogen and mother are remarkably similar (Fig. 3), and that variations between them are only found in toxins that are minor constituents of the venom proteome, as previously proposed^38^ (but see also^39^). However, the venom of the parthenogen is slightly less complex compositionally than that of unrelated individuals of the same age produced by sexual reproduction. This may perhaps be the result of two overlapping effects: (i) the non-equivalent contribution of gene toxin-encoding alleles from both progenitors, and (ii) individual intra-population variability. The latter could be potentially important if the minor venom components we observed here are not under strong selection and vary as the result of stochastic fluctuations within populations, although even minor components have been shown to play a role in local adaptation of venom in other snakes^40^. Alternatively, or in addition, postgenomic mechanisms^24,41,42^ may differentially affect the relative expression of gene loci. Such transcriptional/translational/post-translational regulation seems likely to influence the ontogenetic variations observed in the venoms of some snakes^22,24,34,35^ and may also underpin the minor variations in toxin composition found here between the mother, her parthenogen offspring, and the two conspecifics.

Having determined that FP did not substantially reduce the complexity of venom composition, we next sought to test whether the functional phenotype of the parthenogen’s venom remained comparable to that of its mother. To do so, we used a range of *in vitro* functional assays that measured the following relevant venom bioactivities: (i) thrombin-like enzyme activity by chromogenic assay, (ii) fibrinogenolytic activity by degradation SDS-PAGE gel electrophoresis, (iii) procoagulant venom function by spectrophotometric assessments of plasma coagulation, and (iv) enzymatic PLA_2_ activity by fluorescent assay.

**Figure 5:**
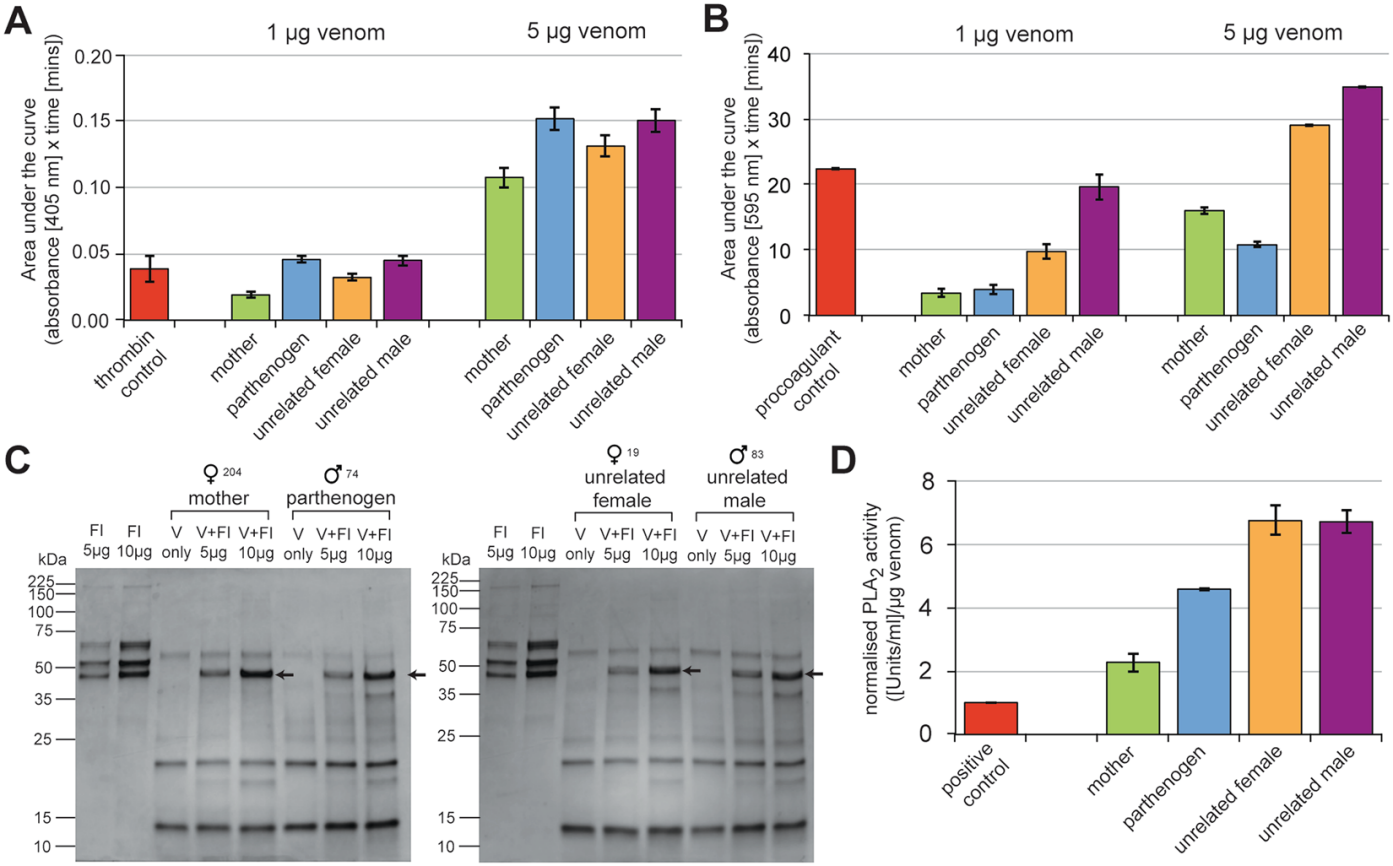
Functional comparisons of bioactivity between the venoms of the parthenogen, mother and unrelated snakes. (**A**) The thrombin-like enzyme activity of *A. contortrix* venoms measured by chromogenic assay at two venom doses. (**B**) Coagulopathic venom activity measured by a plasma coagulation assay at two venom doses. For both (**A** and **B**) bars represent mean areas under the curve (absorbance *vs*. time) of triplicate measurements and error bars represent SEM. (**C**) The fibrinogenolytic activity of *A. contortrix* venoms by degradation SDS-PAGE gel electrophoresis. The fibrinogen (FI) control lanes show three distinct bands representing the α, β and γ chains of fibrinogen, respectively. Arrows in venom (V) and fibrinogen lanes (V + FI) highlight the retention of only one of these three bands (the γ chain) following incubation, demonstrating the degradation of α and β chains. (**D**) The enzymatic phospholipase A_2_ (PLA_2_) activity of the venoms measured by fluorescent enzyme assay. Bars represent PLA_2_ activity (Units/ml) per μg of venom normalised to a positive control (venom from the rattlesnake *Crotalus atrox*), and error bars represent SEM of three independent triplicate measurements.

Pitviper venoms typically contain SVSPs, and many of these toxins act as thrombin-like enzymes by cleaving fibrinogen into fibrin, which in turn is cross-linked to form clots^43,44^. We assessed the thrombin-like enzyme activity of each venom sample by measuring the cleavage of a thrombin-specific substrate (S-2238) in a chromogenic assay. All four venoms exhibited detectable thrombin-like enzyme activities (Figs 5A and S1). The parthenogen was found to exhibit the highest activity at both of the venom doses tested (1 μg and 5 μg), although these results were similar to those obtained from the venom of the unrelated male snake, and to a lesser extent, the unrelated female individual. Despite having the highest proportion of SVSPs in its proteome (20.9% *vs* 15.1–18.9%; Fig. 4), venom from the parthenogen’s mother had the lowest thrombin-like enzyme activity, accounting for only 42% and 71% of the parthenogen’s activity at the low and high venom doses, respectively (Fig. 5A). This apparent disconnect between gross proteomic abundance and functional activity is almost certainly the result of some, but not all, of the venom SVSPs acting in a thrombin-like manner^45^.

We found that all four of the venoms exhibited comparable fibrinogenolytic activities. Following the incubation of each venom with fibrinogen, our SDS-PAGE gel electrophoresis experiments revealed the preferential and complete degradation of both α and β chains of fibrinogen, while the γ chain remained intact (Fig. 5C). This enzymatic cleavage of fibrinogen chains is caused by members of the SVSP and/or SVMP toxin families^43–45^, both of which are comparable in terms of their proteomic abundance across the four individuals sampled here (Fig. 4).

Thrombin-like enzyme and fibrinogenolytic venom activities act together to perturb physiological fibrinogen, resulting in haemostatic disturbances^43–45^. But snake venom toxins are known to act upon many other targets related to the blood clotting cascade (e.g. prothrombin, Factor X, plasmin, platelets) to disrupt haemostasis^43–45^. The toxin types involved are also diverse and include members of the SVMP, SVSP, PLA_2_, CTL and disintegrin toxin families^44^, all of which were identified in each of the *A. contortrix* venoms analysed here, with the exception of the CTLs which were only detected in the venom of the unrelated female and male individuals (Fig. 4). Therefore, to assess the combined coagulopathic function of each of the four copperhead venoms, we used a small-scale spectrophotometric plasma clotting assay^46^. Our results revealed substantial differences in the coagulopathic effect of the four venoms (Fig. 5B). At both doses tested (1 μg and 5 μg), venoms from the unrelated male and female individuals were substantially more coagulopathic than that of both the mother and parthenogen, with venom from the unrelated male snake being the most potent overall. While we detected no difference between the plasma clotting ability of venom from the mother and parthenogen at the low venom dose, at the high dose the mother exhibited an increased venom activity compared to her offspring (Figs 5B and S1). However, the synergistic contribution of numerous toxins potentially involved in this venom activity makes determining which compositional differences are responsible for this functional variation challenging.

Since PLA_2_s are the most abundant toxin family detected in the venom of each of the four sampled copperheads (38.3–42.6% of all toxins) (Fig. 4), we next sought to assess and compare venom PLA_2_ activity. To do so, we used a continuous secretory PLA_2_ fluorescent substrate assay, which detects enzymatic PLA_2_ activity. Similar to the plasma assay, the PLA_2_ assay showed that the venoms of the unrelated female and male individuals exhibit substantially higher venom activity than both the mother and parthenogen (Fig. 5D). We find in this case, however, that venom from the parthenogen has higher enzymatic PLA_2_ activity than that of its mother. The patterns of PLA_2_ venom activity only show limited association with the proportion of PLA_2_ toxins found in each of the venom proteomes (Fig. S1), but when analysing the proportions of enzymatic venom PLA_2_ toxins only (those with an aspartic acid residue at position 49^47,48^), we find a positive relationship between venom toxin composition and PLA_2_ activity (Fig. S1).

In summary, our functional assessments demonstrate that venom from the parthenogen exhibits equipotency to that of its mother in terms of fibrinogenolytic activity and coagulopathic activity (at the low venom dose), and increased potency in terms of thrombin-like enzyme activity and enzymatic PLA_2_ activity (Fig. 5). However, at higher doses the venom of the parthenogen was found to be less coagulopathic than that of its mother. Broadly speaking though, we do not observe consistent, marked, functional differences between the parthenogen and its mother, strongly suggesting that FP has not adversely affected the functionally complex phenotype of the parthenogen’s venom.

## Discussion

The discovery of a functionally active and compositionally equivalent pitviper venom from a facultative parthenogen compared to its mother (and two unrelated conspecifics sampled from the same genetically diverse population^30^), contradicts our initial expectations that assumed reduced complexity due to a complete or near-complete loss of allelic diversity resulting from automictic parthenogenesis^4^. Automixis restores diploidy either through the fusion or duplication of meiotic products. Terminal fusion, resulting from fusion of an egg nucleus with a second polar body, is assumed to retain some level of heterozygosity due to recombination distal to the centromere and proximal to the chromosome tips, whereas gametic duplication is predicted to result in the complete loss of heterozygosity, barring spontaneous mutations, due to the absence of recombination (Fig. 1). The accurate assignment of which mechanism underlies FP in a given species has been challenging owing to the molecular markers previously employed, primarily microsatellite DNA^4^. However, current research employing double-digest restriction site associated DNA sequencing (RADseq) support the former mechanism of terminal fusion over gametic duplication^49^ (Booth W, unpublished data).

Assuming that all species of snake capable of FP employ the same automictic mode^4^, these findings of near genome-wide homozygosity suggest that only limited allelic diversity should have been retained in the copperhead parthenogen examined here. Under a simple recessive or incomplete dominant framework, it may be predicted that a dropout of venom proteins might be observed in a parthenogen relative to the heterozygous mother, which could result in a reduction or loss of complexity and function in the parthenogen. However, if venom-genes are encoded by multiple paralogous loci, as recently shown for rattlesnakes^50^, the effect of automixis-driven homozygosity might be reduced due to identical gene loci compensating for such losses, and thus resulting in a buffering effect. Furthermore, if copies of venom encoding genes are located in chromosomal regions where elements of heterozygosity might be retained, this may further mitigate the impact of FP on venom complexity and/or function. Ultimately, the high level of similarity observed here between the venoms of the mother and her parthenogenetic offspring suggests that allelic variation is perhaps not a major contributor to venom composition. If venom toxin-encoding genes are predominately homozygous to begin with, the process of FP would not be anticipated to dramatically affect the resulting venom phenotype.

An unknown combination of the processes described above seem likely to explain the remarkable degree of toxin similarity (with minor elements of variation) observed here between the venoms of the parthenogen and its mother. These findings highlight the glaring gap in our knowledge regarding the inheritance of venom-associated genes and the relative importance of allelic and locus diversity in generating venom composition. In a similar manner to the use of FP to stimulate research that identified the dichotomous sex chromosome systems used by snakes^51^, examples of FP seem likely to prove informative as models to better understand the inheritance of venom genes.

Despite the extensive loss of allelic diversity expected under automixis, we conclude that reproduction by FP has not resulted in major losses of venom toxin complexity or reduced functional activities. It therefore seems unlikely that FP would have a significant impact on the venom phenotype and prey-capturing ability of parthenogenetic pitviper snakes. Consequently, these results reinforce the viability of FP as a potentially important mode of reproduction in vertebrate lineages and should stimulate further research on the wider phenotypic consequences and evolutionary significance of FP in natural animal populations.

## Methods

### Samples

#### Study site

The source of the current subjects was a study site located in a 485 ha parcel of basalt trap rock ridge ecosystem situated 4.75 km NW of Meriden, Connecticut. Details of topography and climate of this region are presented in Smith *et al*.^52^. All experimental protocols relating to our research on copperheads were conducted in accordance with relevant guidelines and regulations under approvals granted from The University of Connecticut and Wofford College Institutional Animal Care and Use Committee (IACUC), protocol numbers S211-1201 and 802, respectively.

#### Study subjects

Adult female copperheads suspected of being pregnant were captured in late July or August 2011. All were checked for prior identification (passive integrated transponders, PIT-tags; 125 kHz 12 mm, Biomark, Boise, Idaho, USA; see^52^). PIT-tags were injected one-third of the body length anterior from the cloaca and the last three characters of the 10-character PIT code were used as an identification code for all records pertaining to an individual. Three females (F118, F204, and F368) were transported to the laboratory (Wofford College, Copperhead Institute, Spartanburg, South Carolina, USA) for general processing and maintained until parturition. All three females gave birth in 2011, and the progeny were maintained in captivity. Four subjects were used for the present venom analyses: one male (M83 from F118) and one female (F19 from F368) progeny, and the adult F204, and her parthenogen son (M74). Male 74 has been discussed in a previous paper^17^. All venom was collected in summer 2014. The age of the laboratory-born subjects was 3 years. The mother (F204) that bore the male parthenogen (M74) appeared young but estimated at ≥5 years-old, as sexual maturity in this species is reached within 2–3 years^53^. All subjects are alive at the Copperhead Institute at the time of writing.

#### Venom samples

Using approved procedures for handling venomous snakes, the four subjects were safely restrained in an appropriate-size clear, acrylic tube. Venom from the four snakes used in these analyses was harvested by allowing each subject’s head to exit the tube and they were encouraged to bite through Parafilm^®^ (Bemis, Inc., Neenah, Wisconsin, USA) that was tightly stretched over a glass funnel fitted with a 1.5 ml sterile plastic centrifuge tube; the expelled venom gravity-flowed directly into the centrifuge tube. After collection, the venom was immediately processed (lyophilized) and stored in a new sterile 1.5 microcentrifuge tube sealed with Parafilm^®^, before long term storage at 4 °C prior to experimental use.

### Venom gland transcriptomics

The venom gland transcriptome for *A. contortrix* was sequenced on an Illumina HiSeq with 100-nucleotide paired-end reads using the method described previously^54^. We generated 103,979,548 read pairs, which were submitted to the National Center for Biotechnology Information (NCBI) Sequence Read Archive (SRA) under the accession number SRR2032114. The initial assembly (TSA accession GDAY01000000) was generated as described^53^ and subsequently used by Rokyta *et al*.^55^. To further verify and improve the original assembly, we merged the original paired-end reads using PEAR version 0.9.5^56^. We assembled 10 million merged reads with SeqMan NGen version 12.2.0, five million merged reads using VTBuilder^57^, and all merged and unmerged reads with Trinity version 2.0.6^58^. We also built transcripts using our in-house Extender assembler^59^, starting from 1,000 merged reads as seeds. Full-length toxins were identified by means of blastx searches against the National Center for Biotechnology Information (NCBI) nonredundant protein database (nr). Any new toxin sequences were added to the previous assembly. We screened for chimeric sequences by checking for multi-modal coverage distributions after aligning the merged reads against the final set of transcripts using bowtie2 version 2.2.5^60^. The final transcriptome used in this study consisted of 66 toxin and 2,954 non-toxin full-length protein-coding sequences. The NCBI Transcriptome Shotgun Assembly (TSA) accession for the new version of this assembled transcriptome is GDAY00000000.2.

### Two dimensional gel electrophoresis

We performed two dimensional (2D) SDS-PAGE gel electrophoresis experiments using each of the four *A. contortrix* venoms to compare venom compositional profiles. For each gel, 0.5 mg of venom was prepared for 2D gel electrophoresis using the ReadyPrep™ 2-D Cleanup Kit for isoelectric focusing (IEF) (Bio-Rad) as per the manufacturer’s instructions. Cleaned-up venom samples were then applied to 7 cm, pH 3–10, non-linear IPG strips (Bio-Rad) using the ReadyPrep™ 2-D starter kit (BioRad), as per manufacturer’s instructions, and re-hydrated overnight at room temperature. After re-hydration, IEF was performed using a PROTEAN^®^ IEF Cell (Bio-Rad) with the manufacturer’s standard electrophoresis protocol for 7 cm IPG strips (default cell temperature = 20 °C; maximum current 50 Ua/strip; voltage = 250 V with linear ramp for 20 min; 4000 V with linear ramp for 2 hours; 4000 V with rapid ramp for 10,000 V-hr). After IEF, IPG strips were equilibrated (as per the ReadyPrep™ 2-D starter kit) and loaded onto Mini-PROTEAN TGX AnyKd precast gels (Bio-Rad) and run at 200 V for 35 minutes. Gels were then rinsed in water and stained with G-250 coomassie blue stain (Bio-Rad) for 1 hr to visualise proteins. Original (unedited) gel images can be found in Fig. S2.

### Reverse-phase HPLC, SDS-PAGE and mass spectrometry

For each individual, we dissolved 0.5 mg of crude, lyophilized venom in 200 μL of 5% acetonitrile in MilliQ^®^ (Millipore Co.) water containing 0.1% trifluoroacetic acid (TFA), centrifuged the sample to remove debris, and then separated by reverse-phase (RP) HPLC using a Teknokroma Europa Protein 300 C18 (0.4 × 25 cm, 5 μm particle size, 300 Å pore size) column and an LC 1100 High Pressure Gradient System (Agilent Technologies, Santa Clara, CA, USA) equipped with DAD detector and micro-Auto-sampler. The flow rate was set to 1 mL/min and the column was developed with a linear gradient of 0.1% TFA in water (solution A) and acetonitrile (solution B) using the following column elution conditions: isocratically (5% B) for 5 min, followed by 5–25% B for 10 min, 25–45% B for 60 min, and 45–70% B for 10 min. Protein detection was carried out at 215 nm with a reference wavelength of 400 nm. Fractions were collected manually, dried in a vacuum centrifuge (Savant), redissolved in water, and submitted to SDS-PAGE analysis in 15% polyacrylamide gels, under reducing and non-reducing conditions. Gels were stained with Coomassie Brilliant Blue R-250 (Sigma-Aldrich, St. Louis, MO, USA). Electrophoretic protein bands were excised from Coomassie Brilliant Blue-stained SDS-PAGE gels and subjected to in-gel reduction (10 mM dithiothreitol) and alkylation (50 mM iodoacetamide), followed by overnight sequencing-grade trypsin digestion (66 ng/μL in 25 mM ammonium bicarbonate, 10% acetonitrile; 0.25 μg/sample) in an automated processor (ProGest Protein Digestion Workstation, Genomic Solution Ltd., Cambridgeshire, UK) following the manufacturer’s instructions. Tryptic digests were dried in a SpeedVac (Savant™, ThermoScientific Inc., West Palm Beach, FL, USA), redissolved in 15 μL of 0.1% formic acid in water, and submitted to LC-MS/MS (Eichberg *et al*. 2015). To this end, tryptic peptides were separated by nano-Acquity UltraPerformance LC^®^ (UPLC^®^, Waters Corporation, Milford, MA, USA) using BEH130 C18 (100 μm × 100 mm, 1.7 μm particle size) column in-line with a SYNAPT^®^ G2 High Definition Mass Spectrometry System (Waters). The flow rate was set to 0.6 μL/min, and the column was developed with a linear gradient of 0.1% formic acid in water (solution A) and 0.1% formic acid in acetonitrile (solution B), isocratically - 1% B for 1 min, followed by 1–12% B for 1 min, 12%–40% B for 15 min, 40–85% B for 2 min. Doubly- and triply-charged ions were selected for collision-induced dissociation (CID) MS/MS. Fragmentation spectra were processed in Waters Corporation’s ProteinLynx Global (PLG) SERVER 2013 version 2.5.2. (with Expression version 2.0), and the generated .pkl peak list files searched against a species-specific transcriptomic dataset. MS/MS mass tolerance was set to ±0.6 Da. Carbamidomethyl cysteine and oxidation of methionine were selected as fixed and variable modifications, respectively. MS/MS assignations were manually verified.

### *In vitro* assessments of venom function

#### Thrombin-like enzyme chromogenic assay

We applied a chromogenic assay, using the thrombin-specific chromogenic substrate S-2238 (Cambridge Biosciences), to measure the thrombin-like enzyme activity of the four *A. contortrix* venoms. We plated the reactions for each venom in triplicate onto 384-well plates and kinetically measured changes in absorbance at 405 nm for 30 minutes using a FLUOstar Omega microplate reader (BMG Labtech GmbH, Ortenberg, Germany). We initially added 15 μl of diluted venom (1 μl of venom (1 μg) and 14 μl PBS) to the plate, followed by a 3 min incubation at 37 °C. We then added 15 μl Tris buffer (100 mM Tris, 100 mM NaCl, pH 8.5) and incubated for another 3 min at 37 °C. Lastly, we added 15 μl of 6 mM S-2238 chromogenic substrate to each well and set the reaction to run at 37 °C in the plate reader. A negative control, consisting of no venom (15 μl PBS, 15 μl Tris buffer and 15 μl substrate) was used in every experiment, and a positive control, consisting of 1 μl of 0.1 Units/μl of thrombin (Sigma-Aldrich) instead of venom, was used to validate the assay. Mean measures of absorbance were plotted against time to compare venom activity with baseline (negative controls) and thrombin (positive control) readings (Fig. S1). We then subtracted the mean of the negative control readings from each of the venom and positive control readings and calculated the rate of substrate consumption for each sample by measuring the slope after 5 minutes, as follows:

$${{\rm{Rate}}}_{(\mathrm{Abs}/\min )}=({{\rm{A}}}_{405(5\min )}-{{\rm{A}}}_{405(0\min )})/5$$

where absorbance at 405 nm represent the adjusted absorbance from which the negative control has been subtracted. We then plotted the averages and the standard error of the mean for each venom (n = 3 independent repeats). We did not perform statistical comparisons of venom activity detected in this assay (or the coagulation and PLA_2_ assays described below) due to the small sample sizes used in this study.

#### Fibrinogen degradation gel electrophoresis

We used degradation SDS-PAGE gel electrophoresis^61^ to determine whether fibrinogen was cleaved (i.e. indicative of fibrinogenolytic activity) by each of the four *A. contortrix* venoms. For each venom, we performed the following experiment with lanes containing: 5 μg of fibrinogen; 10 μg of fibrinogen; 5 μg of venom; 5 μg of fibrinogen and 5 μg of venom; and 10 μg of fibrinogen and 5 μg venom. All samples were prepared and incubated for 60 minutes at 37 °C before the addition of a reduced protein loading buffer at a ratio of 1:1. Samples were then loaded onto 10-well Mini-PROTEAN TGX precast AnykD gels (Bio-Rad) alongside a protein marker (Broad Range Molecular Marker, Promega) and run at 100 V for 90 minutes using a Mini-PROTEAN Tetra System (Bio-Rad). Resulting gels were stained with coomassie brilliant blue overnight and then destained (4.5:1:4.5 methanol:acetic acid:H_2_O) for visualisation. Original (unedited) gel images can be found in Fig. S2.

#### Plasma coagulation assay

We used a small-scale plasma clotting assay^46^ to quantitate the procoagulant activity of each venom. Frozen bovine plasma (Sigma) was defrosted in warm water, placed on ice, centrifuged at 2,000 × g for 4 mins to remove precipitous proteins, and the supernatant retained for use. Five microlitre volumes of each venom sample (from the four *A. contortrix* venoms and the procoagulant positive control venom from *Echis ocellatus*) were added in triplicate to a 384 microtiter plate (transparent, flat bottom, Greiner), with each dose (1 μg and 5 μg) reconstituted in PBS. The negative control consisted of 5 μl of PBS only. Subsequently, 20 μl of 20 mM CaCl_2_ was overlaid on to each well using a ThermoScientific Multidrop Labsystems 384 Reagent Dispenser, followed by 20 μl of the plasma supernatant. The microtiter plate was read immediately on a FLUOStar Omega Spectrophotometer (BMG Labtech GmbH, Ortenberg, Germany) with the following settings: wavelength 595 nM, temperature 25 °C, well interval 10 ms, number of readings 55, kinetic interval 15 sec. As per the thrombin assay described above, mean measures of absorbance were plotted against time to compare venom activity with baseline (negative controls) (Fig. S1) and the mean of the negative control readings subtracted from each venom reading and the triplicate measurements re-plotted. We calculated areas under the curve and the standard error of the mean (of total peak areas) using default parameters in GraphPad Prism5.

#### Enzymatic PLA_2_ fluorescent assay

To assess PLA_2_ activity, we used the EnzChek^TM^ Phospholipase A2 Assay Kit (#E10217, Fisher Scientific, Waltham, USA), following the manufacturer’s instructions. Briefly, 0.2 μg of the four *A. contortrix* venoms were assayed in triplicate, for each experimental repeat. As a control, 1 μg samples of *C. atrox* venom were also measured, alongside a negative control containing no venom. A standard activity curve to compare venom activity against was generated using 5, 4, 3, 2, 1 and 0 U/ml of bee PLA_2_ enzyme present in the kit. Fifty microlitre samples were mixed with 50 μl of substrate mix and the reaction incubated in the dark for 10 mins. End-point fluorescence was then measured on a FLUOStar Omega Instrument at an excitation wavelength of 485 nm and an emission wavelength of 520 nm. The mean value for the negative control was subtracted from the raw values for each sample and the PLA_2_ activity calculated as (U/ml)/μg of venom, relative to the standard curve. To normalize across independent experimental repeats, the PLA_2_ activity in each sample was divided to the PLA_2_ activity of the *C. atrox* sample. The data represents the mean of triplicate independent experiments and the error bars represent SEMs.

### Data Availability Statement

Molecular sequence data generated via transcriptomic experiments are available from the National Center for Biotechnology Information (NCBI) Sequence Read Archive (SRA) (raw reads; accession: SRR2032114) and Trascriptome Shotgun Assembly (TSA) (assembly; accession: GDAY00000000.2) databases. Curated proteomic data is available in Supplementary Information Tables S1–S4. All remaining datasets generated and analysed during the current study are available from the corresponding authors on request.

## Electronic supplementary material

Supplementary Information

## References

[1] Watts PC (2006). Parthenogenesis in Komodo dragons. Nature.

[2] Chapman DD (2007). Virgin birth in a hammerhead shark. Biol. Lett..

[3] Schut E, Hemmings N, Borkhead TR (2008). Parthenogenesis in a passerine bird, the Zebra Finch *Taeniopygia guttata*. Ibis.

[4] Booth W, Schuett GW (2016). The emerging phylogenetic pattern of parthenogenesis in snakes. Biol. J. Linn. Soc..

[5] Booth W, Johnson DH, Moore S, Schal C, Vargo EL (2011). Evidence for viable, non-clonal but fatherless Boa constrictors. Biol. Lett..

[6] Dudgeon CL, Coulton L, Bone R, Ovenden JR, Thomas S (2017). Switch from sexual to parthenogenetic reproduction in a zebra shark. Sci. Rep..

[7] Booth W (2011). Consecutive virgin births in the new world boid snake, the Colombian rainbow boa. Epicrates maurus. J. Hered..

[8] Feldheim KA (2010). Shark virgin birth produces multiple, viable offspring. J. Hered..

[9] Feldheim KA (2017). Multiple births by a captive swellshark *Cephaloscyllium ventriosum* via facultative parthenogenesis. J. Fish Biol..

[10] Reynolds RG, Booth W, Schuett GW, Fitzpatrick BM, Burghardt GM (2012). Successive virgin births of viable male progeny in the checkered gartersnake. Thamnophis marcianus. Biol. J. Linn. Soc..

[11] Harmon TS, Kamerman TY, Corwin AL, Sellas AB (2016). Consecutive parthenogenetic births in a spotted eagle ray *Aetobatus narinari*. J. Fish Biol..

[12] Straube N, Lampert KP, Geiger MF, Weiβ JD, Kirchhauser JX (2016). First record of second-generation facultative parthenogenesis in a vertebrate species, the whitespotted bambooshark *Choloscyllium plagiosum*. J. Fish Biol..

[13] Lampert KP (2008). Facultative parthenogenesis in vertebrates: reproductive error or chance?. Sex. Dev..

[14] Groot TVM, Bruins E, Breeuwer JAJ (2003). Molecular genetic evidence for parthenogenesis in the Burmese python. Python molurus bivittatus. Heredity.

[15] Booth W (2014). New insights on facultative parthenogenesis in pythons. Biol. J. Linn. Soc..

[16] Olsen, M. *Avian parthenogenesis. US Dept. Agriculture, Agriculture Research Service, Beltsville*, MD, USA (1975).

[17] Booth W (2012). Facultative parthenogenesis discovered in wild vertebrates. Biol. Lett..

[18] Fields AT, Feldheim KA, Poulakis GR, Chapman DD (2015). Facultative parthenogenesis in a critically endangered wild vertebrate. Curr. Biol..

[19] van der Kooi CJ, Schwander T (2015). Parthenogenesis: birth of a new lineage or reproductive accident?. Curr. Biol..

[20] Booth W, Schuett GW (2011). Molecular genetic evidence for alternate reproductive strategies in North American pitviper (Serpentes, Viperidae): long-term sperm storage and parhenogenesis. Biol. J. Linn. Soc..

[21] Vonk FJ (2013). The king cobra genome reveals dynamic gene evolution and adaptation in the snake venom system. Proc. Natl. Acad. Sci. USA.

[22] Chippaux JP, Williams V, White J (1991). Snake venom variability: methods of study, results and interpretation. Toxicon.

[23] Massey DJ (2012). Venom variability and envenoming severity outcomes of the *Crotalus scutulatus scutulatus* (Mojave rattlesnake) from Southern Arizona. J. Proteomics.

[24] Durban J (2017). Integrated venomics and venom gland transcriptome analysis of juvenile and adult Mexican rattlesnakes *Crotalus simus*, *C. tzabcan* and *C. culminatus* revealed miRNA-modulated ontogenetic shifts. J. Proteome Res..

[25] Chippaux JP, Boche J, Courtois B (1982). Electrophoretic patterns of the venoms from a litter of *Bitis gabonica* snakes. Toxicon.

[26] Gregory-Dwyer VM, Egen NB, Bosisio AB, Righetti PG, Russell FE (1986). An isoelectric focusing study of seasonal variation in rattlesnake venom proteins. Toxicon.

[27] Daltry JC, Wüster W, Thorpe RS (1996). Diet and snake venom evolution. Nature.

[28] Daltry, J., Wuster, W. & Thorpe, R. The role of ecology in determining venom variation in the Malayan pit viper. In Venomous snakes: Ecology, evolution, and snakebite, J. Daltry, W. Wuster & R. Thorpe, ed. (Oxford, UK: Clarendon Press), pp. 155–171 (1997).

[29] Gibbs HL, Sanz L, Chiucchi JE, Farrell TM, Calvete JJ (2011). Proteomic analysis of ontogenetic and diet-related changes in venom composition of juvenile and adult Dusky Pigmy rattlesnakes (*Sistrurus miliarius barbouri*). J. Proteomics.

[30] Levine BA (2016). Population genetics of the copperhead at its most northeastern distribution. Copeia.

[31] Calvete JJ (2017). Venomics: integrative venom proteomics and beyond. Biochem. J..

[32] Lomonte B (2014). Venomics of New World pit vipers: Genus-wide comparisons of venom proteomes across *Agkistrodon*. J. Proteomics.

[33] Andrade DV, Abe AS (1999). Relationship of venom ontogeny and diet in *Bothrops*. Herpetologica.

[34] Cipriani V (2017). Correlation between ontogenetic dietary shifts and venom variation in Australian brown snakes (*Pseudonaja*). Comp. Biochem. Physiol. Part C Toxicol. Pharmacol..

[35] Rokyta DR, Margres MJ, Ward MJ, Sanchez EE (2017). The genetics of venom ontogeny in the eastern diamondback rattlesnake (*Crotalus adamanteus*). PeerJ.

[36] Fujii Y, Okuda D, Fujimoto Z, Morita T, Mizuno H (2002). Crystallization and preliminary crystallographic studies of dimeric disintegrins from the venom of two *Agkistrodon* snakes. Acta Crystallogr. Sect. D Biol. Crystallogr..

[37] Moiseeva N (2008). Structure of acostatin, a dimeric disintegrin from Southern copperhead (*Agkistrodon contortrix contortrix*), at 1.7 Å resolution. Acta Crystallogr. Sect. D Biol. Crystallogr..

[38] Margres MJ (2016). Expression differentiation is constrained to low-expression proteins over ecological timescales. Genetics.

[39] Calvete JJ (2011). Snake population venomics and antivenomics of *Bothrops atrox*: Paedomorphism along its transamazonian dispersal and implications of geographic venom variability on snakebite management. J. Proteomics.

[40] Margres MJ (2017). Quantity, not quality: rapid adaptation in a polygenic trait preoceeded exclusively through expression differentiation. Mol. Biol. Evol..

[41] Pfeifer K (2000). Mechanisms of genomic imprinting. Am. J. Hum. Genet..

[42] Casewell NR (2014). Medically important differences in snake venom composition are dictated by distinct postgenomic mechanisms. Proc. Natl. Acad. Sci. USA.

[43] Kini R, Koh C (2016). Metalloproteases affecting blood coagulation, fibrinolysis and platelet aggregation from snake venoms: Definition and nomenclature of interaction sites. Toxins (Basel).

[44] Slagboom J, Kool J, Harrison R, Casewell NR (2017). Haemotoxic snake venoms: their functional activity, impact on snakebite victims and pharmaceutical promise. Br. J. Haematol..

[45] Serrano SMT, Maroun RC (2005). Snake venom serine proteinases: sequence homology vs. substrate specificity, a paradox to be solved. Toxicon.

[46] Still K (2017). Multipurpose HTS coagulation analysis: assay development and assessment of coagulopathic snake venoms. Toxins (Basel)..

[47] van den Bergh CJ, Slotboom AJ, Verheij HM, de Haas GH (1988). The role of aspartic acid-49 in the active site of phospholipase A2. A site-specific mutagenesis study of porcine pancreatic phospholipase A2 and the rationale of the enzymatic activity of [lysine49]phospholipase A2 from *Agkistrodon piscivorus piscivorus*’ venom. Eur. J. Biochem..

[48] Ownby CL, S de Araujo HS, White SP, Fletcher JE (1999). Lysine 49 phospholipase A2 proteins. Toxicon.

[49] Allen L, Sanders KL, Thomson VA (2018). Molecular evidence for the first records of facultative parthenogenesis in elapid snakes. R. Soc. Open Sci..

[50] Margres MJ, Bigelow AT, Lemmon EM, Lemmon AR, Rokyta DR (2017). Selection to increase expression, not sequence diversity, precedes gene family origin and expansion in rattlesnake venom. Genetics.

[51] Gamble T (2017). The discovery of XY sex chromosomes in a *Boa* and *Python*. Current Biology..

[52] Smith CF, Schuett GW, Earley RL, Schwenk K (2009). The spatial and reproductive ecology of the copperhead (*Agkistrodon contortrix*) at the northeastern extreme of its range. Herpetol. Monogr..

[53] Fitch HS (1960). Autecology of the copperhead. University of Kansas Publications, Museum of Natural History.

[54] Rokyta DR, Wray KP, McGivern JJ, Margres MJ (2015). The transcriptomic and proteomic basis for the evolution of a novel venom phenotype within the Timber Rattlesnake (*Crotalus horridus*). Toxicon.

[55] Rokyta DR, Margres MJ, Calvin K (2015). Post-transcriptional mechanisms contribute little to phenotypic variation in snake venoms. G3 (Bethesda).

[56] Zhang J, Kobert K, Flouri T, Stamatakis A (2014). PEAR: a fast and accurate Illumina Paired-End reAd mergeR. Bioinformatics.

[57] Archer J, Whiteley G, Casewell NR, Harrison RA, Wagstaff SC (2014). VTBuilder: a tool for the assembly of multi isoform transcriptomes. BMC Bioinformatics.

[58] Grabherr MG (2011). Full-length transcriptome assembly from RNA-Seq data without a reference genome. Nat. Biotechnol..

[59] Rokyta DR, Lemmon AR, Margres MJ, Aronow K (2012). The venom-gland transcriptome of the eastern diamondback rattlesnake (*Crotalus adamanteus*). BMC Genomics.

[60] Langmead B, Salzberg SL (2012). Fast gapped-read alignment with Bowtie 2. Nat. Methods.

[61] Ainsworth S (2018). The paraspecific neutralisation of snake venom induced coagulopathy by antivenoms. Communications Biology..

