# Supplementary material for: Venom Complexity in a Pitviper Produced by Facultative Parthenogenesis
Source: Sci Rep. 2018 Aug 1;8:11539. doi: 10.1038/s41598-018-29791-y (PMC6070573; doi:10.1038/s41598-018-29791-y)
Supplement: Supplementary file 1 — Supplementary Information [file 41598_2018_29791_MOESM1_ESM.docx]

**SUPPLEMENTARY INFORMATION**

Venom Complexity in a Pitviper Produced by Facultative Parthenogenesis

Calvete, J.J.^1,^*^,#^, Casewell, N.R.^2,^*^,#^, Hernández-Guzmán, U.^1,3^, Quesada-Bernat, S.^1^, Sanz, L.^1^, Rokyta D.R.^4^, Storey, D.^2,5^, Albulescu, L-O.^2^, Wüster, W.^5,6^, Smith, C.F.^6,7,8^, Schuett, G.W.^6,7,9^ & Booth, W.^6,10^

^1^ Evolutionary and Translational Venomics Laboratory, CSIC, Valencia, Spain

^2^ Alistair Reid Venom Research Unit, Parasitology Department, Liverpool School of Tropical Medicine, Pembroke Place, Liverpool, L3 5QA, UK

^3^ Laboratorio de Química de Biomacromoléculas, Instituto de Química, Universidad Nacional Autónoma de México, Ciudad Universitaria, Delegación Coyoacán C.P. 04510, Ciudad de México, Mexico.

^4^ Department of Biological Science, Florida State University, Tallahassee, FL, USA

^5^ Molecular Ecology and Fisheries Genetics Laboratory, School of Biological Sciences, Environment Centre Wales, Bangor University, Bangor, LL57 2UW, UK

^6^ Chiricahua Desert Museum, P.O. Box 376, Rodeo, NM, USA

^7^ The Copperhead Institute, P.O. Box 6755, Spartanburg, SC, USA

^8^ Department of Biology, Wofford College, 429 North Church Street, Spartanburg, SC, USA

^9^ Department of Biology and Neuroscience Institute, Georgia State University, Atlanta, GA, USA

^10^ Department of Biological Science, The University of Tulsa, Tulsa, OK, USA

^#^ these authors contributed equally.

* Corresponding authors.

- Juan J. Calvete. Email: [jcalvete@ibv.csic.es](mailto:jcalvete@ibv.csic.es)
- Nicholas R. Casewell. Email: [nicholas.casewell@lstmed.ac.uk](mailto:nicholas.casewell@lstmed.ac.uk)

**Supplementary Figures**





**Fig. S1. Functional comparisons of venom activity between mother, parthenogen and unrelated female and male individuals. A)** The thrombin-like enzyme activity of the four *A. contortrix* venoms in comparison with a positive (0.1 Units of thrombin) and negative (substrate-only) control. The graphs show the mean absorbance of triplicate measurements from three independent experiments over 30 mins at two venom concentrations. Error bars are only displayed for every third reading to aid visualisation, and represent SEM. **B)** The procoagulant activity of the four *A. contortrix* venoms in comparison with a plasma-only negative control. The graphs show the mean absorbance of triplicate measurements over 40 mins at two venom concentrations. Error bars represent SEM. **C)** Enzymatic PLA_2_ activity of the four *A. contortrix* venoms plotted against (i) total PLA_2_ abundance in venom, (ii) enzymatic PLA_2_ abundance in venom (PLA_2_ toxins with an aspartic acid residue at position 49) and (iii) non-enzymatic PLA_2_ abundance in venom (PLA_2_ toxins with a lysine residue at position 49), as detected by proteomic measurements.

**
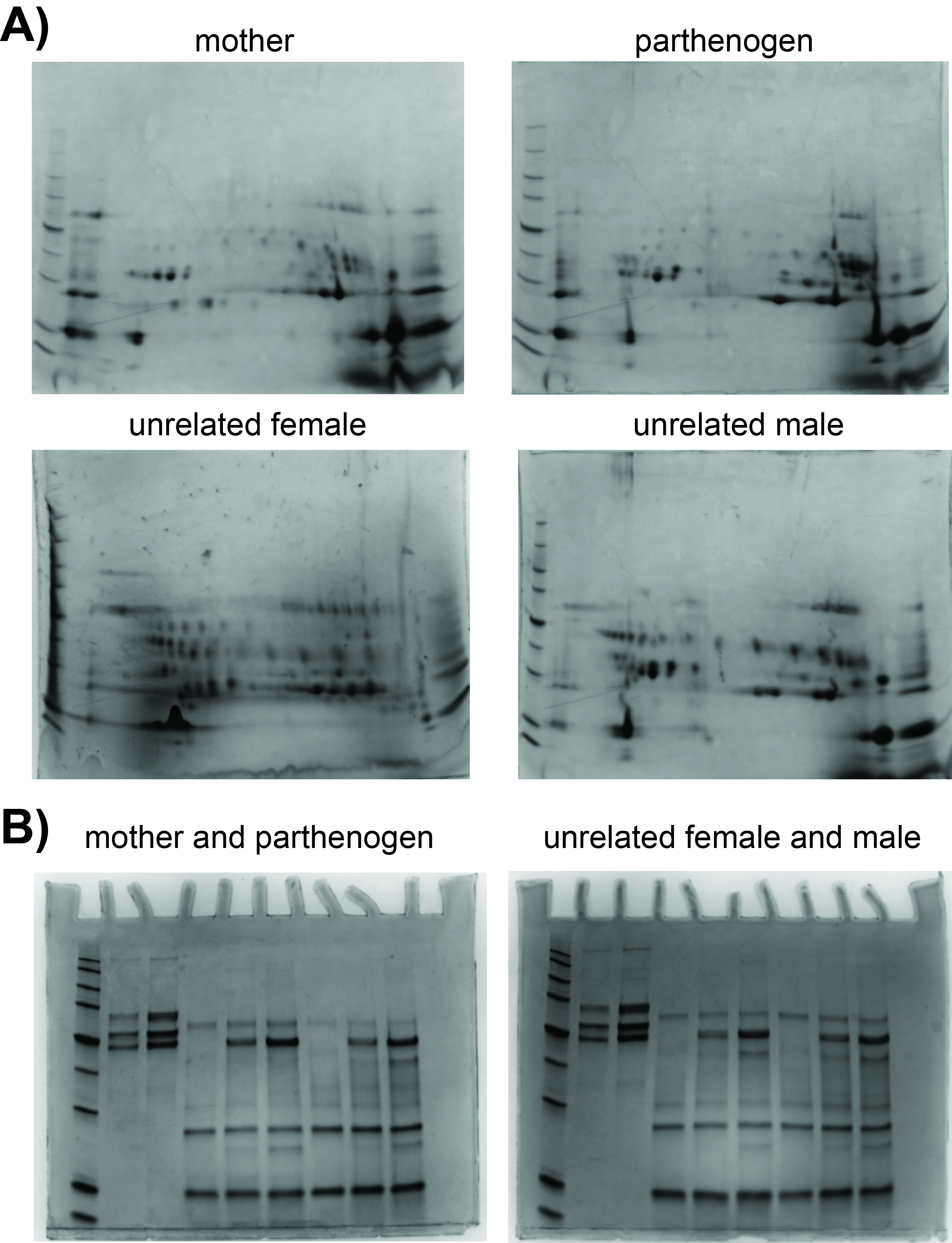
**

**Fig. S2. Original gel images for those included in the manuscript in Figs 2 and 5. A)** Original images for the 2D SDS-PAGE gel electrophoretic profiles of each of the four venoms (annotated in Fig. 2). **B)** Original images for the 1D SDS-PAGE gel electrophoretic profiles of the fibrinogen degradation experiments (annotated in Fig. 5).

**Supplementary Tables**

**Table S1.** Identification by MS/MS of the venom components of *Agkistrodon c. contortrix* specimen F204 (parthenogen mother) (Fig. 2B). *, ambiguous ID.

| **Spot ID** | **%** | **MW (kDa)** | **ESI-MS (ave)** | **m/z** | **z** | **Peptide sequence** | **Transcriptome match** | **Protein family** | **Observations** |
| --- | --- | --- | --- | --- | --- | --- | --- | --- | --- |
|  |  |  |  |  |  |  |  |  |  |
| **2** | 1.06 |  |  | 532.2 | 2 | TPPAGPDVGPR | Acont_BPP-1a_98 | VAP (BIP) |  |
| **4** | 0.78 | 8^▼^ |  | 598.8 | 2 | NPCCDAATCK | Acont_DIS-2a_e351 | Dimeric disintegrin | Acostatin alpha (Q805F7) |
|  |  |  |  | 992.4 | 2 | LTPGSQCAEGLCCDQCK |  |  |  |
|  |  |  |  | 569.2 | 2 | CTGQSGDCPR |  |  |  |
| **5** | 1.23 |  |  | 430.2 | 1 | ZNW | Acont_BPP-1a_98 | SVMPi |  |
| **6** | 0.74 |  |  | 444.2 | 1 | ZBW | Acont_BPP-1a_98 | SVMPi |  |
| **9** | 0.83 | 21▪ | 13487,8/13237,7/13352,7 | 598.8 | 2 | NPCCDAATCK | Acont_DIS-2a_e351 | Dimeric disintegrin | Acostatin alpha (Q805F7) |
|  |  |  |  | 992.4 | 2 | LTPGSQCAEGLCCDQCK |  |  |  |
|  |  |  |  | 569.2 | 2 | CTGQSGDCPR |  |  |  |
| **10** | 0.47 | 21▪ | 13783,9/13636,8/13521,8 | 987.4 | 2 | LTTGSQCADGLCCDQCK | Acont_SVMPII-5a_e416 | Dimeric disintegrin | Acostatin beta (BAC55945) |
|  |  |  |  | 942.9 | 2 | GDDLDDYCNGISAGCPR |  |  |  |
|  |  |  |  | 992.4 | 2 | LTPGSQCAEGLCCDQCK | Acont_DIS-2a_e351 | Dimeric disintegrin | Acostatin alpha (Q805F7) |
|  |  |  |  | 569.2 | 2 | CTGQSGDCPR |  |  |  |
|  |  | 8^▼^ |  | 987.4 | 2 | LTTGSQCADGLCCDQCK | Acont_SVMPII-5a_e416 | Dimeric disintegrin | Acostatin beta (BAC55945) |
|  |  |  |  | 942.9 | 2 | GDDLDDYCNGISAGCPR |  |  |  |
| **11** | 0.19 |  | 13216,7/13100,6 | 598.8 | 2 | NPCCDAATCK | Acont_DIS-2a_e351 | Dimeric disintegrin | Acostatin alpha (Q805F7) |
|  |  |  |  | 569.2 | 2 | CTGQSGDCPR |  |  |  |
| **12** | 0.49 |  |  | 534.8 | 2 | WPPHPQIPP | Acont_BPP-1a_98 | VAP (BPP) |  |
|  |  |  |  | 598.8 | 2 | NPCCDAATCK | Acont_DIS-2a_e351 | Dimeric disintegrin |  |
|  |  |  |  | 569.2 | 2 | CTGQSGDCPR |  |  |  |
| **15** | 1.09 | 31^▼^ |  | 460.3 | 2 | MILQETGK | Acont_PLA2-3a_e604 | PLA2 |  |
|  |  |  |  | 498.7 | 2 | ENLDTYNK |  |  |  |
|  |  |  | 14042.4 | 416.2 | 2 | YNPYFK |  |  |  |
|  | 20.67 | 14^▼^ |  | 562.8 | 2 | ENLDTYNKK | Acont_PLA2-3a_e604 | PLA2 | Mav calc: 14043.3 |
|  |  |  |  | 460.3 | 2 | MILQETGK | Acont_PLA2-3a_e604 | PLA2 |  |
|  |  |  |  | 707.3 | 3 | (NAIT)SYGFYGCNCGWGHR |  |  |  |
|  |  |  |  | 454.6 | 3 | (AII)CEEKNPCK |  |  |  |
|  |  |  |  | 585.6 | 3 | (EMC)ECDKAVAICLR |  |  |  |
| **16** | 0.86 | 16 ^∎^ |  | 460.3 | 2 | MILQETGK | Acont_PLA2-3a_e604 | PLA2 |  |
|  |  |  |  | 707.3 | 3 | (NAIT)SYGFYGCNCGWGHR |  |  |  |
|  |  |  |  | 423.7 | 2 | LTNCNPK |  |  |  |
|  |  |  |  | 431.7 | 2 | AIICEEK |  |  |  |
|  |  |  |  | 401.7 | 2 | AVAICLR |  |  |  |
|  |  |  |  | 498.7 | 2 | ENLDTYNK |  |  |  |
|  |  |  |  | 562.8 | 2 | ENLDTYNKK |  |  |  |
|  |  |  |  | 480.3 | 2 | KYNPYFK |  |  |  |
|  |  |  | 14042.4 | 417.2 | 2 | YNPYFK |  |  |  |
|  | 0.70 | 14 ^∎^ |  | 707.3 | 3 | (649.3)GFYGCNCGWGHR | Acont_PLA2-3a_e604 | PLA2 |  |
|  |  |  |  | 585.6 | 3 | (EMCEC)DKAVAICLR |  |  |  |
|  |  |  |  | 468.2 | 2 | MoxILQETGK |  |  |  |
|  |  |  |  | 431.7 | 2 | AIICEEK |  |  |  |
|  |  |  |  | 401.7 | 2 | AVAICLR |  |  |  |
|  |  |  |  | 498.7 | 2 | ENLDTYNK |  |  |  |
| **18** | 0.97 | 33^▼^ |  | 565.3 | 2 | FLVALYTFR | Acont_SVSP-5_e565 | SVSP |  |
|  |  |  |  | 500.3 | 2 | EKFFCLR |  |  |  |
|  |  |  |  | 595.3 | 2 | WDKDIMLIR |  |  |  |
|  |  |  |  | 721.7 | 3 | STHIAPLSLPSSPPSLGSVCR |  |  |  |
|  |  |  |  | 536.9 | 3 | TLCAGILEGGKDSCK |  |  |  |
|  | 0.22 | 31^▼^ | 14213.5/14195.5/14180.4 | 507.8 | 2 | AAYPQLPVR | Acont_SVSP-5_e565 | SVSP |  |
|  | 0.73 | 16^▼^ |  | 460.3 | 2 | MILQETGK | Acont_PLA2-3a_e604 | PLA2 |  |
|  | 0.24 | 16^▼^ |  | 565.3 | 2 | FLVALYTFR | Acont_SVSP-5_e565 | SVSP |  |
|  |  |  |  | 721.7 | 3 | STHIAPLSLPSSPPSLGSVCR |  |  |  |
|  |  |  |  | 507.8 | 2 | AAYPQLPVR |  |  |  |
| **19** | 0.31 | 36^▼^ |  | 727.4 | 2 | AAYPEYDLPATSR | Acont_SVSP-15a_e914 | SVSP |  |
|  |  |  |  | 559.8 | 2 | TLCAGILEGGK |  |  |  |
|  |  |  |  | 1036.5 | 2 | VMGWGTTTSPQETLPDVPR | Acont_SVSP-12a_98-10M126 | SVSP |  |
|  |  |  |  | 519.3 | 2 | AFYPGLLEK |  |  |  |
|  |  |  |  | 427.6 | 3 | AFYPGLLEKSR |  |  |  |
|  | 0.13 | 36^▼^ |  | 551.3 | 2 | LDIYTYSVK | Acont_PLA2-1a_e343 | PLA2 |  |
|  |  |  |  | 490.7 | 2 | QICECDR |  |  |  |
|  |  |  |  | 404.7 | 2 | AAAICFR |  |  |  |
|  | 0.12 | 33^▼^ |  | 565.3 | 2 | FLVALYTFR | Acont_SVSP-5_e565 | SVSP |  |
|  |  |  |  | 507.8 | 2 | AAYPQLPVR |  |  |  |
|  |  |  |  | 559.8 | 2 | TLCAGILEGGK |  |  |  |
|  |  |  |  | 727.4 | 2 | AAYPEYDLPATSR | Acont_SVSP-15a_e914 | SVSP |  |
|  |  |  |  | 551.3 | 2 | LDIYTYSVK | Acont_PLA2-1a_e343 | PLA2 |  |
|  | 0.10 | 33^▼^ |  | 490.7 | 2 | QICECDR |  |  |  |
|  |  |  |  | 603.8 | 2 | DVVCGGTNPCK |  |  |  |
|  | 0.43 | 31^▼^ |  | 551.3 | 2 | LDIYTYSVK | Acont_PLA2-1a_e343 | PLA2 |  |
|  |  |  |  | 490.7 | 2 | QICECDR |  |  |  |
|  | 1.53 | 16^▼^ |  | 551.3 | 2 | LDIYTYSVK | Acont_PLA2-1a_e343 | PLA2 |  |
|  |  |  |  | 603.8 | 2 | DVVCGGTNPCK |  |  |  |
|  |  |  |  | 404.7 | 2 | AAAICFR |  |  |  |
|  |  |  |  | 502.5 | 3 | CCFVHDCCYGK | Acont_PLA2-4_e312 | PLA2 |  |
|  |  |  |  | 491.2 | 2 | EICECDR |  |  |  |
|  |  |  |  | 460.3 | 2 | MILQETGK | Acont_PLA2-3a_e604 | PLA2 |  |
|  | 0.10 | 16^▼^ | 14163.8/13768.9 | 500.2 | 3 | VVGGDECNINEHR | Acont_SVSP-3a_e(242,9,339,515) | SVSP | * |
|  | 8.15 | 14^▼^ |  | 551.3 | 2 | LDIYTYSVK | Acont_PLA2-1a_e343 | PLA2 |  |
|  |  |  |  | 490.7 | 2 | QICECDR |  |  |  |
| **20** | 0.05 | 36^▼^ |  | 608.3 | 2 | KVPNEDEQTR | Acont_SVSP-15a_e914 | SVSP |  |
|  |  |  |  | 544.3 | 2 | VPNEDEQTR |  |  |  |
|  |  |  |  | 727.4 | 2 | AAYPEYDLPATSR |  |  |  |
|  |  |  |  | 559.8 | 2 | TLCAGILEGGK |  |  |  |
|  |  |  |  | 571.8 | 2 | FLVALYNFR | Acont_SVSP-14a_e824 | SVSP |  |
|  |  |  |  | 608.3 | 2 | KVPNEDEQTR |  |  |  |
|  |  |  |  | 544.3 | 2 | VPNEDEQTR |  |  |  |
|  |  |  |  | 559.8 | 2 | TLCAGILEGGK |  |  |  |
|  | 0.34 | 34^▼^ |  | 486.3 | 2 | IYLGVHNR | Acont_SVSP-11a_e515(339) | SVSP | * |
|  |  |  |  | 553.6 | 3 | NNIKWDKDIMLIR |  |  |  |
|  |  |  |  | 753.4 | 3 | NSAHIAPHSLPSNAPSVGSVCR |  |  |  |
|  |  |  |  | 766.4 | 2 | GLAATTLCAGILEGGK | Acont_SVSP-16a_e(369,982) | SVSP | * |
|  |  |  |  | 571.8 | 2 | FLVALYNFR | Acont_SVSP-14a_e824 | SVSP |  |
|  |  |  |  | 608.3 | 2 | KVPNEDEQTR |  |  |  |
|  |  |  |  | 550.9 | 3 | TLCAGILEGGKDTCR |  |  |  |
|  |  |  |  | 727.4 | 2 | AAYPEYDLPATSR | Acont_SVSP-15a_e914 | SVSP |  |
|  | 0.96 | 31^▼^ |  | 654.3 | 2 | NFQMLFGVHSK | Acont_SVSP-2a_e957 | SVSP |  |
|  |  |  |  | 559.3 | 2 | ILNEDEQTR |  |  |  |
|  |  |  |  | 403.7 | 2 | FICPNR |  |  |  |
|  |  |  |  | 497.6 | 3 | DDEKDKDIMLIR |  |  |  |
|  |  |  |  | 604.8 | 2 | IMoxGWGTTTPTK |  |  |  |
|  |  |  |  | 624.3 | 4 | ETYPDVPHCANINLLDHAVCR |  |  |  |
|  |  |  |  | 751.4 | 3 | AAYPELLAESSTLCAGTQQGGK |  |  |  |
|  |  |  |  | 405.9 | 3 | KVPNEDEQTR |  |  |  |
|  |  |  |  | 860.1 | 3 | ETYPDVPHCANINLLDHAVCR |  |  |  |
|  |  |  |  | 727.4 | 2 | AAYPEYDLPATSR | Acont_SVSP-15a_e914 | SVSP |  |
|  |  |  |  | 559.8 | 2 | TLCAGILEGGK |  |  |  |
|  |  |  |  | 571.8 | 2 | FLVALYNFR | Acont_SVSP-14a_e824 | SVSP |  |
|  |  |  |  | 565.3 | 2 | FLVALYTFR | Acont_SVSP-5_e565 | SVSP |  |
|  |  |  |  | 595.3 | 2 | WDKDIMLIR |  |  |  |
|  |  |  |  | 507.8 | 2 | AAYPQLPVR |  |  |  |
|  |  |  |  | 500.2 | 3 | VVGGDECNINEHR | Acont_SVSP-3a_e(242,9,339,515) | SVSP | * |
|  |  |  |  | 766.4 | 2 | GLAATTLCAGILEGGK | Acont_SVSP-16a_e(369,982) | SVSP | * |
|  | 0.05 | 21^▼^ |  | 596.8 | 2 | IMGWGTTTPTK | Acont_SVSP-2a_e957 | SVSP |  |
|  |  |  |  | 766.4 | 2 | GLAATTLCAGILEGGK | Acont_SVSP-16a_e(369,982) | SVSP | * |
|  |  |  |  | 401.2 | 3 | LLDKDIMLIK | Acont_SVSP-4_e9 | SVSP |  |
|  |  |  | 14163.8/13768.9 | 500.2 | 3 | VVGGDECNINEHR | Acont_SVSP-3a_e(242,9,339,515) | SVSP | * |
|  | 1.93 | 14^▼^ |  | 551.3 | 2 | LDIYTYSVK | Acont_PLA2-1a_e343 | PLA2 | Mave calc: 14164,1 |
|  |  |  |  | 401.7 | 2 | AVAICLR | Acont_PLA2-3a_e604 | PLA2 |  |
|  | 1.44 | 14^▼^ |  | 486.3 | 2 | IYLGVHNR | Acont_SVSP-16a_e(369,339,515) | SVSP | * |
|  | 0.04 | 6^▼^ |  | 551.3 | 2 | LDIYTYSVK | Acont_PLA2-1a_e343 | PLA2 |  |
|  |  |  |  | 490.7 | 2 | QICECDR |  |  |  |
|  |  |  |  | 404.7 | 2 | AAAICFR |  |  |  |
|  | 0.01 | 6^▼^ |  | 500.2 | 3 | VVGGDECNINEHR | Acont_SVSP-3a_e(242,9,339,515) | SVSP | * |
|  |  |  |  | 486.3 | 2 | IYLGVHNR | Acont_SVSP-16a_e(369,339,515) | SVSP | * |
| **21** | 0.03 | 36^▼^ |  | 571.8 | 2 | FLVALYNFR | Acont_SVSP-14a_e824 | SVSP |  |
|  |  |  |  | 608.3 | 2 | KVPNEDEQTR |  |  |  |
|  |  |  |  | 444.7 | 2 | FFCLSSK |  |  |  |
|  |  |  |  | 559.8 | 2 | TLCAGILEGGK |  |  |  |
|  |  |  |  | 550.9 | 3 | TLCAGILEGGKDTCR |  |  |  |
|  |  |  |  | 727.4 | 2 | AAYPEYDLPATSR | Acont_SVSP-15a_e914 | SVSP |  |
|  |  |  |  | 559.8 | 2 | TLCAGILEGGK |  |  |  |
|  |  |  |  | 750.7 | 3 | NSAHIAPLSLPSNSPSVGSVCR | Acont_SVSP-18a_e982 | SVSP |  |
|  |  |  |  | 486.3 | 2 | IYLGVHNR | Acont_SVSP-16a_e(369,339,515) | SVSP | * |
|  | 0.23 | 21^▼^ |  | 565.3 | 4 | NSAHIAPHSLPSNAPSVGSVCR | Acont_SVSP-11a_e515 | SVSP |  |
|  |  |  |  | 403.7 | 2 | KPGLYTK |  |  |  |
|  |  |  |  | 486.3 | 2 | IYLGVHNR | Acont_SVSP-16a_e369 | SVSP |  |
|  |  |  |  | 766.4 | 2 | GLAATTLCAGILEGGK |  |  |  |
|  |  |  |  | 401.2 | 3 | LLDKDIMLIK | Acont_SVSP-4_e9 | SVSP |  |
|  |  |  | 14163.8 | 559.8 | 2 | TLCAGILEGGK |  |  |  |
|  | 1.13 | 14^▼^ |  | 521.5 | 3 | CCFVHDCCYNK | Acont_PLA2-1a_e343 | PLA2 |  |
|  |  |  |  | 551.3 | 2 | LDIYTYSVK |  |  |  |
|  |  |  |  | 416.2 | 2 | YNPYFK | Acont_PLA2-3a_e604 | PLA2 |  |
|  |  |  |  | 460.3 | 2 | MILQETGK |  |  |  |
|  | 0.03 | 14^▼^ |  | 595.3 | 2 | WDKDIMLIR | Acont_SVSP-12a_98-10M126(M191,M279,M326..) | SVSP | * |
|  | 1.16 | 6^▼^ |  | 500.2 | 3 | VVGGDECNINEHR | Acont_SVSP-3a_e(242,9,339,515) | SVSP | * |
|  |  |  |  | 486.3 | 2 | IYLGVHNR | Acont_SVSP-16a_e(369,339,515) | SVSP | * |
|  |  |  |  | 504.9 | 3 | VVGGDECNINEHR | Acont_SVSP-3a_e(242,9,339,515) | SVSP | * |
| **22** | 0.54 | 36^▼^ |  | 830.7 | 3 | SLCCGTLINQEWVLSAAHCDR | Acont_SVSP-16a_e369 | SVSP |  |
|  |  |  |  | 487.2 | 2 | YFCLNTR |  |  |  |
|  |  |  |  | 444.7 | 2 | FFCLSSK | Acont_SVSP-14a_e(824,857,914,10M191) | SVSP | * |
|  |  |  |  | 559.8 | 2 | TLCAGILEGGK | Acont_SVSP-3a_e(242,9,339,515) | SVSP | * |
|  |  |  |  | 681.4 | 2 | SRTLCAGILEGGK | Acont_SVSP-14a_e(824,857,914,10M191..) | SVSP | * |
|  | 0.01 | 26^▼^ |  | 490.7 | 2 | QICECDR | Acont_PLA2-4_e312 | PLA2 |  |
|  | 0.03 | 26^▼^ |  | 500.2 | 3 | VVGGDECNINEHR | Acont_SVSP-3a_e(242,9,339,515) | SVSP | * |
|  | 0.04 | 21^▼^ |  | 437.7 | 2 | FFCVSSK | Acont_SVSP-8_e14 | SVSP |  |
|  |  |  |  | 766.4 | 2 | GLAATTLCAGILEGGK |  |  |  |
|  | 2.81 | 16^▼^ | 13753.0 | 404.7 | 2 | AAAICFR | Acont_PLA2-1a_e343 | PLA2 |  |
|  |  |  |  | 753.3 | 2 | CCFVHDCCYGK | Acont_PLA2-4_e312 | PLA2 | Mave calc: 13753,4 |
|  | 0.05 | 16^▼^ |  | 500.2 | 3 | VVGGDECNINEHR | Acont_SVSP-3a_e(242,9,339,515) | SVSP | * |
|  | 0.07 | 14^▼^ |  | 404.7 | 2 | AAAICFR | Acont_PLA2-1a_e343 | PLA2 |  |
|  | 0.00 | 14^▼^ |  | 595.3 | 2 | AIVFDEGIIGR | Acont_SVMPI-3a_e522 | PI-SVMP |  |
|  | 0.03 | 6^▼^ |  | 486.3 | 2 | IYLGVHNR | Acont_SVSP-16a_e(369,339,515) | SVSP | * |
|  |  |  |  | 494.3 | 2 | AAYPELPVK | Acont_SVSP-8_e14 | SVSP |  |
|  |  |  |  | 500.2 | 3 | VVGGDECNINEHR | Acont_SVSP-3a_e(242,9,339,515) | SVSP | * |
|  | 0.01 | 6^▼^ |  | 404.7 | 2 | AAAICFR | Acont_PLA2-1a_e343 | PLA2 |  |
| **23** | 0.06 | 45^▼^ |  | 773.1 | 3 | NSEHIAPLSLPSSPPIVGSVCR | Acont_SVSP-13a_e537 | SVSP |  |
|  |  |  |  | 803.4 | 2 | ILCAGVLEGGIDTCK |  |  |  |
|  |  |  |  | 559.8 | 2 | TLCAGILEGGK | Acont_SVSP-14a_e(824,857,914,10M191..) | SVSP | * |
|  |  |  |  | 549.3 | 2 | FLALVYTDR | Acont_SVSP-8_e14 | SVSP |  |
|  |  |  |  | 728.4 | 2 | SLPSSPPIVGSVCR | Acont_SVSP-6_98-10M191 | SVSP |  |
|  |  |  |  | 830.7 | 3 | FLCGGTLINQEWVLTAAHCDGK |  |  |  |
|  | 1.75 | 31^▼^ |  | 494.3 | 2 | AAYPELPVK | Acont_SVSP-17a_e339 | SVSP |  |
|  |  |  |  | 486.3 | 2 | IYLGVHNR |  |  |  |
|  |  |  |  | 828.9 | 2 | RLPATTLCAGILEGGK | Acont_SVSP-16a_e369 | SVSP |  |
|  |  |  |  | 487.2 | 2 | YFCLNTR |  |  |  |
|  |  |  |  | 952.8 | 3 | GDSGGPLICNGQFQGILSVGGNPCAQPR | Acont_SVSP-16a_e369 | SVSP |  |
|  |  |  |  | 500.2 | 3 | VVGGDECNINEHR | Acont_SVSP-3a_e(242,9,339,515) | SVSP | * |
|  | 3.49 | 28^▼^ |  | 559.8 | 2 | TLCAGILEGGK | Acont_SVSP-8_e14 | SVSP |  |
|  |  |  |  | 549.3 | 2 | FLALVYTDR |  |  |  |
|  |  |  |  | 830.7 | 3 | FLCGGTLINQEWVLTAAHCDGK |  |  |  |
|  |  |  |  | 725.9 | 4 | LDRPVSNSAHIAPLSLPSSPPSVGSVCR |  |  |  |
|  |  |  |  | 595.8 | 2 | IMGWGTISPTK |  |  |  |
|  |  |  |  | 833.1 | 3 | VILPDVPHCANINLLNYSECR |  |  |  |
|  |  |  |  | 494.3 | 2 | AAYPELPVK | Acont_SVSP-4_e9 | SVSP |  |
|  |  |  |  | 532.9 | 3 | KLLNEDEQIRNPK |  |  |  |
|  |  |  |  | 565.3 | 2 | LLNEDEQIR |  |  |  |
|  |  |  |  | 490.3 | 3 | LLNEDEQIRNPK |  |  |  |
|  |  |  |  | 401.2 | 3 | LLDKDIMLIK | Acont_SVSP-18a_e982 | SVSP |  |
|  |  |  |  | 750.7 | 3 | NSAHIAPLSLPSNSPSVGSVCR |  |  |  |
|  |  |  |  | 440.2 | 3 | EWDKDIMLIR | Acont_SVSP-1_e857(10M326) | SVSP | * |
|  | 0.29 | 21^▼^ |  | 549.3 | 2 | FLALVYTDR | Acont_SVSP-8_e14 | SVSP |  |
|  |  |  |  | 830.7 | 3 | FLCGGTLINQEWVLTAAHCDGK |  |  |  |
|  |  |  |  | 725.9 | 4 | LDRPVSNSAHIAPLSLPSSPPSVGSVCR |  |  |  |
|  |  |  |  | 595.8 | 2 | IMGWGTISPTK | Acont_SVSP-3a_e242 | SVSP |  |
|  |  |  |  | 500.2 | 3 | VVGGDECNINEHR |  |  |  |
|  |  |  |  | 559.8 | 2 | TLCAGILEGGK | Acont_SVSP-4_e9 | SVSP |  |
|  |  |  |  | 532.9 | 3 | KLLNEDEQIRNPK |  |  |  |
|  |  |  |  | 565.3 | 2 | LLNEDEQIR |  |  |  |
|  |  |  |  | 490.3 | 3 | LLNEDEQIRNPK |  |  |  |
|  |  |  |  | 401.2 | 3 | LLDKDIMLIK |  | SVSP |  |
|  | 0.06 | 14^▼^ |  | 750.9 | 2 | LPATTLCAGILEGGK | Acont_SVSP-17a_e339 | SVSP |  |
|  |  |  |  | 431.7 | 2 | AIICEEK | Acont_PLA2-3a_e604 | PLA2 |  |
|  | 0.11 | 14^▼^ |  | 404.7 | 2 | AAAICFR | Acont_PLA2-1a_e343(312,1f2202) | PLA2 | * |
|  |  |  |  | 490.3 | 3 | LLNEDEQIRNPK | Acont_SVSP-4_e9 | SVSP |  |
|  |  |  |  | 401.2 | 3 | LLDKDIMLIK |  |  |  |
|  |  |  |  | 559.8 | 2 | TLCAGILEGGK |  | SVSP |  |
|  |  |  |  | 549.3 | 2 | FLALVYTDR | Acont_SVSP-8_e14 | SVSP |  |
|  | 0.06 | 6^▼^ |  | 486.3 | 2 | IYLGVHNR | Acont_SVSP-16a_e(369,339,515) | SVSP | * |
|  |  |  |  | 750.9 | 2 | LPATTLCAGILEGGK | Acont_SVSP-17a_e339 | SVSP |  |
|  |  |  |  | 500.2 | 3 | VVGGDECNINEHR | Acont_SVSP-3a_e(242,9,339,515) | SVSP | * |
|  |  |  |  | 559.8 | 2 | TLCAGILEGGK | Acont_SVSP-14a_e(824,857,914,10M191..) | SVSP | * |
| **24** | 0.13 | 40^▼^ |  | 494.3 | 2 | AAYPELPVK | Acont_SVSP-13a_e537 | SVSP |  |
|  |  |  |  | 773.1 | 3 | NSEHIAPLSLPSSPPIVGSVCR |  |  |  |
|  |  |  |  | 803.4 | 2 | ILCAGVLEGGIDTCK |  |  |  |
|  |  |  |  | 587.9 | 3 | ILCAGVLEGGIDTCKR |  |  |  |
|  |  |  |  | 490.3 | 3 | LLNEDEQIRNPK | Acont_SVSP-4_e9 | SVSP |  |
|  |  |  |  | 401.2 | 3 | LLDKDIMLIK |  |  |  |
|  | 0.04 | 31^▼^ |  | 601.3 | 2 | NVPNEDEQTR | Acont_SVSP-6_98-10M191 | SVSP |  |
|  |  |  |  | 559.8 | 2 | TLCAGILEGGK |  |  |  |
|  |  |  |  | 595.3 | 2 | WDKDIMLIR |  |  |  |
|  |  |  |  | 559.8 | 2 | TLCAGILEGGK | Acont_SVSP-14a_e(824,857,914,10M191..) | SVSP | * |
|  |  |  |  | 486.3 | 2 | IYLGVHNR | Acont_SVSP-16a_e(369,339,515) | SVSP | * |
|  |  |  |  | 750.9 | 2 | LPATTLCAGILEGGK | Acont_SVSP-17a_e339 | SVSP |  |
|  |  |  |  | 500.2 | 3 | VVGGDECNINEHR | Acont_SVSP-3a_e(242,9,339,515) | SVSP | * |
|  | 3.59 | 28^▼^ |  | 565.3 | 2 | LLNEDEQIRNPK | Acont_SVSP-4_e9 | SVSP |  |
|  |  |  |  | 629.3 | 2 | KLLNEDEQIR |  |  |  |
|  |  |  |  | 532.9 | 3 | KLLNEDEQIRNPK |  |  |  |
|  |  |  |  | 565.3 | 2 | LLNEDEQIRNPK |  |  |  |
|  |  |  |  | 490.3 | 3 | LLNEDEQIRNPK |  |  |  |
|  |  |  |  | 401.2 | 3 | LLDKDIMLIK |  |  |  |
|  |  |  |  | 559.8 | 2 | TLCAGILEGGK | Acont_SVSP-8_e14 | SVSP |  |
|  |  |  |  | 830.7 | 3 | FLCGGTLINQEWVLTAAHCDGK |  |  |  |
|  |  |  |  | 437.7 | 2 | FFCVSSK |  |  |  |
|  |  |  |  | 595.8 | 2 | IMGWGTISPTK |  |  |  |
|  |  |  |  | 833.1 | 3 | (VILPDV)PHCANINLLNYSECR |  |  |  |
|  |  |  |  | 494.3 | 2 | AAYPELPVK |  |  |  |
|  | 0.04 | 21^▼^ |  | 500.2 | 3 | VVGGDECNINEHR | Acont_SVSP-3a_e(242,9,339,515) | SVSP | * |
|  |  |  |  | 403.7 | 2 | FICPNR | Acont_SVSP-7_e420(957) | SVSP | * |
|  |  |  |  | 972.8 | 3 | LDSPVNNSEHIAPLSLPSSPPSVGSVCR | Acont_SVSP-7_e420 | SVSP |  |
|  |  |  |  | 559.8 | 2 | TLCAGILEGGK | Acont_SVSP-8_e14 | SVSP |  |
|  |  |  |  | 549.3 | 2 | FLALVYTDR | Acont_SVSP-8_e14 | SVSP |  |
|  |  |  |  | 437.7 | 2 | FFCVSSK |  |  |  |
|  |  |  | 13941.5 | 750.9 | 2 | LPATTLCAGILEGGK | Acont_SVSP-17a_e339 | SVSP |  |
|  | 0.37 | 14^▼^ |  | 559.8 | 2 | TLCAGILEGGK | Acont_SVSP-14a_e(824,857,914,10M191..) | SVSP | * |
|  |  |  |  | 430.3 | 2 | KTDLLNR | Acont_SVMPI-3a_e522(1M61) | SVMP | * |
|  | 0.05 | 14^▼^ |  | 723.7 | 3 | SHDNAQLLTAIVFDEGIIGR | Acont_SVMPI-3a_e522 | SVMP |  |
|  |  |  |  | 643.8 | 2 | SAGQLYEESFR | Acont_LAAO-1_e495 | LAAO |  |
| **27** | 3.51 | 60^▼^ |  | 524.6 | 3 | DPGLLEYPVKPSEK |  |  |  |
|  |  |  |  | 532.7 | 2 | NPLEECFR |  |  |  |
|  |  |  |  | 757.8 | 2 | ETDYEEFLEIAR |  |  |  |
|  |  |  |  | 1014.2 | 3 | VVIVGAGMSGLSAAYVLAGAGHQVTVLEASGR |  |  |  |
|  |  |  |  | 532.3 | 3 | DKEDWYANLGPMR |  |  |  |
|  |  |  |  | 524.6 | 3 | DPGLLEYPVKPSEK |  |  |  |
|  |  |  |  | 643.8 | 2 | SAGQLYEESFR |  |  |  |
|  |  |  |  | 562.3 | 2 | HDDIFGYEK |  |  |  |
|  |  |  |  | 555.8 | 2 | FDEIVGGMDK |  |  |  |
|  |  |  |  | 557.8 | 2 | VIEIQQNDR |  |  |  |
|  |  |  |  | 583.3 | 2 | IKFEPPLPPK |  |  |  |
|  |  |  |  | 441.7 | 2 | IFLTCTK |  |  |  |
|  |  |  |  | 472.9 | 3 | KFWEEEGIHGGK |  |  |  |
|  | 0.66 | 45^▼^ |  | 571.3 | 3 | DCGDIVINDLSLIHK | Acont_LAAO-1_e495 | LAAO |  |
|  |  |  |  | 757.8 | 2 | ETDYEEFLEIAR |  |  |  |
|  |  |  |  | 643.8 | 2 | DPGLLEYPVKPSEK |  |  |  |
|  |  |  |  | 439.2 | 2 | YDTYSTK |  |  |  |
|  |  |  |  | 555.8 | 2 | FDEIVGGMDK |  |  |  |
|  |  |  |  | 583.4 | 2 | IKFEPPLPPK |  |  |  |
|  |  |  |  | 441.7 | 2 | IFLTCTK |  |  |  |
|  |  |  |  | 708.8 | 2 | KFWEEEGIHGGK |  |  |  |
|  |  |  |  | 571.3 | 3 | DCGDIVINDLSLIHK |  |  |  |
|  | 0.18 | 36^▼^ |  | 652.8 | 2 | SFCYPSMIQR | Acont_LAAO-1_e495 | LAAO |  |
|  |  |  |  | 757.8 | 2 | ETDYEEFLEIAR |  |  |  |
|  |  |  |  | 524.6 | 3 | DPGLLEYPVKPSEK |  |  |  |
|  |  |  |  | 643.8 | 2 | SAGQLYEESFR |  |  |  |
|  |  |  |  | 562.3 | 2 | HDDIFGYEK |  |  |  |
|  |  |  |  | 555.8 | 2 | FDEIVGGMDK |  |  |  |
|  |  |  |  | 583.4 | 2 | IKFEPPLPPK |  |  |  |
|  |  |  |  | 462.8 | 2 | FEPPLPPK |  |  |  |
|  |  |  |  | 441.7 | 2 | IFLTCTK |  |  |  |
|  | 0.04 | 19^▼^ |  | 472.9 | 3 | KFWEEEGIHGGK | Acont_LAAO-1_e495 | LAAO |  |
|  |  |  |  | 532.7 | 2 | NPLEECFR |  |  |  |
|  |  |  |  | 757.8 | 2 | ETDYEEFLEIAR |  |  |  |
|  |  |  |  | 524.6 | 3 | DPGLLEYPVKPSEK |  |  |  |
| **28** | 1.72 | 60^▼^ |  | 643.8 | 2 | SAGQLYEESFR | Acont_LAAO-1_e495 | LAAO |  |
|  |  |  |  | 532.7 | 2 | NPLEECFR |  |  |  |
|  |  |  |  | 757.8 | 2 | ETDYEEFLEIAR |  |  |  |
|  |  |  |  | 797.9 | 2 | DKEDWYANLGPMR |  |  |  |
|  |  |  |  | 749.1 | 3 | VSEVMoxKDPGLLEYPVKPSEK |  |  |  |
|  |  |  |  | 524.6 | 3 | DPGLLEYPVKPSEK |  |  |  |
|  |  |  |  | 643.8 | 2 | SAGQLYEESFR |  |  |  |
|  |  |  |  | 562.3 | 2 | HDDIFGYEK |  |  |  |
|  |  |  |  | 557.8 | 2 | VIEIQQNDR |  |  |  |
|  |  |  |  | 583.4 | 2 | IKFEPPLPPK |  |  |  |
|  |  |  |  | 462.8 | 2 | FEPPLPPK |  |  |  |
|  |  |  |  | 441.7 | 2 | IFLTCTK |  |  |  |
|  |  |  |  | 430.2 | 3 | FWEEEGIHGGK |  |  |  |
|  | 0.17 | 45^▼^ |  | 856.5 | 2 | DCGDIVINDLSLIHK | Acont_LAAO-1_e495 | LAAO |  |
|  |  |  |  | 757.8 | 2 | ETDYEEFLEIAR |  |  |  |
|  |  |  |  | 524.6 | 3 | DPGLLEYPVKPSEK |  |  |  |
|  |  |  |  | 562.3 | 2 | HDDIFGYEK | Acont_LAAO-1_e495 | LAAO |  |
|  |  |  |  | 532.7 | 2 | NPLEECFR |  |  |  |
|  |  |  |  | 757.8 | 2 | ETDYEEFLEIAR |  |  |  |
|  |  |  |  | 532.3 | 3 | DKEDWYANLGPMR |  |  |  |
|  |  |  |  | 524.6 | 3 | DPGLLEYPVKPSEK |  |  |  |
|  |  |  |  | 643.8 | 2 | SAGQLYEESFR |  |  |  |
|  | 0.02 | 19^▼^ |  | 472.2 | 3 | SAGQLYEESFRK | Acont_LAAO-1_e495 | LAAO |  |
|  |  |  |  | 532.7 | 2 | NPLEECFR |  |  |  |
|  |  |  |  | 757.8 | 2 | ETDYEEFLEIAR |  |  |  |
|  |  |  |  | 574.9 | 3 | KDKEDWYANLGPMR |  |  |  |
|  |  |  |  | 532.3 | 3 | DKEDWYANLGPMR |  |  |  |
|  |  |  |  | 524.6 | 3 | DPGLLEYPVKPSEK |  |  |  |
|  |  |  |  | 643.8 | 2 | SAGQLYEESFR |  |  |  |
| **29** | 4.30 | 60^▼^ |  | 472.2 | 3 | SAGQLYEESFRK | Acont_LAAO-1_e495 | LAAO |  |
|  |  |  |  | 757.8 | 2 | ETDYEEFLEIAR |  |  |  |
|  |  |  |  | 574.9 | 3 | KDKEDWYANLGPMR |  |  |  |
|  |  |  |  | 676.3 | 2 | EDWYANLGPMR |  |  |  |
|  |  |  |  | 749.1 | 3 | VSEVMKDPGLLEYPVKPSEK |  |  |  |
|  |  |  |  | 524.6 | 3 | DPGLLEYPVKPSEK |  |  |  |
|  |  |  |  | 643.8 | 2 | SAGQLYEESFR |  |  |  |
|  |  |  |  | 562.3 | 2 | HDDIFGYEK |  |  |  |
|  |  |  |  | 643.8 | 2 | SAGQLYEESFR |  |  |  |
|  |  |  |  | 557.8 | 2 | VIEIQQNDR |  |  |  |
|  |  |  |  | 583.4 | 2 | IKFEPPLPPK |  |  |  |
|  |  |  |  | 441.7 | 2 | IFLTCTK |  |  |  |
|  |  |  |  | 472.9 | 3 | KFWEEEGIHGGK |  |  |  |
|  |  |  |  | 430.2 | 3 | FWEEEGIHGGK |  |  |  |
|  |  |  |  | 571.3 | 3 | DCGDIVINDLSLIHK |  |  |  |
|  | 0.38 | 50^▼^ |  | 829.4 | 2 | GIGDDANFFQALDFK | Acont_LAAO-1_e495 | LAAO |  |
|  |  |  |  | 532.7 | 2 | NPLEECFR |  |  |  |
|  |  |  |  | 757.8 | 2 | ETDYEEFLEIAR |  |  |  |
|  |  |  |  | 524.6 | 3 | DPGLLEYPVKPSEK |  |  |  |
|  |  |  |  | 643.8 | 2 | SAGQLYEESFR |  |  |  |
|  |  |  |  | 562.3 | 2 | HDDIFGYEK |  |  |  |
|  |  |  |  | 557.8 | 2 | VIEIQQNDR |  |  |  |
|  |  |  |  | 441.7 | 2 | IFLTCTK |  |  |  |
|  | 0.05 | 45^▼^ |  | 571.3 | 3 | DCGDIVINDLSLIHK | Acont_LAAO-1_e495 | LAAO |  |
|  |  |  |  | 524.6 | 3 | DPGLLEYPVKPSEK |  |  |  |
|  |  |  |  | 643.8 | 2 | SAGQLYEESFR |  |  |  |
|  |  |  |  | 562.3 | 2 | HDDIFGYEK |  |  |  |
|  |  |  |  | 557.8 | 2 | VIEIQQNDR |  |  |  |
|  |  |  |  | 583.4 | 2 | IKFEPPLPPK |  |  |  |
|  |  |  |  | 462.8 | 2 | FEPPLPPK |  |  |  |
|  |  |  |  | 441.7 | 2 | IFLTCTK |  |  |  |
|  |  |  |  | 472.9 | 3 | KFWEEEGIHGGK |  |  |  |
|  | 0.05 | 19^▼^ |  | 571.3 | 3 | DCGDIVINDLSLIHK | Acont_LAAO-1_e495 | LAAO |  |
|  |  |  |  | 532.7 | 2 | NPLEECFR |  |  |  |
|  |  |  |  | 757.8 | 2 | ETDYEEFLEIAR |  |  |  |
|  |  |  |  | 532.3 | 3 | DKEDWYANLGPMR |  |  |  |
|  |  |  |  | 524.6 | 3 | DPGLLEYPVKPSEK |  |  |  |
| **32** | 0.75 | 60^▼^ |  | 643.8 | 2 | SAGQLYEESFR | Acont_LAAO-1_e495 | LAAO |  |
|  |  |  |  | 532.7 | 2 | NPLEECFR |  |  |  |
|  |  |  |  | 757.8 | 2 | ETDYEEFLEIAR |  |  |  |
|  |  |  |  | 643.8 | 2 | SAGQLYEESFR |  |  |  |
|  |  |  |  | 557.8 | 2 | VIEIQQNDR |  |  |  |
|  |  |  |  | 430.2 | 3 | FWEEEGIHGGK |  |  |  |
|  | 0.36 | 50^▼^ |  | 571.3 | 3 | DCGDIVINDLSLIHK | Acont_LAAO-1_e495 | SVMP |  |
|  |  |  |  | 502.3 | 2 | NMPQCILK |  |  |  |
|  |  |  |  | 529.8 | 2 | IACEPQNVK |  |  |  |
|  | 0.13 | 50^▼^ |  | 684.8 | 2 | LYCFPNSPENK | Acont_LAAO-1_e495 | LAAO |  |
|  |  |  |  | 532.7 | 2 | NPLEECFR |  |  |  |
|  |  |  |  | 757.8 | 2 | ETDYEEFLEIAR |  |  |  |
|  |  |  |  | 562.3 | 2 | HDDIFGYEK |  |  |  |
| **33** | 1.68 | 60^▼^ |  | 557.8 | 2 | VIEIQQNDR | Acont_LAAO-1_e495 | LAAO |  |
|  |  |  |  | 532.7 | 2 | NPLEECFR |  |  |  |
|  |  |  |  | 757.8 | 2 | ETDYEEFLEIAR |  |  |  |
|  |  |  |  | 524.6 | 3 | DPGLLEYPVKPSEK |  |  |  |
|  |  |  |  | 562.3 | 2 | HDDIFGYEK |  |  |  |
| **35** | 0.06 | 66^▼^ |  | 571.3 | 3 | DCGDIVINDLSLIHK | Acont_LAAO-1_e495 | LAAO |  |
|  |  |  |  | 643.8 | 2 | SAGQLYEESFR |  |  |  |
|  |  |  |  | 557.8 | 2 | VIEIQQNDR |  |  |  |
|  | 0.09 | 66^▼^ |  | 438.7 | 2 | STTDLPSR |  |  |  |
|  |  |  |  | 534.3 | 2 | YNGDSDKIR | Acont_SVMPI-3a_e522(335,279,me383) | SVMP | * |
|  |  |  |  | 755.7 | 3 | DLSTVTSVSHDTLASFENWR | Acont_SVMPI-3a_e522 | SVMP |  |
|  |  |  |  | 430.3 | 2 | KTDLLNR | Acont_SVMPI-3a_e522(1M61) | SVMP | * |
|  |  |  |  | 723.7 | 3 | SHDNAQLLTAIVFDEGIIGR | Acont_SVMPI-3a_e522 | SVMP |  |
|  | 0.70 | 50^▼^ |  | 626.8 | 2 | MVNTINEIYR | Acont_SVMPI-3a_e522 | SVMP |  |
|  |  |  |  | 657.4 | 2 | YVELVIVADHR | Acont_SVMPI-3a_e522(me383,44,279..) | SVMP | * |
|  |  |  |  | 534.3 | 2 | YNGDSDKIR | Acont_SVMPI-3a_e522(335,279,me383) | SVMP | * |
|  |  |  |  | 755.7 | 3 | DLSTVTSVSHDTLASFENWR | Acont_SVMPI-3a_e522 | SVMP |  |
|  |  |  |  | 430.3 | 2 | KTDLLNR | Acont_SVMPI-3a_e522(1M61) | SVMP | * |
|  |  |  |  | 657.4 | 2 | YVELVIVADHR | Acont_SVMPI-3a_e522(me383,44,279..) | SVMP | * |
|  | 0.07 | 50^▼^ |  | 534.3 | 2 | YNGDSDKIR | Acont_SVMPI-3a_e522(335,279,me383) | SVMP | * |
|  |  |  |  | 532.7 | 2 | NPLEECFR | Acont_LAAO-1_e495 | LAAO |  |
|  | 0.66 | 36^▼^ |  | 757.8 | 2 | ETDYEEFLEIAR |  |  |  |
|  |  |  |  | 657.4 | 2 | YVELVIVADHR | Acont_SVMPI-3a_e522(me383,44,279..) | SVMP | * |
|  |  |  |  | 534.8 | 2 | YNGDSDKIR |  |  |  |
|  |  |  |  | 755.7 | 3 | DLSTVTSVSHDTLASFENWR | Acont_SVMPI-3a_e522 | SVMP |  |
|  | 0.10 | 36^▼^ |  | 430.3 | 2 | KTDLLNR | Acont_SVMPI-3a_e522(1M61) | SVMP | * |
|  |  |  |  | 643.8 | 2 | SAGQLYEESFR | Acont_LAAO-1_e495 | LAAO |  |
|  |  |  |  | 557.8 | 2 | VIEIQQNDR |  |  |  |
|  | 0.23 | 31^▼^ |  | 571.3 | 3 | DCGDIVINDLSLIHK |  |  |  |
|  |  |  |  | 534.3 | 2 | YNGDSDKIR | Acont_SVMPI-3a_e522(335,279,me383) | SVMP | * |
|  |  |  |  | 430.3 | 2 | KTDLLNR | Acont_SVMPI-3a_e522(1M61) | SVMP | * |
|  |  |  |  | 723.7 | 3 | SHDNAQLLTAIVFDEGIIGR | Acont_SVMPI-3a_e522 | SVMP |  |
|  | 0.01 | 31^▼^ |  | 626.8 | 2 | MVNTINEIYR |  |  |  |
|  | 0.07 | 31^▼^ |  | 486.3 | 2 | IYLGVHNR | Acont_SVSP-11a_e515(339,369) | SVMP |  |
|  |  |  |  | 557.8 | 2 | VIEIQQNDR | Acont_LAAO-1_e495 | LAAO |  |
|  |  |  |  | 438.7 | 2 | STTDLPSR |  |  |  |
|  | 0.15 | 28^▼^ |  | 571.3 | 3 | DCGDIVINDLSLIHK |  |  |  |
|  |  |  |  | 534.3 | 2 | YNGDSDKIR | Acont_SVMPI-3a_e522(335,279,me383) | SVMP | * |
|  |  |  |  | 430.3 | 2 | KTDLLNR | Acont_SVMPI-3a_e522(1M61) | SVMP | * |
|  |  |  |  | 723.7 | 3 | SHDNAQLLTAIVFDEGIIGR | Acont_SVMPI-3a_e522 | SVMP |  |
|  |  |  |  | 482.7 | 2 | GDIGIAYGAK | Acont_SVMPI-2a_e44 | SVMP |  |
|  |  |  |  | 457.7 | 2 | DYQTFLK |  |  |  |
|  | 0.01 | 28^▼^ |  | 626.8 | 2 | MVNTINEIYR | Acont_SVMPI-3a_e522 | SVMP |  |
|  | 9.16 | 25^▼^ |  | 657.4 | 2 | YVELVIVADHR | Acont_SVMPI-3a_e522 | SVMP |  |
|  |  |  |  | 534.3 | 2 | YNGDSDKIR |  |  |  |
|  |  |  |  | 755.5 | 3 | DLSTVTSVSHDTLASFENWR |  |  |  |
|  |  |  |  | 1133.1 | 2 | DLSTVTSVSHDTLASFENWR |  |  |  |
|  |  |  |  | 430.3 | 2 | KTDLLNR |  |  |  |
|  |  |  |  | 1085.1 | 2 | SHDNAQLLTAIVFDEGIIGR |  |  |  |
|  |  |  |  | 709.9 | 2 | VSLTDLEVWSNR | Acont_SVMPII-5a_e416 | SVMP |  |
|  |  |  |  | 492.7 | 2 | LFSDCSKK | Acont_SVMPI-1_me383(44,522,575,335..) | SVMP | * |
|  | 0.15 | 21^▼^ |  | 626.8 | 2 | MVNTINEIYR | Acont_SVMPI-3a_e522 | SVMP |  |
|  |  |  |  | 534.3 | 2 | YNGDSDKIR |  |  |  |
|  |  |  |  | 755.5 | 3 | DLSTVTSVSHDTLASFENWR |  |  |  |
|  |  |  |  | 430.3 | 2 | KTDLLNR |  |  |  |
|  |  |  |  | 723.7 | 3 | SHDNAQLLTAIVFDEGIIGR |  |  |  |
|  |  |  |  | 492.7 | 2 | LFSDCSKK |  |  |  |
|  |  |  |  | 534.3 | 2 | YNGDSDKIR |  |  |  |
|  | 0.04 | 19^▼^ |  | 626.8 | 2 | MVNTINEIYR | Acont_SVMPI-3a_e522 | SVMP |  |
|  |  |  |  | 757.8 | 2 | ETDYEEFLEIAR | Acont_LAAO-1_e495 | LAAO |  |
|  |  |  |  | 524.6 | 3 | DPGLLEYPVKPSEK |  |  |  |
|  | 0.12 | 19^▼^ |  | 643.8 | 2 | SAGQLYEESFR |  |  |  |
|  |  |  |  | 534.3 | 2 | YNGDSDKIR |  |  |  |
|  | 0.15 | 18^▼^ |  | 430.3 | 2 | KTDLLNR | Acont_SVMPI-3a_e522 | SVMP |  |
|  |  |  |  | 534.3 | 2 | YNGDSDKIR |  |  |  |
|  |  |  |  | 430.3 | 2 | KTDLLNR |  |  |  |
|  |  |  |  | 723.7 | 3 | SHDNAQLLTAIVFDEGIIGR |  |  |  |
|  |  |  |  | 438.6 | 3 | YVELVIVADHR |  |  |  |
|  | 0.44 | 14^▼^ |  | 755.5 | 3 | DLSTVTSVSHDTLASFENWR | Acont_SVMPI-3a_e522 | SVMP |  |
|  |  |  |  | 657.4 | 2 | YVELVIVADHR |  |  |  |
|  |  |  |  | 534.3 | 2 | YNGDSDKIR |  |  |  |
|  |  |  |  | 430.3 | 2 | KTDLLNR |  |  |  |
|  |  |  |  | 723.7 | 3 | SHDNAQLLTAIVFDEGIIGR |  |  |  |
|  | 0.02 | 14^▼^ |  | 595.3 | 2 | AIVFDEGIIGR | Acont_SVMPI-3a_e522 | SVMP |  |
|  | 1.53 | 8^▼^ |  | 532.7 | 2 | NPLEECFR | Acont_LAAO-1_e495 | LAAO |  |
|  |  |  |  | 657.4 | 2 | YVELVIVADHR | Acont_SVMPI-3a_e522 | SVMP |  |
|  |  |  |  | 534.3 | 2 | YNGDSDKIR |  |  |  |
|  |  |  |  | 605.3 | 3 | YNGDSDKIRQWIYR |  |  |  |
|  |  |  |  | 1133.1 | 2 | DLSTVTSVSHDTLASFENWR |  |  |  |
|  |  |  |  | 1085.1 | 2 | SHDNAQLLTAIVFDEGIIGR |  |  |  |
|  |  |  |  | 438.3 | 3 | YVELVIVADHR |  |  |  |
|  |  |  |  | 755.7 | 3 | DLSTVTSVSHDTLASFENWR |  |  |  |
|  |  |  |  | 595.3 | 2 | AIVFDEGIIGR |  |  |  |
|  | 1.53 | 6^▼^ |  | 626.8 | 2 | MVNTINEIYR | Acont_SVMPI-3a_e522 | SVMP |  |
|  |  |  |  | 657.4 | 2 | YVELVIVADHR |  |  |  |
|  |  |  |  | 755.7 | 3 | DLSTVTSVSHDTLASFENWR |  |  |  |
|  |  |  |  | 430.3 | 2 | KTDLLNR |  |  |  |
| **36** | 0.32 | 50^▼^ |  | 1085.1 | 2 | SHDNAQLLTAIVFDEGIIGR | Acont_SVMPI-3a_e522 | SVMP |  |
|  |  |  |  | 757.8 | 2 | ETDYEEFLEIAR | Acont_LAAO-1_e495 | LAAO |  |
|  |  |  |  | 643.8 | 2 | SAGQLYEESFR |  |  |  |
|  |  |  |  | 562.3 | 2 | HDDIFGYEK |  |  |  |
|  |  |  |  | 555.8 | 2 | FDEIVGGMDK |  |  |  |
|  |  |  |  | 557.8 | 2 | VIEIQQNDR |  |  |  |
|  |  |  |  | 583.4 | 2 | IKFEPPLPPK |  |  |  |
|  | 0.03 | 45^▼^ |  | 441.7 | 2 | IFLTCTK | Acont_LAAO-1_e495 | LAAO |  |
|  |  |  |  | 562.3 | 2 | HDDIFGYEK |  |  |  |
|  |  |  |  | 557.8 | 2 | VIEIQQNDR |  |  |  |
|  | 0.00 | 45^▼^ |  | 438.7 | 2 | STTDLPSR |  |  |  |
|  |  |  |  | 438.6 | 3 | YVELVIVADHR | Acont_SVMPI-3a_e522(me383,44,279..) | SVMP | * |
|  |  |  |  | 482.7 | 2 | GDIGIAYGAK | Acont_SVMPI-2a_e44 | SVMP |  |
|  |  |  |  | 723.7 | 3 | SHDNAQLLTAIVFDEGIIGR | Acont_SVMPI-3a_e522 | SVMP |  |
|  | 0.03 | 31^▼^ |  | 595.3 | 2 | AIVFDEGIIGR | Acont_SVMPII-2_me463 | SVMP |  |
|  |  |  |  | 438.6 | 3 | YVELVIVADHR | Acont_SVMPI-3a_e522(me383,44,279..) | SVMP | * |
|  |  |  |  | 461.7 | 2 | GVIGSAYGAK | Acont_SVMPI-3a_e522 | SVMP |  |
|  | 0.00 | 31^▼^ |  | 723.7 | 3 | SHDNAQLLTAIVFDEGIIGR | Acont_SVMPI-3a_e522 | SVMP |  |
|  |  |  |  | 562.3 | 2 | HDDIFGYEK | Acont_LAAO-1_e495 | LAAO |  |
|  | 2.54 | 28^▼^ |  | 557.8 | 2 | VIEIQQNDR |  |  |  |
|  |  |  |  | 438.6 | 3 | YVELVIVADHR | Acont_SVMPI-3a_e522(me383,44,279..) | SVMP | * |
|  |  |  |  | 482.7 | 2 | GDIGIAYGAK | Acont_SVMPI-2a_e44 | SVMP |  |
|  |  |  |  | 608.8 | 2 | FSVGIVQDHSK |  |  |  |
|  | 0.16 | 25^▼^ |  | 521.8 | 2 | KDYQTFLK |  |  |  |
|  |  |  |  | 534.3 | 2 | YNGDSDKIR | Acont_SVMPI-1_me383(522,335,279) | SVMP | * |
|  |  |  |  | 430.3 | 2 | KTDLLNR |  |  |  |
|  |  |  |  | 723.7 | 3 | SHDNAQLLTAIVFDEGIIGR | Acont_SVMPI-3a_e522 | SVMP |  |
|  |  |  |  | 492.7 | 2 | LFSDCSKK | Acont_SVMPI-1_me383(522,335,279) | SVMP | * |
|  |  |  |  | 482.7 | 2 | GDIGIAYGAK | Acont_SVMPI-2a_e44 | SVMP |  |
|  | 0.10 | 6^▼^ |  | 626.8 | 2 | MVNTINEIYR | Acont_SVMPI-3a_e522(575) | SVMP | * |
|  |  |  |  | 406.2 | 3 | FSVGIVQDHSK | Acont_SVMPI-2a_e44 | SVMP |  |
|  |  |  |  | 521.8 | 2 | KDYQTFLK | Acont_SVMPI-3a_e522 | SVMP |  |
|  |  |  |  | 430.3 | 2 | KTDLLNR |  |  |  |
| **37** | 0.39 | 50^▼^ |  | 723.7 | 3 | SHDNAQLLTAIVFDEGIIGR | Acont_SVMPI-3a_e522 | SVMP |  |
|  |  |  |  | 757.8 | 2 | ETDYEEFLEIAR | Acont_LAAO-1_e495 | LAAO |  |
|  |  |  |  | 643.8 | 2 | SAGQLYEESFR |  |  |  |
|  |  |  |  | 562.3 | 2 | HDDIFGYEK |  |  |  |
|  |  |  |  | 555.8 | 2 | FDEIVGGMDK |  |  |  |
|  |  |  |  | 557.8 | 2 | VIEIQQNDR |  |  |  |
|  | 3.09 | 25^▼^ |  | 441.7 | 2 | IFLTCTK |  |  |  |
|  |  |  |  | 626.8 | 2 | MVNTINEIYR | Acont_SVMPI-3a_e522(575) | SVMP | * |
|  |  |  |  | 601.3 | 2 | NVPNEDEQTR | Acont_SVSP-6_98-10M191 | SVMP |  |
|  | 0.04 | 21^▼^ |  | 514.3 | 2 | KDYLTFLK | Acont_SVMPI-4a_e575 | SVMP |  |
|  | 0.04 | 16^▼^ |  | 626.8 | 2 | MVNTINEIYR | Acont_SVMPI-3a_e522(575) | SVMP | * |
|  |  |  |  | 626.8 | 2 | MVNTINEIYR | Acont_SVMPI-3a_e522(575) | SVMP | * |
|  | 0.00 | 16^▼^ |  | 609.3 | 2 | APLAGMoxCDPNR | Acont_SVMPI-4a_e575 | SVMP |  |
|  |  |  |  | 757.8 | 2 | ETDYEEFLEIAR | Acont_LAAO-1_e495 | LAAO |  |
|  | 0.04 | 14^▼^ |  | 643.8 | 2 | SAGQLYEESFR |  |  |  |
|  |  |  |  | 626.8 | 2 | MVNTINEIYR | Acont_SVMPI-3a_e522(575) | SVMP | * |
|  |  |  |  | 601.3 | 2 | NVPNEDEQTR | Acont_SVSP-6_98-10M191 | SVMP |  |
|  | 0.08 | 8^▼^ |  | 609.3 | 2 | APLAGMoxCDPNR | Acont_SVMPI-4a_e575 | SVMP |  |
|  | 0.19 | 6^▼^ |  | 626.8 | 2 | MVNTINEIYR | Acont_SVMPI-3a_e522(575) | SVMP | * |

**Table S2.** Identification by MS/MS of the venom components of *Agkistrodon c. contortrix* specimen M74 (parthenogen) (Fig. 2B). *, ambiguous ID.

| **Spot ID** | **%** | **MW (kDa)** | **ESI-MS (ave)** | **m/z** | **z** | **Peptide sequence** | **Transcriptome match** | **Protein family** | **Observations** |
| --- | --- | --- | --- | --- | --- | --- | --- | --- | --- |
|  |  |  |  |  |  |  |  |  |  |
| **1** | 0.24 |  |  | 532.3 | 2 | TPPAGPDVGPR | Acont_BPP-1a_98 | VAP (BIP) |  |
| **2** | 1.84 |  |  | 532.3 | 2 | TPPAGPDVGPR | Acont_BPP-1a_98 | VAP (BIP) |  |
| **5** | 0.69 |  |  | 444.2 | 1 | ZQW | Acont_BPP-1a_98 | SVMPi |  |
| **6** | 0.12 |  |  | 430.2 | 1 | ZNW | Acont_BPP-1a_98 | SVMPi |  |
| **7** | 0.17 |  | 13010,9/  13450,9 | 598.8 | 2 | NPCCDAATCK | Acont_DIS-2a_e351 | Dimeric Disintegrin | Acostatin alpha (Q805F7) |
|  |  |  |  | 992.4 | 2 | LTPGSQCAEGLCCDQCK |  |  | Mave calc (homodimer ^1^QP..KH^61^): 13016,5 |
|  |  |  |  | 569.2 | 2 | CTGQSGDCPR |  |  |  |
| **8** | 0.31 |  | 13450.1 | 775.4 | 2 | DAPANPCCDAATCK | Acont_SVMPII-5a_e416 | Dimeric Disintegrin | Acostatin beta (BAC55945) |
| **9** | 1.24 | 8^▼^ | 13450.6 | 775.4 | 2 | DAPANPCCDAATCK | Acont_SVMPII-5a_e416 | Dimeric Disintegrin |  |
|  |  |  |  | 942.9 | 2 | GDDLDDYCNGISAGCPR |  |  |  |
|  |  |  |  | 598.8 | 2 | NPCCDAATCK | Acont_DIS-2a_e351 | Dimeric Disintegrin | Acostatin alpha (Q805F7) |
|  |  |  |  | 992.4 | 2 | LTPGSQCAEGLCCDQCK |  |  |  |
| **10** | 0.02 | 8^▼^ |  | 598.8 | 2 | NPCCDAATCK | Acont_DIS-2a_e351 | Dimeric Disintegrin | Acostatin alpha (Q805F7) |
|  |  |  |  | 791.4 | 3 | LTPGSQCAEGLCCDQCKFIK |  |  |  |
| **11** | 0.11 |  | 13369.2 |  |  |  |  |  | Mave calc Acostatin (heterodimer ab): 13369,7 |
|  |  |  |  | 791.4 | 3 | LTPGSQCAEGLCCDQCKFIK | Acont_DIS-2a_e351 | Dimeric Disintegrin | Acostatin alpha (Q805F7) |
|  |  | 8^▼^ |  | 987.4 | 2 | LTTGSQCADGLCCDQCK | Acont_SVMPII-5a_e416 | Dimeric disintegrin | Acostatin beta (BAC55945) |
|  |  |  |  | 942.9 | 2 | GDDLDDYCNGISAGCPR |  |  |  |
| **15** | 0.21 | 31^▼^ |  | 468.3 | 2 | MoxILQETGK | Acont_PLA2-3a_e604 | PLA2 |  |
|  |  |  |  | 707.3 | 3 | NAITSYGFYGCNCGWGHR |  |  |  |
|  |  |  |  | 498.7 | 2 | ENLDTYNK |  |  |  |
|  |  |  |  | 562.8 | 2 | ENLDTYNKK |  |  |  |
|  |  |  |  | 480.3 | 2 | KYNPYFK |  |  |  |
|  |  |  |  | 416.2 | 2 | YNPYFK |  |  |  |
|  | 10.18 | 14^▼^ | 14042.5 | 468.3 | 2 | MoxILQETGK | Acont_PLA2-3a_e604 | PLA2 |  |
|  |  |  |  | 707.3 | 3 | NAITSYGFYGCNCGWGHR |  |  |  |
|  |  |  |  | 498.7 | 2 | ENLDTYNK |  |  |  |
|  |  |  |  | 562.8 | 2 | ENLDTYNKK |  |  |  |
|  | 10.18 | 12^▼^ |  | 460.3 | 2 | MILQETGK | Acont_PLA2-3a_e604 | PLA2 |  |
|  |  |  |  | 401.7 | 2 | AVAICLR |  |  |  |
|  |  |  |  | 498.7 | 2 | ENLDTYNK |  |  |  |
|  |  |  |  | 562.8 | 2 | ENLDTYNKK |  |  |  |
|  |  |  |  | 480.3 | 2 | KYNPYFK |  |  |  |
| **17** | 0.09 | 14^▼^ | 14042.5 | 460.3 | 2 | MILQETGK | Acont_PLA2-3a_e604 | PLA2 |  |
|  |  |  |  | 707.3 | 3 | NAITSYGFYGCNCGWGHR |  |  |  |
|  |  |  |  | 498.7 | 2 | ENLDTYNK |  |  |  |
|  |  |  |  | 562.8 | 2 | ENLDTYNKK |  |  |  |
|  | 0.09 | 12^▼^ |  | 468.3 | 2 | MoxILQETGK | Acont_PLA2-3a_e604 | PLA2 |  |
|  |  |  |  | 707.3 | 3 | NAITSYGFYGCNCGWGHR |  |  |  |
|  |  |  |  | 498.7 | 2 | ENLDTYNK |  |  |  |
| **18** | 0.87 | 31^▼^ |  | 565.3 | 2 | FLVALYTFR | Acont_SVSP-5_e565 | SVSP |  |
|  |  |  |  | 595.3 | 2 | WDKDIMLIR |  |  |  |
|  |  |  |  | 721.7 | 3 | STHIAPLSLPSSPPSLGSVCR |  |  |  |
|  |  |  |  | 559.8 | 2 | TLCAGILEGGK |  |  |  |
|  | 0.87 | 26^▼^ |  | 565.3 | 2 | FLVALYTFR | Acont_SVSP-5_e565 | SVSP |  |
|  |  |  |  | 507.8 | 2 | AAYPQLPVR |  |  |  |
|  |  |  |  | 559.8 | 2 | TLCAGILEGGK |  |  |  |
|  |  |  |  | 500.2 | 3 | VVGGDECNINEHR | Acont_SVSP-3a_e242(9,14,515,339) | SVSP | * |
|  | 0.43 | 14^▼^ |  | 551.3 | 2 | LDIYTYSVK | Acont_PLA2-1a_e343 | PLA2 |  |
|  |  |  |  | 460.3 | 2 | MILQETGK | Acont_PLA2-3a_e604 | PLA2 |  |
|  |  |  |  | 707.3 | 3 | NAITSYGFYGCNCGWGHR |  |  |  |
|  | 0.02 | 14^▼^ |  | 565.3 | 2 | FLVALYTFR | Acont_SVSP-5_e565 | SVSP |  |
|  |  |  |  | 507.8 | 2 | AAYPQLPVR |  |  |  |
|  |  |  |  | 559.8 | 2 | TLCAGILEGGK |  |  |  |
|  | 0.45 | 12^▼^ |  | 507.8 | 2 | AAYPQLPVR | Acont_SVSP-5_e565 | SVSP |  |
| **19** | 0.29 | 31^▼^ |  | 551.3 | 2 | LDIYTYSVK | Acont_PLA2-1a_e343 | PLA2 |  |
|  |  |  |  | 521.5 | 3 | CCFVHDCCYNK |  |  |  |
|  | 0.37 | 31^▼^ |  | 565.3 | 2 | FLVALYTFR | Acont_SVSP-5_e565 | SVSP |  |
|  |  |  |  | 507.8 | 2 | AAYPQLPVR |  |  |  |
|  |  |  |  | 559.8 | 2 | TLCAGILEGGK |  |  |  |
|  |  |  |  | 486.3 | 2 | IYLGVHNR | Acont_SVSP-16a_e369(339,515) | SVSP | * |
|  | 0.65 | 26^▼^ |  | 551.3 | 2 | LDIYTYSVK | Acont_PLA2-1a_e343 | PLA2 |  |
|  |  |  |  | 490.7 | 2 | QICECDR |  |  |  |
|  | 9.25 | 14^▼^ |  | 630.0 | 3 | VTSCNPKLDIYTYSVK | Acont_PLA2-1a_e343 | PLA2 |  |
|  |  |  |  | 551.3 | 2 | LDIYTYSVK |  |  |  |
|  |  |  |  | 404.7 | 2 | AAAICFR |  |  |  |
|  | 0.85 | 12^▼^ |  | 760.4 | 2 | NVGVPQVVPDNPER | Acont_Vespryn-1_e296 | Ohanin-like_protein |  |
|  |  |  |  | 763.4 | 2 | FDSSPCVLGSPGFR |  |  |  |
|  |  |  |  | 465.8 | 2 | EWAVGLAGK |  |  |  |
|  |  |  |  | 605.9 | 2 | KGGLLLVPEER |  |  |  |
|  |  |  |  | 541.8 | 2 | GGLLLVPEER |  |  |  |
|  | 1.62 | 12^▼^ |  | 551.3 | 2 | LDIYTYSVK | Acont_PLA2-1a_e343 | PLA2 |  |
|  |  |  |  | 404.7 | 2 | AAAICFR |  |  |  |
| **20** | 0.10 | 31^▼^ |  | 544.3 | 2 | VPNEDEQTR | Acont_SVSP-15a_e914 | SVSP |  |
|  |  |  |  | 727.4 | 2 | AAYPEYDLPATSR |  |  |  |
|  |  |  |  | 559.8 | 2 | TLCAGILEGGK |  |  |  |
|  | 0.02 | 31^▼^ |  | 551.3 | 2 | LDIYTYSVK | Acont_PLA2-1a_e343 | PLA2 |  |
|  |  |  |  | 490.7 | 2 | QICECDR |  |  |  |
|  | 1.93 | 28^▼^ |  | 459.2 | 2 | NNIKWDK | Acont_SVSP-11a_e515(339) | SVSP | * |
|  |  |  |  | 553.6 | 3 | NNIKWDKDIMLIR |  |  |  |
|  |  |  |  | 595.3 | 2 | WDKDIMLIR |  |  |  |
|  |  |  |  | 753.4 | 3 | NSAHIAPHSLPSNAPSVGSVCR |  |  |  |
|  |  |  |  | 766.4 | 2 | GLAATTLCAGILEGGK | Acont_SVSP-16a_e369(982) | SVSP | * |
|  | 0.08 | 26^▼^ |  | 553.6 | 3 | NNIKWDKDIMLIR | Acont_SVSP-11a_e515(339) | SVSP | * |
|  |  |  |  | 595.3 | 2 | WDKDIMLIR |  |  |  |
|  | 0.04 | 26^▼^ |  | 551.3 | 2 | LDIYTYSVK | Acont_PLA2-1a_e343 | PLA2 |  |
|  | 0.12 | 21^▼^ |  | 553.6 | 3 | NNIKWDKDIMLIR | Acont_SVSP-11a_e565 | SVSP |  |
|  |  |  |  | 565.3 | 2 | FLVALYTFR |  |  |  |
|  |  |  |  | 766.4 | 2 | GLAATTLCAGILEGGK | Acont_SVSP-16a_e369(982) | SVSP | * |
|  | 0.12 | 14^▼^ |  | 1023.1 | 3 | SGVLWYSAYGCYCGWGGQGRPQDATDR | Acont_PLA2-1a_e343 | PLA2 |  |
|  |  |  |  | 521.5 | 3 | CCFVHDCCYNK |  |  |  |
|  |  |  |  | 603.8 | 2 | DVVCGGTNPCK |  |  |  |
|  |  |  |  | 490.7 | 2 | QICECDR |  |  |  |
| **20a** | 0.86 | 31^▼^ |  | 486.3 | 2 | IYLGVHNR | Acont_SVSP-11a_e515(339,369) | SVSP | * |
|  |  |  |  | 766.4 | 2 | GLAATTLCAGILEGGK | Acont_SVSP-16a_e369 | SVSP |  |
|  |  |  |  | 654.3 | 2 | NFQMLFGVHSK | Acont_SVSP-2a_e957 | SVSP |  |
|  |  |  |  | 596.8 | 2 | IMGWGTTTPTK |  |  |  |
|  |  |  |  | 751.4 | 3 | AAYPELLAESSTLCAGTQQGGK |  |  |  |
|  | 0.86 | 29^▼^ |  | 559.3 | 2 | ILNEDEQTR | Acont_SVSP-2a_e957 | SVSP |  |
|  |  |  |  | 403.7 | 2 | FICPNR |  |  |  |
|  |  |  |  | 596.8 | 2 | IMGWGTTTPTK |  |  |  |
|  |  |  |  | 766.4 | 2 | GLAATTLCAGILEGGK | Acont_SVSP-16a_e369(982) | SVSP | * |
|  | 0.37 | 21^▼^ |  | 766.4 | 2 | GLAATTLCAGILEGGK | Acont_SVSP-16a_e369(982) | SVSP | * |
|  |  |  |  | 559.8 | 2 | TLCAGILEGGK |  | SVSP | * |
|  | 0.20 | 14^▼^ |  | 551.3 | 2 | LDIYTYSVK | Acont_PLA2-1a_e343 | PLA2 |  |
|  | 0.17 | 14^▼^ |  | 486.3 | 2 | IYLGVHNR | Acont_SVSP-11a_e515(339,369) | SVSP | * |
| **21** | 0.14 | 36^▼^ |  | 405.9 | 3 | KVPNEDEQTR | Acont_SVSP-15a_e914 | SVSP |  |
|  |  |  |  | 544.3 | 2 | VPNEDEQTR |  |  |  |
|  |  |  |  | 727.4 | 2 | AAYPEYDLPATSR |  |  |  |
|  |  |  |  | 559.8 | 2 | TLCAGILEGGK |  |  |  |
|  |  |  |  | 550.9 | 3 | TLCAGILEGGKDTCR |  |  |  |
|  |  |  |  | 571.8 | 2 | FLVALYNFR | Acont_SVSP-14a_e824 | SVSP |  |
|  |  |  |  | 544.3 | 2 | VPNEDEQTR |  |  |  |
|  |  |  |  | 750.7 | 3 | NSAHIAPLSLPSNSPSVGSVCR | Acont_SVSP-18a_e982 | SVSP |  |
|  | 0.14 | 31^▼^ |  | 750.7 | 3 | NSAHIAPLSLPSNSPSVGSVCR | Acont_SVSP-18a_e982 | SVSP |  |
|  |  |  |  | 727.4 | 2 | AAYPEYDLPATSR | Acont_SVSP-15a_e914 | SVSP |  |
|  | 0.14 | 29^▼^ |  | 766.4 | 2 | GLAATTLCAGILEGGK | Acont_SVSP-16a_e369(982) | SVSP | * |
|  |  |  |  | 559.8 | 2 | TLCAGILEGGK |  |  |  |
|  |  |  |  | 403.7 | 2 | FICPNR | Acont_SVSP-2a_e957 | SVSP |  |
|  |  |  |  | 596.8 | 2 | IMGWGTTTPTK |  |  |  |
|  | 0.14 | 16^▼^ |  | 502.5 | 3 | CCFVHDCCYGK | Acont_PLA2-4_e312 | PLA2 |  |
|  |  |  |  | 491.2 | 2 | EICECDR |  |  |  |
|  | 0.14 | 14^▼^ |  | 404.7 | 2 | AAAICFR | Acont_PLA2-1a_e343(312,1f2202) | PLA2 | * |
| **22** | 0.36 | 46^▼^ |  | 491.2 | 2 | EICECDR | Acont_PLA2-4_e312 | PLA2 |  |
|  |  |  |  | 404.7 | 2 | AAAICFR |  |  |  |
|  |  |  |  | 750.7 | 3 | NSAHIAPLSLPSNSPSVGSVCR | Acont_SVSP-18a_e982 | SVSP |  |
|  | 0.72 | 34^▼^ |  | 487.2 | 2 | YFCLNTR | Acont_SVSP-18a_e982 | SVSP |  |
|  |  |  |  | 615.8 | 2 | EKYFCLNTR |  |  |  |
|  |  |  |  | 830.7 | 3 | SLCCGTLINQEWVLSAAHCDR | Acont_SVSP-16a_e369 | SVSP |  |
|  |  |  |  | 486.3 | 2 | IYLGVHNR |  |  |  |
|  |  |  |  | 681.4 | 2 | SRTLCAGILEGGK | Acont_SVSP-14a_e824(914,10M191) | SVSP | * |
|  |  |  |  | 559.8 | 2 | TLCAGILEGGK |  |  |  |
|  |  |  |  | 544.3 | 2 | VPNEDEQTR | Acont_SVSP-14a_e824(914,10M191) | SVSP | * |
|  | 0.36 | 31^▼^ |  | 486.3 | 2 | IYLGVHNR | Acont_SVSP-11a_e515(339) | SVSP | * |
|  |  |  |  | 753.4 | 3 | NSAHIAPHSLPSNAPSVGSVCR |  |  |  |
|  |  |  |  | 400.9 | 3 | AAYPELPVKGR | Acont_SVSP-8_e14 | SVSP |  |
|  |  |  |  | 727.4 | 2 | AAYPEYDLPATSR | Acont_SVSP-15a_e914 | SVSP |  |
|  | 5.04 | 14^▼^ | 13769.0/ 13753.0 | 491.2 | 2 | EICECDR | Acont_PLA2-4_e312 | PLA2 |  |
|  |  |  |  | 404.7 | 2 | AAAICFR |  |  |  |
|  |  |  |  | 556.7 | 2 | DNKDTYDNK |  |  |  |
|  |  |  |  | 539.9 | 3 | DNKDTYDNKYWR |  |  |  |
|  | 0.36 | 6^▼^ |  | 502.5 | 3 | CCFVHDCCYGK | Acont_PLA2-4_e312(1f2202) | PLA2 | * |
|  | 0.36 | 5^▼^ |  | 502.5 | 3 | CCFVHDCCYGK | Acont_PLA2-4_e312(1f2202) | PLA2 | * |
| **23** | 0.67 | 46^▼^ |  | 773.1 | 3 | NSEHIAPLSLPSSPPIVGSVCR | Acont_SVSP-13a_e537 | SVSP |  |
|  | 0.34 | 33^▼^ |  | 803.4 | 2 | ILCAGVLEGGIDTCK | Acont_SVSP-13a_e537 | SVSP |  |
|  |  |  |  | 587.9 | 3 | ILCAGVLEGGIDTCKR |  |  |  |
|  |  |  |  | 549.3 | 2 | FLALVYTDR | Acont_SVSP-8_e14 | SVSP |  |
|  |  |  |  | 559.8 | 2 | TLCAGILEGGK | Acont_SVSP-6_98-10M191 | SVSP |  |
|  |  |  |  | 466.7 | 2 | YFCLSSR | Acont_SVSP-10_98-10M279 | SVSP |  |
|  |  |  |  | 741.7 | 3 | NSAHIAPLSLPSSSPSVGSVCR |  |  |  |
|  |  |  |  | 519.3 | 2 | AAHGGLPATSR |  |  |  |
|  |  |  |  | 797.4 | 2 | TLCAGILEGGIDSCK |  |  |  |
|  |  |  |  | 487.2 | 2 | YFCLNTR | Acont_SVSP-18a_e982 | SVSP |  |
|  |  |  |  | 750.7 | 3 | NSAHIAPLSLPSNSPSVGSVCR |  |  |  |
|  |  |  |  | 500.2 | 3 | VVGGDECNINEHR | Acont_SVSP-3a_e242(9,14,515,339) | SVSP | * |
|  |  |  |  | 558.3 | 2 | VLNEDEQIR | Acont_SVSP-3a_e242 | SVSP |  |
|  |  |  |  | 595.8 | 2 | IMGWGTISPTK | Acont_SVSP-8_e14(857) | SVSP | * |
|  |  |  |  | 486.3 | 2 | IYLGVHNR | Acont_SVSP-16a_e369(515,339) | SVSP | * |
|  |  |  |  | 773.1 | 3 | NSEHIAPLSLPSSPPIVGSVCR | Acont_SVSP-13a_e537 | SVSP |  |
|  | 2.53 | 31^▼^ |  | 486.3 | 2 | IYLGVHNR | Acont_SVSP-16a_e369(515,339) | SVSP | * |
|  |  |  |  | 750.9 | 2 | LPATTLCAGILEGGK | Acont_SVSP-17a_e339 | SVSP |  |
|  |  |  |  | 403.7 | 2 | KPGLYTK |  |  |  |
|  |  |  |  | 549.3 | 2 | FLALVYTDR | Acont_SVSP-8_e14 | SVSP |  |
|  |  |  |  | 833.1 | 3 | VILPDVPHCANINLLNYSECR |  |  |  |
|  |  |  |  | 494.3 | 2 | AAYPELPVK |  |  |  |
|  |  |  |  | 486.3 | 2 | IYLGVHNR | Acont_SVSP-16a_e369(515,339) | SVSP | * |
|  |  |  |  | 403.7 | 2 | FICPNR | >Acont_SVSP-7_e420(957) | SVSP | * |
|  |  |  |  | 500.2 | 3 | VVGGDECNINEHR | Acont_SVSP-3a_e242(9,14,515,339) | SVSP | * |
|  |  |  |  | 559.8 | 2 | TLCAGILEGGK |  | SVSP | * |
|  | 2.53 | 29^▼^ |  | 549.3 | 2 | FLALVYTDR | Acont_SVSP-8_e14 | SVSP |  |
|  |  |  |  | 595.8 | 2 | IMGWGTISPTK |  |  |  |
|  |  |  |  | 833.1 | 3 | VILPDVPHCANINLLNYSECR |  |  |  |
|  |  |  |  | 750.9 | 2 | LPATTLCAGILEGGK | Acont_SVSP-17a_e339 | SVSP |  |
|  | 0.34 | 26^▼^ |  | 559.8 | 2 | TLCAGILEGGK |  | SVSP | * |
|  |  |  |  | 401.2 | 3 | LLDKDIMLIK | Acont_SVSP-4_e9 | SVSP |  |
|  |  |  |  | 741.7 | 3 | NSAHIAPLSLPSSSPSVGSVCR | Acont_SVSP-10_98-10M279 | SVSP |  |
|  | 0.30 | 14^▼^ |  | 491.2 | 2 | EICECDR | Acont_PLA2-4_e312 | PLA2 |  |
|  |  |  |  | 404.7 | 2 | AAAICFR |  |  |  |
|  | 0.03 | 14^▼^ |  | 549.3 | 2 | FLALVYTDR | Acont_SVSP-8_e14 | SVSP |  |
| **24** | 0.85 | 50^▼^ |  | 773.1 | 3 | NSEHIAPLSLPSSPPIVGSVCR | Acont_SVSP-13a_e537 | SVSP |  |
|  |  |  |  | 739.7 | 3 | DSGGPLICNGQFQGIVSWGPK |  |  |  |
|  |  |  |  | 565.3 | 2 | LLNEDEQIR | Acont_SVSP-4_e9 | SVSP |  |
|  | 1.98 | 31^▼^ |  | 629.3 | 2 | KLLNEDEQIR | Acont_SVSP-4_e9 | SVSP |  |
|  |  |  |  | 532.9 | 3 | KLLNEDEQIRNPK |  |  |  |
|  |  |  |  | 565.3 | 2 | LLNEDEQIR |  |  |  |
| **26** | 0.40 | 36^▼^ |  | 595.3 | 2 | WDKDIMLIR | Acont_SVSP-6_98-10M191 | SVSP |  |
|  |  |  |  | 559.8 | 2 | TLCAGILEGGK |  |  |  |
|  |  |  |  | 404.2 | 2 | EPGLYTK |  |  |  |
|  |  |  |  | 570.8 | 2 | AANPELPATTR | Acont_SVSP-7_e420 | SVSP |  |
|  |  |  |  | 487.2 | 2 | YFCLNTR | Acont_SVSP-16a_e369(982) | SVSP | * |
|  | 0.80 | 33^▼^ |  | 476.9 | 3 | GNMLIFLGVHSLK | Acont_SVSP-7_e420 | SVSP |  |
|  |  |  |  | 497.6 | 3 | DDEKDKDIMLIR |  |  |  |
|  |  |  |  | 502.3 | 2 | DKDIMLIR |  |  |  |
|  |  |  |  | 570.8 | 2 | AANPELPATTR |  |  |  |
|  |  |  |  | 559.8 | 2 | TLCAGILEGGK |  |  |  |
|  |  |  |  | 500.2 | 3 | VVGGDECNINEHR | Acont_SVSP-3a_e242(9,14,515,339) | SVSP | * |
|  |  |  |  | 601.3 | 2 | NVPNEDEQTR | Acont_SVSP-6_98-10M191 | SVSP |  |
|  | 0.40 | 31^▼^ |  | 476.9 | 3 | GNMLIFLGVHSLK | Acont_SVSP-7_e420 | SVSP |  |
|  |  |  |  | 502.3 | 2 | DKDIMLIR |  |  |  |
|  |  |  |  | 570.8 | 2 | AANPELPATTR |  |  |  |
|  |  |  |  | 559.8 | 2 | TLCAGILEGGK |  |  |  |
|  |  |  |  | 601.3 | 2 | NVPNEDEQTR | Acont_SVSP-6_98-10M191 | SVSP |  |
|  |  |  |  | 565.3 | 2 | LLNEDEQIR | Acont_SVSP-4_e9 | SVSP |  |
| **29** | 1.84 | 55^▼^ |  | 757.8 | 2 | ETDYEEFLEIAR | Acont_LAAO-1_e495 | LAAO |  |
|  |  |  |  | 684.3 | 2 | EDWYANLGPMoxR |  |  |  |
|  |  |  |  | 524.6 | 3 | (DPGLLE)YPVKPSEK |  |  |  |
|  |  |  |  | 643.8 | 2 | SAGQLYEESFR |  |  |  |
|  |  |  |  | 562.3 | 2 | HDDIFGYEK |  |  |  |
|  | 0.29 | 50^▼^ |  | 757.8 | 2 | ETDYEEFLEIAR | Acont_LAAO-1_e495 | LAAO |  |
|  |  |  |  | 524.6 | 3 | (DPGLLE)YPVKPSEK |  |  |  |
|  |  |  |  | 562.3 | 2 | HDDIFGYEK |  |  |  |
|  |  |  |  | 557.8 | 2 | VIEIQQNDR |  |  |  |
|  |  |  |  | 583.4 | 2 | IKFEPPLPPK |  |  |  |
|  |  |  |  | 441.7 | 2 | IFLTCTK |  |  |  |
|  | 0.18 | 50^▼^ |  | 684.8 | 2 | LYCFPNSPENK | Acont_SVMPIII-6a_e457 | SVMP |  |
|  |  |  |  | 532.7 | 2 | NPLEECFR |  |  |  |
| **31** | 0.12 | >66^▼^ |  | 408.7 | 2 | ATDLLKR | Acont_SVMPIII-6a_e457 | SVMP |  |
|  |  |  |  | 493.3 | 2 | EHQAFLIK |  |  |  |
|  |  |  |  | 842.8 | 2 | GAQCAEGLCCDQCR |  |  |  |
|  |  |  |  | 571.6 | 3 | AAKDECDMADLCTGR |  |  |  |
|  |  |  |  | 684.8 | 2 | LYCFPNSPENK |  |  |  |
|  |  |  |  | 502.3 | 2 | NMPQCILK |  |  |  |
|  | 0.12 | 66^▼^ |  | 578.6 | 3 | MYDIVNVITPIYHR | Acont_SVMPIII-6a_e457 | SVMP |  |
|  |  |  |  | 502.3 | 2 | NMPQCILK |  |  |  |
|  |  |  |  | 842.8 | 2 | GAQCAEGLCCDQCR |  |  |  |
|  |  |  |  | 571.6 | 3 | AAKDECDMADLCTGR |  |  |  |
|  |  |  |  | 684.8 | 2 | LYCFPNSPENK |  |  |  |
|  | 0.02 | 55^▼^ |  | 842.8 | 2 | GAQCAEGLCCDQCR | Acont_SVMPIII-6a_e457 | SVMP |  |
|  |  |  |  | 493.3 | 2 | EHQAFLIK |  |  |  |
|  |  |  |  | 532.7 | 2 | NPLEECFR | Acont_LAAO-1_e495 | LAAO |  |
|  |  |  |  | 757.8 | 2 | ETDYEEFLEIAR |  |  |  |
|  |  |  |  | 524.6 | 3 | DPGLLEYPVKPSEK |  |  |  |
|  |  |  |  | 557.8 | 2 | VIEIQQNDR |  |  |  |
|  |  |  |  | 438.7 | 2 | STTDLPSR |  |  |  |
|  | 0.11 | 55^▼^ |  |  |  |  |  |  |  |
|  | 1.84 | 50^▼^ |  | 578.6 | 3 | MYDIVNVITPIYHR | Acont_SVMPIII-6a_e457 | SVMP |  |
|  |  |  |  | 690.7 | 3 | MNIHVALVSLEIWSNTDK |  |  |  |
|  |  |  |  | 720.9 | 2 | LVLVADYIMYLK |  |  |  |
|  |  |  |  | 842.8 | 2 | GAQCAEGLCCDQCR |  |  |  |
|  |  |  |  | 602.3 | 2 | NNNGYCYNGK |  |  |  |
|  |  |  |  | 577.7 | 2 | QGNHYGYCR |  |  |  |
|  |  |  |  | 684.8 | 2 | LYCFPNSPENK |  |  |  |
|  | 0.12 | 36^▼^ |  | 728.9 | 2 | LVLVADYIMoxYLK | Acont_SVMPIII-6a_e457 | SVMP |  |
|  |  |  |  | 578.6 | 3 | MYDIVNVITPIYHR |  |  |  |
|  |  |  |  | 684.8 | 2 | LYCFPNSPENK |  |  |  |
|  |  |  |  | 453.2 | 2 | YEGDKTEICSR | Acont_SVMPII-1_me196 | SVMP |  |
|  | 0.12 | 21^▼^ |  | 720.9 | 2 | LVLVADYIMYLK | Acont_SVMPIII-6a_e457 | SVMP |  |
|  |  |  |  | 578.6 | 3 | MYDIVNVITPIYHR |  |  |  |
|  |  |  |  | 684.8 | 2 | LYCFPNSPENK |  |  |  |
| **32** | 0.29 | 55^▼^ |  | 532.7 | 2 | NPLEECFR | Acont_LAAO-1_e495 | LAAO |  |
|  |  |  |  | 757.8 | 2 | ETDYEEFLEIAR |  |  |  |
|  |  |  |  | 557.8 | 2 | VIEIQQNDR |  |  |  |
|  |  |  |  | 438.7 | 2 | STTDLPSR |  |  |  |
|  |  |  |  | 571.3 | 3 | (DCGD)IVINDLSLIHK |  |  |  |
|  | 0.28 | 50^▼^ |  | 720.9 | 2 | LVLVADYIMYLK | Acont_SVMPIII-6a_e457 | SVMP |  |
|  |  |  |  | 867.5 | 2 | MYDIVNVITPIYHR |  |  |  |
|  |  |  |  | 493.3 | 2 | EHQAFLIK |  |  |  |
|  |  |  |  | 529.8 | 2 | IACEPQNVK |  |  |  |
|  |  |  |  | 684.8 | 2 | LYCFPNSPENK |  |  |  |
|  | 0.26 | 33^▼^ |  | 438.6 | 3 | YVELVIVADHR | Acont_SVMPII-1_me196(592) | SVMP | * |
|  |  |  |  | 620.3 | 2 | NNNGYCYNGK | Acont_SVMPIII-6a_e457 | SVMP |  |
|  |  |  |  | 577.6 | 3 | HDNAQLLTGMIFNEK | Acont_SVMPII-1_me196(592) | SVMP | * |
|  |  |  |  | 493.3 | 2 | EHQAFLIK | Acont_SVMPIII-6a_e457 | SVMP |  |
|  | 0.02 | 33^▼^ |  | 766.4 | 2 | GLAATTLCAGILEGGK | Acont_SVSP-16a_e369(982) | SVSP | * |
| **33** | 0.47 | 57^▼^ |  | 714.4 | 3 | SHDNAQLLTVIDFDGPTIGK | Acont_SVMPIII-3_1f45 | SVMP |  |
|  |  |  |  | 992.4 | 2 | LTPGSQCADGVCCDQCR |  |  |  |
|  |  |  |  | 585.3 | 2 | KIPCAPQDIK |  |  |  |
|  |  |  |  | 521.3 | 2 | IPCAPQDIK |  |  |  |
|  | 0.37 | 55^▼^ |  | 532.7 | 2 | NPLEECFR | Acont_LAAO-1_e495 | LAAO |  |
|  |  |  |  | 757.8 | 2 | ETDYEEFLEIAR |  |  |  |
|  |  |  |  | 524.6 | 3 | DPGLLEYPVKPSEK |  |  |  |
|  |  |  |  | 643.8 | 2 | SAGQLYEESFR |  |  |  |
|  |  |  |  | 562.3 | 2 | HDDIFGYEK |  |  |  |
|  |  |  |  | 557.8 | 2 | VIEIQQNDR |  |  |  |
|  |  |  |  | 583.3 | 2 | IKFEPPLPPK |  |  |  |
|  |  |  |  | 441.7 | 2 | IFLTCTK |  |  |  |
|  | 0.10 | 55^▼^ |  | 565.3 | 2 | ENGVNIPCAR | Acont_SVMPIII-2_me561 | SVMP |  |
|  |  |  |  | 752.3 | 2 | LFCEFNNFPCR |  |  |  |
|  | 0.08 | 50^▼^ |  | 524.6 | 3 | DPGLLEYPVKPSEK | Acont_LAAO-1_e495 | LAAO |  |
|  |  |  |  | 557.8 | 2 | VIEIQQNDR |  |  |  |
|  |  |  |  | 441.7 | 2 | IFLTCTK |  |  |  |
|  | 0.06 | 50^▼^ |  | 585.3 | 2 | KIPCAPQDIK | Acont_SVMPIII-3_1f45 | SVMP |  |
|  |  |  |  | 521.3 | 2 | IPCAPQDIK |  |  |  |
|  | 0.14 | 31^▼^ |  | 570.8 | 2 | AANPELPATTR | Acont_SVSP-7_e420 | SVSP |  |
|  |  |  |  | 559.8 | 2 | TLCAGILEGGK |  |  |  |
|  |  |  |  | 741.7 | 3 | NSAHIAPLSLPSSSPSVGSVCR | Acont_SVSP-10_98-10M279 | SVSP |  |
|  | 0.14 | 25^▼^ |  | 595.3 | 2 | TAIVFDDGIIGR | Acont_SVMPI-4a_e575 | SVMP |  |
| **35** | 0.66 | 55^▼^ |  | 757.8 | 2 | ETDYEEFLEIAR | Acont_LAAO-1_e495 | LAAO |  |
|  |  |  |  | 532.3 | 3 | DKEDWYANLGPMR |  |  |  |
|  |  |  |  | 524.6 | 3 | DPGLLEYPVKPSEK |  |  |  |
|  |  |  |  | 643.8 | 2 | SAGQLYEESFR |  |  |  |
|  |  |  |  | 557.8 | 2 | VIEIQQNDR |  |  |  |
|  |  |  |  | 441.7 | 2 | IFLTCTK |  |  |  |
|  |  |  |  | 438.7 | 2 | STTDLPSR |  |  |  |
|  | 0.10 | 55^▼^ |  | 430.3 | 2 | KTDLLNR | Acont_SVMPI-3a_e522 | PI-SVMP |  |
|  |  |  |  | 723.7 | 3 | SHDNAQLLTAIVFDEGIIGR |  |  |  |
|  | 0.76 | 36^▼^ |  | 753.3 | 2 | AYQECMCFPQR | Acont_SVMPII-6a_e592 | SVMP |  |
|  |  |  |  | 935.8 | 2 | GDDVDDYCNGISAGCPR |  |  |  |
|  |  |  |  | 430.3 | 2 | KTDLLNR | Acont_SVMPI-3a_e522 | PI-SVMP |  |
|  |  |  |  | 723.7 | 3 | SHDNAQLLTAIVFDEGIIGR |  |  |  |
|  | 10.64 | 25^▼^ |  | 438.6 | 3 | YVELVIVADHR | Acont_SVMPI-3a_e522 | SVMP |  |
|  |  |  |  | 534.3 | 2 | YNGDSDKIR |  |  |  |
|  |  |  |  | 454.2 | 4 | YNGDSDKIRQWIYR |  |  |  |
|  |  |  |  | 1133.0 | 2 | DLSTVTSVSHDTLASFENWR |  |  |  |
|  |  |  |  | 723.7 | 3 | SHDNAQLLTAIVFDEGIIGR |  |  |  |
|  |  |  |  | 709.9 | 2 | VSLTDLEVWSNR | Acont_SVMPII-5a_e416 | SVMP |  |
|  | 0.76 | 18^▼^ |  | 657.4 | 2 | YVELVIVADHR | Acont_SVMPI-3a_e522 | SVMP |  |
|  |  |  |  | 755.7 | 3 | DLSTVTSVSHDTLASFENWR |  |  |  |
|  |  |  |  | 430.3 | 2 | KTDLLNR |  |  |  |
|  |  |  |  | 723.7 | 3 | SHDNAQLLTAIVFDEGIIGR |  |  |  |
|  |  |  |  | 547.8 | 2 | YNSNLNTIR | Acont_SVMPII-5a_e416 | SVMP |  |
|  |  |  |  | 438.3 | 3 | YVELVIVADHR |  | SVMP | * |
|  | 0.76 | 14^▼^ |  | 534.3 | 2 | YNGDSDKIR | Acont_SVMPI-3a_e522 | SVMP |  |
|  |  |  |  | 755.7 | 3 | DLSTVTSVSHDTLASFENWR |  |  |  |
|  |  |  |  | 430.3 | 2 | KTDLLNR |  |  |  |
|  |  |  |  | 723.7 | 3 | SHDNAQLLTAIVFDEGIIGR |  |  |  |
|  | 0.76 | 8^▼^ |  | 534.3 | 2 | YNGDSDKIR | Acont_SVMPI-3a_e522 | SVMP |  |
|  |  |  |  | 755.7 | 3 | DLSTVTSVSHDTLASFENWR |  |  |  |
|  |  |  |  | 430.3 | 2 | KTDLLNR |  |  |  |
|  |  |  |  | 723.7 | 3 | SHDNAQLLTAIVFDEGIIGR |  |  |  |
|  |  |  |  | 547.8 | 2 | YNSNLNTIR | Acont_SVMPII-5a_e416 | SVMP |  |
|  |  |  |  | 709.9 | 2 | VSLTDLEVWSNR |  |  |  |
|  | 0.76 | 6^▼^ |  | 534.3 | 2 | YNGDSDKIR | Acont_SVMPI-3a_e522 | SVMP |  |
|  |  |  |  | 755.7 | 3 | DLSTVTSVSHDTLASFENWR |  |  |  |
|  |  |  |  | 430.3 | 2 | KTDLLNR |  |  |  |
|  |  |  |  | 723.7 | 3 | SHDNAQLLTAIVFDEGIIGR |  |  |  |
| **36** | 0.23 | 57^▼^ |  | 532.7 | 2 | NPLEECFR | Acont_LAAO-1_e495 | LAAO |  |
|  |  |  |  | 757.8 | 2 | ETDYEEFLEIAR |  |  |  |
|  |  |  |  | 532.2 | 3 | DKEDWYANLGPMR |  |  |  |
|  |  |  |  | 557.8 | 2 | VIEIQQNDR |  |  |  |
|  |  |  |  | 438.6 | 2 | STTDLPSR |  |  |  |
|  | 0.10 | 57^▼^ |  | 560.8 | 2 | RGDIGIAYGAK | Acont_SVMPI-2a_e44 | SVMP |  |
|  |  |  |  | 482.7 | 2 | GDIGIAYGAK |  |  |  |
|  | 0.33 | 55^▼^ |  | 430.3 | 2 | QTDLLNR | Acont_SVMPI-2a_e44 | SVMP |  |
|  |  |  |  | 560.8 | 2 | RGDIGIAYGAK |  |  |  |
|  |  |  |  | 482.7 | 2 | GDIGIAYGAK |  |  |  |
|  |  |  |  | 538.3 | 2 | IFPCAPQDK | Acont_SVMPIII-7_98-5M50 | SVMP |  |
|  | 1.33 | 31^▼^ |  | 438.6 | 2 | STTDLPSR | Acont_SVMPI-2a_e44 | SVMP |  |
|  |  |  |  | 482.7 | 2 | GDIGIAYGAK |  |  |  |
|  |  |  |  | 406.2 | 3 | FSVGIVQDHSK |  |  |  |
|  |  |  |  | 723.7 | 3 | SHDNAQLLTAIVFDEGIIGR | Acont_SVMPI-3a_e522 | SVMP |  |
|  | 2.66 | 26^▼^ |  | 657.4 | 2 | YVELVIVADHR | Acont_SVMPI-2a_e44 | SVMP |  |
|  |  |  |  | 430.2 | 2 | QTDLLNR |  |  |  |
|  |  |  |  | 482.7 | 2 | GDIGIAYGAK |  |  |  |
|  |  |  |  | 608.8 | 2 | FSVGIVQDHSK |  |  |  |
|  | 1.33 | 23^▼^ |  | 534.3 | 2 | YNGDSDKIR | Acont_SVMPI-3a_e522 | SVMP |  |
|  |  |  |  | 723.7 | 3 | SHDNAQLLTAIVFDEGIIGR |  |  |  |
|  |  |  |  | 755.7 | 3 | DLSTVTSVSHDTLASFENWR |  |  |  |
|  |  |  |  | 430.2 | 2 | QTDLLNR | Acont_SVMPI-2a_e44 | SVMP |  |
|  |  |  |  | 482.7 | 2 | GDIGIAYGAK |  |  |  |
|  |  |  |  | 406.2 | 3 | FSVGIVQDHSK |  |  |  |
|  |  |  |  | 626.8 | 2 | MVNTINEIYR | Acont_SVMPI-3a_e522 | SVMP |  |
|  |  |  |  | 438.3 | 3 | YVELVIVADHR |  | SVMP |  |
|  | 0.33 | 16^▼^ |  | 657.4 | 2 | YVELVIVADHR | Acont_SVMPI-2a_e44 | SVMP |  |
|  |  |  |  | 430.2 | 2 | QTDLLNR |  |  |  |
|  |  |  |  | 482.7 | 2 | GDIGIAYGAK |  |  |  |
|  |  |  |  | 461.7 | 2 | GVIGSAYGAK | Acont_SVMPII-2_me463 |  |  |
|  | 0.33 | 6^▼^ |  | 406.2 | 3 | FSVGIVQDHSK |  |  |  |
|  |  |  |  | 521.8 | 2 | KDYQTFLK |  |  |  |
| **37** | 0.20 | 57^▼^ |  | 643.8 | 2 | SAGQLYEESFR | Acont_LAAO-1_e495 | LAAO |  |
|  |  |  |  | 557.8 | 2 | VIEIQQNDR |  |  |  |
|  |  |  |  | 430.2 | 3 | FWEEEGIHGGK |  |  |  |
|  | 0.03 | 57^▼^ |  | 626.8 | 2 | MVNTINEIYR | Acont_SVMPI-4a_e575(522) | SVMP | * |
|  | 0.23 | 55^▼^ |  | 615.3 | 2 | LVIVADDVMVR | Acont_SVMPIII-4_98-1M61 | SVMP |  |
|  |  |  |  | 430.2 | 2 | KTDLLNR |  |  |  |
|  |  |  |  | 587.3 | 2 | IFPCAPQNVK |  |  |  |
|  |  |  |  | 626.8 | 2 | MVNTINEIYR | Acont_SVMPI-4a_e575(522) | SVMP | * |
|  | 0.23 | 31^▼^ |  | 626.8 | 2 | MVNTINEIYR | Acont_SVMPI-4a_e575 | SVMP |  |
|  | 0.23 | 25^▼^ |  | 430.2 | 2 | QTDLLNR |  |  |  |
|  |  |  |  | 482.7 | 2 | GDIGIAYGAK | Acont_SVMPI-2a_e44 | SVMP |  |
|  |  |  |  | 626.8 | 2 | MVNTINEIYR | Acont_SVMPI-4a_e575 | SVMP |  |
|  | 2.77 | 23^▼^ |  | 443.3 | 3 | YVELVIIADHR | Acont_SVMPI-4a_e575 | SVMP |  |
|  |  |  |  | 533.8 | 2 | YNGDSNKIR |  |  |  |
|  |  |  |  | 517.8 | 2 | IRQWIYR |  |  |  |
|  |  |  |  | 626.8 | 2 | MVNTINEIYR |  |  |  |
|  |  |  |  | 601.3 | 2 | APLAGMCDPNR |  |  |  |
|  |  |  |  | 529.3 | 2 | SVGTVQDHSK |  |  |  |
|  |  |  |  | 514.3 | 2 | KDYLTFLK |  |  |  |
|  | 0.23 | 18^▼^ |  | 626.8 | 2 | MVNTINEIYR | Acont_SVMPI-4a_e575 | SVMP |  |
|  |  |  |  | 588.3 | 2 | VFDDGIIGR |  |  |  |
|  | 0.23 | 16^▼^ |  | 626.8 | 2 | MVNTINEIYR | Acont_SVMPI-4a_e575 | SVMP |  |
|  |  |  |  | 588.3 | 2 | VFDDGIIGR |  |  |  |
|  | 0.23 | 8^▼^ |  | 626.8 | 2 | MVNTINEIYR | Acont_SVMPI-4a_e575 | SVMP |  |
|  |  |  |  | 588.3 | 2 | VFDDGIIGR |  |  |  |
|  |  |  |  | 638.5 | 2 | TAIVFDDGIIGR |  |  |  |
|  | 0.23 | 6^▼^ |  | 626.8 | 2 | MVNTINEIYR | Acont_SVMPI-4a_e575 | SVMP |  |
|  |  |  |  | 514.3 | 2 | KDYLTFLK |  |  |  |

**Table S3.** Identification by MS/MS of the venom components of *Agkistrodon c. contortrix* specimen F19 (unrelated female) (Fig. 2B). *, ambiguous ID.

| **Spot ID** | **%** | **MW (kDa)** | **ESI-MS (ave)** | **m/z** | **z** | **Peptide sequence** | **Transcriptome match** | **Protein family** | **Observations** |
| --- | --- | --- | --- | --- | --- | --- | --- | --- | --- |
|  |  |  |  |  |  |  |  |  |  |
| **2** | 0.93 |  |  | 592.3 | 2 | ZWPPGHHIPP | Acont_BPP-1a_98 | VAP (BPP) |  |
| **3** | 1.15 |  |  | 532.3 | 2 | TPPAGPDVGPR | Acont_BPP-1a_98 | VAP (BIP) |  |
| **4** | 0.89 |  |  |  |  |  |  | not identified |  |
| **5** | 3.15 |  |  | 430.2 | 1 | ZNW | Acont_BPP-1a_98 | SVMPi |  |
| **6** | 2.01 |  |  | 444.2 | 1 | ZQW | Acont_BPP-1a_98 | SVMPi |  |
| **7** | 0.21 |  |  | 576.2 | 2 | CTGQSADCPR | Acont_SVMPII-3a_e261-2 (=me463) | Long Disintegrin JAS04330 |  |
| **8** | 0.11 | 21▪ |  | 598.8 | 2 | NPCCDAATCK | Acont_DIS-2a_e351 | Dimeric Disintegrin |  |
|  |  |  |  | 992.4 | 2 | LTPGSQCAEGLCCDQCK |  |  |  |
|  |  |  |  | 569.2 | 2 | CTGQSGDCPR |  |  |  |
|  |  | 8▼ | 8771.6 | 569.2 | 2 | CTGQSGDCPR | Acont_SVMPII-1_me196 | Long Disintegrin JAS04331 | Mav calc (SS) ^1^VS..NA^84^: 8772,8 |
|  |  |  |  | 942.9 | 2 | GDDLDDYCNGISAGCPR | Acont_SVMPII-5a_e416 | Dimeric Disintegrin |  |
| **9** | 1.95 | 9▼ | 13506.7 |  |  |  | Acont_DIS-2a_e351 | Dimeric Disintegrin | Mav calc (SS): 6806,8  Mav calc: heterodimer e351/e866: 13511,7 |
|  |  |  |  | 598.7 | 2 | NPCCDAATCK | Acont_DIS-1_e866 | JAS04370 (Disi 3) | Mav calc (SS): 6724,9 |
|  |  |  |  | 992.4 | 2 | LTPGSQCAEGLCCDQCK |  |  |  |
|  |  |  |  | 418.7 | 2 | GDNPDYR |  |  |  |
|  | 0.09 | 9▼ |  | 558.3 | 2 | VLNEDEQIR | Acont_SVSP-3a_e242 | SVSP |  |
|  |  |  |  | 559.8 | 2 | TLCAGILEGGK |  |  |  |
| **10** |  |  | 13506.7 | 992.4 | 2 | LTPGSQCAEGLCCDQCK | Acont_DIS-2a_e351 | Dimeric Disintegrin |  |
|  |  |  |  |  |  |  | Acont_DIS-1_e866 | JAS04370 (Disintegrin 3) |  |
| **15** | 1.79 | 30▼ |  | 460.2 | 2 | MILQETGK | Acont_PLA2-3a_e604 | PLA2 |  |
|  |  |  |  | 431.7 | 2 | AIICEEK |  |  |  |
|  |  |  |  | 401.7 | 2 | AVAICLR |  |  |  |
|  |  |  |  | 498.7 | 2 | ENLDTYNK |  |  |  |
|  |  |  |  | 562.8 | 2 | ENLDTYNKK |  |  |  |
|  |  |  |  | 416.2 | 2 | YNPYFK |  |  |  |
|  | 17.86 | 14▼ | 14043.1 | 468.2 | 2 | MoxILQETGK | Acont_PLA2-3a_e604 | PLA2 | Mav calc: 14043.3 |
|  |  |  |  | 707.3 | 3 | NAITSYGFYGCNCGWGHR |  |  |  |
|  |  |  |  | 454.6 | 3 | AIICEEKNPCK |  |  |  |
|  |  |  |  | 585.6 | 3 | EMCECDKAVAICLR |  |  |  |
|  |  |  |  | 480.2 | 2 | KYNPYFK |  |  |  |
|  | 0.20 | 11▼ |  | 460.2 | 2 | MILQETGK | Acont_PLA2-3a_e604 | PLA2 |  |
|  |  |  |  | 431.7 | 2 | AIICEEK |  |  |  |
|  |  |  |  | 401.7 | 2 | AVAICLR |  |  |  |
|  |  |  |  | 498.7 | 2 | ENLDTYNK |  |  |  |
|  |  |  |  | 562.8 | 2 | ENLDTYNKK |  |  |  |
| **16** | 0.22 | 15▼ | 14043.1 | 460.2 | 2 | MILQETGK | Acont_PLA2-3a_e604 | PLA2 |  |
|  |  |  |  | 401.7 | 2 | AVAICLR |  |  |  |
|  |  |  |  | 498.7 | 2 | ENLDTYNK |  |  |  |
|  |  |  |  | 562.8 | 2 | ENLDTYNKK |  |  |  |
|  |  |  |  | 551.3 | 2 | LDIYTYSVK | Acont_PLA2-1a_e343 | PLA2 |  |
|  | 0.07 | 13▼ |  | 460.2 | 2 | MILQETGK | Acont_PLA2-3a_e604 | PLA2 |  |
|  |  |  |  | 401.7 | 2 | AVAICLR |  |  |  |
|  |  |  |  | 562.8 | 2 | ENLDTYNKK |  |  |  |
| **18** | 0.73 | 32▼ |  | 565.3 | 2 | FLVALYTFR | Acont_SVSP-5_e565 | SVSP |  |
|  |  |  |  | 507.8 | 2 | AAYPQLPVR |  |  |  |
|  |  |  |  | 536.9 | 3 | TLCAGILEGGKDSCK |  |  |  |
|  | 0.73 | 27▼ | 24822.9 | 565.3 | 2 | FLVALYTFR | Acont_SVSP-5_e565 | SVSP |  |
|  | 0.42 | 15▼ |  | 521.5 | 3 | CCFVHDCCYNK | Acont_PLA2-1a_e343 | PLA2 |  |
|  |  |  |  | 551.3 | 2 | LDIYTYSVK |  |  |  |
|  |  |  |  | 603.8 | 2 | DVVCGGTNPCK |  |  |  |
|  |  |  |  | 404.7 | 2 | AAAICFR |  |  |  |
|  |  |  |  | 460.2 | 2 | MILQETGK | Acont_PLA2-3a_e604 | PLA2 |  |
|  |  |  |  | 401.7 | 2 | (A)VAICLR |  |  |  |
|  | 0.00 | 15▼ |  | 507.8 | 2 | (AA)YPQLPVR | Acont_SVSP-5_e565 | SVSP |  |
|  | 0.09 | 12▼ |  | 507.8 | 2 | (AA)YPQLPVR | Acont_SVSP-5_e565 | SVSP |  |
|  |  |  |  | 559.8 | 2 | TLCAGILEGGK |  |  |  |
|  | 0.12 | 12▼ |  | 551.3 | 2 | LDIYTYSVK | Acont_PLA2-1a_e343 | PLA2 |  |
| **19** | 0.08 | 40▼ |  | 519.3 | 2 | AFYPGLLEK | Acont_SVSP-12a_98-10M126 | SVSP |  |
|  |  |  |  | 727.3 | 2 | AAYPEYDLPATSR | Acont_SVSP-15a_e914 | SVSP |  |
|  |  |  |  | 559.8 | 2 | TLCAGILEGGK |  |  |  |
|  |  |  |  | 571.8 | 2 | FLVALYNFR | Acont_SVSP-14a_e824 | SVSP |  |
|  | 0.02 | 40▼ |  | 551.3 | 2 | LDIYTYSVK | Acont_PLA2-1a_e343 | PLA2 |  |
|  | 1.01 | 32▼ |  | 750.9 | 2 | LPATTLCAGILEGGK | Acont_SVSP-17a_e339 | SVSP |  |
|  |  |  |  | 403.7 | 2 | KPGLYTK |  |  |  |
|  |  |  |  | 500.2 | 3 | VVGGDECNINEHR | Acont_SVSP-3a_e(242,9,339,515) | SVSP | * |
|  |  |  |  | 559.8 | 2 | TLCAGILEGGK |  |  |  |
|  | 0.40 | 16▼ |  | 502.5 | 3 | CCFVHDCCYGK | Acont_PLA2-4_e312 | PLA2 |  |
|  |  |  |  | 491.2 | 2 | EICECDR |  |  |  |
|  | 8.49 | 15▼ | 14163.9 | 630.0 | 3 | VTSCNPKLDIYTYSVK | Acont_PLA2-1a_e343 | PLA2 | Mave calc: 14164,1 |
|  |  |  |  | 551.3 | 2 | LDIYTYSVK |  |  |  |
|  |  |  |  | 404.7 | 2 | AAAICFR |  |  |  |
|  | 0.10 | 9▼ |  | 521.5 | 3 | (CC)FVHDCCYNK | Acont_PLA2-1a_e343 | PLA2 |  |
|  |  |  |  | 603.8 | 2 | DVVCGGTNPCK |  |  |  |
|  |  |  |  | 404.7 | 2 | AAAICFR |  |  |  |
| **20** | 0.03 | 40▼ |  | 544.3 | 2 | VPNEDEQTR | Acont_SVSP-15a_e914 | SVSP |  |
|  |  |  |  | 727.3 | 2 | AAYPEYDLPATSR |  |  |  |
|  |  |  |  | 559.8 | 2 | TLCAGILEGGK |  |  |  |
|  |  |  |  | 550.9 | 3 | TLCAGILEGGKDTCR |  |  |  |
|  |  |  |  | 571.8 | 2 | FLVALYNFR | Acont_SVSP-14a_e824 | SVSP |  |
|  |  |  |  | 519.3 | 2 | AFYPGLLEK | Acont_SVSP-12a_98-10M126 | SVSP |  |
|  |  |  |  | 403.7 | 2 | PGLYTK | Acont_SVSP-7_e(339,369,515,982,10M191) | SVSP | * |
|  | 2.33 | 31▼ |  | 404.7 | 2 | AAAICFR | Acont_PLA2-1a_e343 | PLA2 |  |
|  | 0.09 | 16▼ |  | 596.8 | 2 | IMGWGTTTPTK | Acont_SVSP-2a_e957 | SVSP |  |
|  | 0.05 | 16▼ |  | 551.3 | 2 | LDIYTYSVK | Acont_PLA2-1a_e343 | PLA2 |  |
|  | 0.41 | 15▼ | 14163.9/ 13769.7 | 551.3 | 2 | LDIYTYSVK | Acont_PLA2-1a_e343 | PLA2 |  |
|  |  |  |  | 490.7 | 2 | QICECDR |  |  |  |
|  |  |  |  | 404.7 | 2 | AAAICFR |  |  |  |
|  |  |  |  | 445.5 | 2 | DVVCGGTNPCKK |  |  |  |
|  | 0.00 | 15▼ |  | 596.8 | 2 | IMGWGTTTPTK | Acont_SVSP-2a_e957 | SVSP |  |
|  | 0.41 | 15▼ |  | 502.7 | 2 | KAWEDAER | Acont_CTL-9a_e4 | CTL |  |
|  |  |  |  | 701.4 | 2 | GQAEVWIGLWDK |  |  |  |
|  |  |  |  | 621.3 | 2 | DFSWEWTDR |  |  |  |
|  |  |  |  | 644.8 | 2 | SCTDYLTWDK |  |  |  |
|  |  |  |  | 786.9 | 2 | EFCVELVSLTGYR |  |  |  |
| **22** | 3.33 | 35▼ |  | 486.3 | 2 | IYLGVHNR | Acont_SVSP-16a_e369 | SVSP |  |
|  |  |  |  | 766.4 | 2 | GLAATTLCAGILEGGK |  |  |  |
|  |  |  |  | 544.8 | 2 | IYLGVHNR | Acont_SVSP-18a_e982 | SVSP |  |
|  |  |  |  | 750.7 | 3 | NSAHIAPLSLPSNSPSVGSVCR |  |  |  |
|  |  |  |  | 571.8 | 2 | FLVALYNFR | Acont_SVSP-14a_e824 | SVSP |  |
|  |  |  |  | 544.3 | 2 | VPNEDEQTR |  |  |  |
|  |  |  |  | 559.8 | 2 | TLCAGILEGGK |  |  |  |
|  |  |  |  | 727.3 | 2 | AAYPEYDLPATSR | Acont_SVSP-15a_e914 | SVSP |  |
|  | 0.40 | 32▼ |  | 486.3 | 2 | IYLGVHNR | Acont_SVSP-16a_e369 | SVSP |  |
|  |  |  |  | 766.4 | 2 | GLAATTLCAGILEGGK |  |  |  |
|  |  |  |  | 750.7 | 3 | NSAHIAPLSLPSNSPSVGSVCR | Acont_SVSP-18a_e982 | SVSP |  |
|  |  |  |  | 750.9 | 2 | LPATTLCAGILEGGK | Acont_SVSP-17a_e339 | SVSP |  |
|  |  |  |  | 500.2 | 3 | VVGGDECNINEHR | Acont_SVSP-3a_e(242,9,339,515) | SVSP | * |
|  |  |  |  | 559.8 | 2 | TLCAGILEGGK |  |  |  |
|  |  |  |  | 549.3 | 2 | FLALVYTDR | Acont_SVSP-8_e14 | SVSP |  |
|  |  |  |  | 596.8 | 2 | IMGWGTTTPTK | Acont_SVSP-2a_e957 | SVSP |  |
|  | 9.33 | 15▼ | 13753.4 | 491.2 | 2 | EICECDR | Acont_PLA2-4_e312 | PLA2 | Mave calc: 13753,4 |
|  |  |  |  | 404.7 | 2 | AAAICFR |  |  |  |
|  | 0.13 | 9▼ |  | 654.3 | 2 | NFQMLFGVHSK | Acont_SVSP-2a_e957 | SVSP |  |
|  |  |  |  | 596.8 | 2 | IMGWGTTTPTK |  |  |  |
|  |  |  |  | 624.3 | 4 | ETYPDVPHCANINLLDHAVCR |  |  |  |
|  |  |  |  | 1126.6 | 2 | AAYPELLAESSTLCAGTQQGGK |  |  |  |
|  |  |  |  | 963.5 | 3 | LNSPVSNSEHIAPLSLPSSPPSVGSVCR |  |  |  |
|  | 0.13 | 5▼ |  | 404.7 | 2 | AAAICFR | Acont_PLA2-4_e(312,343,if2202) | PLA2 | * |
| **23** | 0.03 | 45▼ |  | 803.4 | 2 | ILCAGVLEGGIDTCK | Acont_SVSP-13a_e537 | SVSP |  |
|  |  |  |  | 587.9 | 3 | ILCAGVLEGGIDTCKR |  |  |  |
|  |  |  |  | 403.7 | 2 | KPALYSK |  |  |  |
|  | 0.03 | 35▼ |  | 466.7 | 2 | YFCLSSR | Acont_SVSP-10_98-10M279 | SVSP |  |
|  |  |  |  | 797.4 | 2 | TLCAGILEGGIDSCK |  |  |  |
|  |  |  |  | 741.7 | 3 | NSAHIAPLSLPSNSPSVGSVCR |  |  |  |
|  |  |  |  | 486.3 | 2 | IYLGVHNR | Acont_SVSP-16a_e(339,369,515) | SVSP | * |
|  |  |  |  | 750.7 | 3 | NSAHIAPLSLPSNSPSVGSVCR | Acont_SVSP-18a_e982 | SVSP |  |
|  |  |  |  | 549.3 | 2 | FLALVYTDR | Acont_SVSP-8_e14 | SVSP |  |
|  |  |  |  | 595.8 | 2 | IMGWGTISPTK |  |  |  |
|  |  |  |  | 750.9 | 2 | LPATTLCAGILEGGK | Acont_SVSP-17a_e339 | SVSP |  |
|  |  |  |  | 559.8 | 2 | TLCAGILEGGK | Acont_SVSP-3a_e(242,9,339,515) | SVSP | * |
|  | 2.62 | 30▼ |  | 549.3 | 2 | FLALVYTDR | Acont_SVSP-8_e14 | SVSP |  |
|  |  |  |  | 833.1 | 3 | VILPDVPHCANINLLNYSECR |  |  |  |
|  |  |  |  | 830.7 | 3 | FLCGGTLINQEWVLTAAHCDGK |  |  |  |
|  |  |  |  | 595.3 | 2 | WDKDIMLIR | Acont_SVSP-17a_e339 | SVSP |  |
|  |  |  |  | 750.9 | 2 | LPATTLCAGILEGGK |  |  |  |
|  |  |  |  | 403.7 | 2 | KPGLYTK |  |  |  |
|  |  |  |  | 465.3 | 2 | LQFGLHSK | Acont_SVSP-1_e857 | SVSP |  |
|  |  |  |  | 559.8 | 2 | TLCAGILEGGK |  |  |  |
|  |  |  |  | 541.8 | 2 | FLALMFANR | Acont_SVSP-16a_e369 | SVSP |  |
|  | 0.23 | 26▼ |  | 500.2 | 3 | VVGGDECNINEHR | Acont_SVSP-3a_e242 | SVSP |  |
|  |  |  |  | 485.6 | 3 | VLNEDEQIRNPK |  |  |  |
|  |  |  |  | 559.8 | 2 | TLCAGILEGGK |  |  |  |
|  |  |  |  | 750.9 | 2 | LPATTLCAGILEGGK | Acont_SVSP-17a_e339 | SVSP |  |
|  |  |  |  | 490.3 | 3 | LLNEDEQIRNPK | Acont_SVSP-4_e9 | SVSP |  |
|  |  |  |  | 549.3 | 2 | FLALVYTDR | Acont_SVSP-8_e14 | SVSP |  |
|  |  |  |  | 487.2 | 2 | YFCLNTR | Acont_SVSP-18a_e(369,982) | SVSP | * |
| **24** | 0.09 | 45▼ |  | 773.1 | 3 | NSEHIAPLSLPSSPPIVGSVCR | Acont_SVSP-13a_e537 | SVSP |  |
|  |  |  |  | 803.4 | 2 | ILCAGVLEGGIDTCK |  |  |  |
|  |  |  |  | 587.9 | 3 | ILCAGVLEGGIDTCKR |  |  |  |
|  |  |  |  | 559.8 | 2 | TLCAGILEGGK | Acont_SVSP-3a_e(242,9,339,515) | SVSP | * |
|  | 1.68 | 29▼ |  | 401.2 | 3 | LLDKDIMLIK | Acont_SVSP-4_e9 | SVSP |  |
|  |  |  |  | 629.3 | 2 | KLLNEDEQIR |  |  |  |
|  |  |  |  | 532.9 | 3 | KLLNEDEQIRNPK |  |  |  |
|  |  |  |  | 565.3 | 2 | LLNEDEQIR |  |  |  |
|  |  |  |  | 490.3 | 3 | LLNEDEQIRNPK |  |  |  |
|  |  |  |  | 494.3 | 2 | AAYPELPVK | Acont_SVSP-8_e14 | SVSP |  |
|  |  |  |  | 549.3 | 2 | FLALVYTDR |  |  |  |
| **25** | 0.29 | 52▼ |  | 773.1 | 3 | NSEHIAPLSLPSSPPIVGSVCR | Acont_SVSP-13a_e537 | SVSP |  |
|  |  |  |  | 803.4 | 2 | ILCAGVLEGGIDTCK |  |  |  |
|  |  |  |  | 587.9 | 3 | ILCAGVLEGGIDTCKR |  |  |  |
|  |  |  |  | 559.8 | 2 | TLCAGILEGGK | Acont_SVSP-15a_e(824,857,914) | SVSP | * |
|  |  |  |  | 444.7 | 2 | FFCLSSK |  |  |  |
|  |  |  |  | 401.2 | 3 | LLDKDIMLIK | Acont_SVSP-4_e9 | SVSP |  |
|  | 1.65 | 31▼ |  | 419.9 | 3 | KLLNEDEQIR | Acont_SVSP-4_e9 | SVSP |  |
|  |  |  |  | 532.9 | 3 | KLLNEDEQIRNPK |  |  |  |
|  |  |  |  | 565.3 | 2 | LLNEDEQIR |  |  |  |
|  |  |  |  | 490.3 | 3 | LLNEDEQIRNPK |  |  |  |
|  |  |  |  | 401.2 | 3 | LLDKDIMLIK |  |  |  |
|  |  |  |  | 750.9 | 2 | LPATTLCAGILEGGK | Acont_SVSP-17a_e339 | SVSP |  |
|  |  |  |  | 500.2 | 3 | VVGGDECNINEHR | Acont_SVSP-3a_e(9,14,242,339,515) | SVSP | * |
| **26** | 1.09 | 46▼ |  | 405.8 | 3 | KVPNEDEQTR | Acont_SVSP-14a_e824 | SVSP |  |
|  |  |  |  | 741.4 | 2 | AAYPDYELPVTSR |  |  |  |
|  |  |  |  | 559.8 | 2 | TLCAGILEGGK |  |  |  |
|  |  |  |  | 773.1 | 3 | NSEHIAPLSLPSSPPIVGSVCR | Acont_SVSP-13a_e537 | SVSP |  |
|  |  |  |  | 803.4 | 2 | ILCAGVLEGGIDTCK |  |  |  |
|  |  |  |  | 587.9 | 3 | ILCAGVLEGGIDTCKR |  |  |  |
|  | 0.59 | 34▼ |  | 476.9 | 3 | GNMLIFLGVHSLK | Acont_SVSP-7_e420 | SVSP |  |
|  |  |  |  | 559.8 | 2 | TLCAGILEGGK |  |  |  |
|  |  |  |  | 570.8 | 2 | AANPELPATTR |  |  |  |
|  |  |  |  | 403.7 | 2 | FICPNR |  |  |  |
|  |  |  |  | 741.4 | 2 | AAYPDYELPVTSR | Acont_SVSP-14a_e824 | SVSP |  |
|  |  |  |  | 474.7 | 2 | YFFPCSK | Acont_SVSP-19_vtb0097-2 | SVSP |  |
| **28** | 0.17 | 61▼ |  | 555 | 2 | NPLEECFR | Acont_LAAO-1_e495 | LAAO |  |
|  |  |  |  | 562.3 | 2 | HDDIFGYEK |  |  |  |
|  |  |  |  | 441.7 | 2 | IFLTCTK |  |  |  |
|  |  |  |  | 583.3 | 2 | IKFEPPLPPK |  |  |  |
|  |  |  |  | 557.8 | 2 | VIEIQQNDR |  |  |  |
|  | 0.52 | 49▼ |  | 757.8 | 2 | ETDYEEFLEIAR | Acont_LAAO-1_e495 | LAAO |  |
|  |  |  |  | 643.8 | 2 | SAGQLYEESFR |  |  |  |
|  |  |  |  | 562.3 | 2 | HDDIFGYEK |  |  |  |
|  |  |  |  | 557.8 | 2 | VIEIQQNDR |  |  |  |
| **29** | 0.15 | >116▼ |  | 532.7 | 2 | NPLEECFR | Acont_LAAO-1_e495 | LAAO |  |
|  |  |  |  | 643.8 | 2 | SAGQLYEESFR |  |  |  |
|  |  |  |  | 557.8 | 2 | VIEIQQNDR |  |  |  |
|  |  |  |  | 562.3 | 2 | HDDIFGYEK |  |  |  |
|  | 3.61 | 56▼ |  | 532.7 | 2 | NPLEECFR | Acont_LAAO-1_e495 | LAAO |  |
|  |  |  |  | 757.8 | 2 | ETDYEEFLEIAR |  |  |  |
|  |  |  |  | 524.6 | 3 | DPGLLEYPVKPSEK |  |  |  |
|  |  |  |  | 643.8 | 2 | SAGQLYEESFR |  |  |  |
|  |  |  |  | 562.3 | 2 | HDDIFGYEK |  |  |  |
|  |  |  |  | 557.8 | 2 | VIEIQQNDR |  |  |  |
|  |  |  |  | 462.8 | 2 | FEPPLPPK |  |  |  |
|  |  |  |  | 441.7 | 2 | IFLTCTK |  |  |  |
|  | 0.03 | 53▼ |  | 532.7 | 2 | NPLEECFR | Acont_LAAO-1_e495 | LAAO |  |
|  |  |  |  | 757.8 | 2 | ETDYEEFLEIAR |  |  |  |
|  |  |  |  | 524.6 | 3 | DPGLLEYPVKPSEK |  |  |  |
|  |  |  |  | 643.8 | 2 | SAGQLYEESFR |  |  |  |
|  |  |  |  | 562.3 | 2 | HDDIFGYEK |  |  |  |
|  |  |  |  | 557.8 | 2 | VIEIQQNDR |  |  |  |
|  |  |  |  | 441.7 | 2 | IFLTCTK |  |  |  |
|  |  |  |  | 583.3 | 2 | IKFEPPLPPK |  |  |  |
|  | 0.01 | 53▼ |  | 559.3 | 2 | ILNEDEQTR | Acont_SVSP-2a_e957 | SVSP |  |
|  |  |  |  | 403.7 | 2 | FICPNR |  |  |  |
| **30** | 0.12 | 61▼ |  | 757.8 | 2 | ETDYEEFLEIAR | Acont_LAAO-1_e495 | LAAO |  |
|  |  |  |  | 643.8 | 2 | SAGQLYEESFR |  |  |  |
|  |  |  |  | 441.7 | 2 | IFLTCTK |  |  |  |
|  | 0.49 | 53▼ |  | 502.3 | 2 | NMPQCILK | Acont_SVMPIII-6a_e457 | SVMP |  |
|  |  |  |  | 529.8 | 2 | IACEPQNVK |  |  |  |
|  |  |  |  | 684.8 | 2 | LYCFPNSPENK |  |  |  |
| **31** | 0.12 | 60▼ |  | 757.8 | 2 | ETDYEEFLEIAR | Acont_LAAO-1_e495 | LAAO |  |
|  |  |  |  | 524.6 | 3 | DPGLLEYPVKPSEK |  |  |  |
|  |  |  |  | 643.8 | 2 | SAGQLYEESFR |  |  |  |
|  |  |  |  | 557.8 | 2 | VIEIQQNDR |  |  |  |
|  | 1.19 | 53▼ |  | 408.8 | 2 | ATDLLKR | Acont_SVMPIII-6a_e457 | SVMP |  |
|  |  |  |  | 842.8 | 2 | GAQCAEGLCCDQCR |  |  |  |
|  |  |  |  | 571.6 | 3 | AAKDECDMADLCTGR |  |  |  |
|  |  |  |  | 529.8 | 2 | IACEPQNVK |  |  |  |
|  |  |  |  | 684.8 | 2 | LYCFPNSPENK |  |  |  |
|  |  |  |  | 838.0 | 3 | NPCNIYYSPNDEDKGMVLPGTK |  |  |  |
|  | 0.01 | 36▼ |  | 504.9 | 3 | VIGGDECNINEHR | Acont_SVSP-13a_e(369,420,537,857...) | SVSP | * |
|  | 0.01 | 31▼ |  | 571.6 | 3 | AAKDECDMADLCTGR | Acont_SVMPIII-6a_e457 | SVMP |  |
|  |  |  |  | 684.8 | 2 | LYCFPNSPENK |  |  |  |
|  | 0.00 | 31▼ |  | 559.8 | 2 | TLCAGILEGGK | Acont_SVSP-4_e(9,420,515,824...) | SVSP | * |
| **32** | 0.02 | 82▼ |  | 578.6 | 3 | MYDIVNVITPIYHR | Acont_SVMPIII-6a_e457 | SVMP |  |
|  |  |  |  | 502.3 | 2 | NMPQCILK |  |  |  |
|  |  |  |  | 684.8 | 2 | LYCFPNSPENK |  |  |  |
|  | 0.38 | 60▼ |  | 532.7 | 2 | NPLEECFR | Acont_LAAO-1_e495 | LAAO |  |
|  |  |  |  | 757.8 | 2 | ETDYEEFLEIAR |  |  |  |
|  |  |  |  | 643.8 | 2 | SAGQLYEESFR |  |  |  |
|  |  |  |  | 562.3 | 2 | HDDIFGYEK |  |  |  |
|  |  |  |  | 557.8 | 2 | VIEIQQNDR |  |  |  |
|  |  |  |  | 555.8 | 2 | FDEIVGGMDK |  |  |  |
|  | 1.48 | 53▼ |  | 578.6 | 3 | MYDIVNVITPIYHR | Acont_SVMPIII-6a_e457 | SVMP |  |
|  |  |  |  | 502.3 | 2 | NMPQCILK |  |  |  |
|  |  |  |  | 842.8 | 2 | GAQCAEGLCCDQCR |  |  |  |
|  |  |  |  | 571.6 | 3 | AAKDECDMADLCTGR |  |  |  |
|  |  |  |  | 721.8 | 2 | DECDMADLCTGR |  |  |  |
|  |  |  |  | 684.8 | 2 | LYCFPNSPENK |  |  |  |
|  |  |  |  | 401.7 | 2 | GMVLPGTK |  |  |  |
|  | 0.01 | 36▼ |  | 578.6 | 3 | MYDIVNVITPIYHR | Acont_SVMPIII-6a_e457 | SVMP |  |
|  |  |  |  | 842.8 | 2 | GAQCAEGLCCDQCR |  |  |  |
|  |  |  |  | 684.8 | 2 | LYCFPNSPENK |  |  |  |
|  | 0.01 | 36▼ |  | 766.4 | 2 | GLAATTLCAGILEGGK | Acont_SVSP-16a_e(369,914) | SVSP | * |
|  | 0.01 | 31▼ |  | 578.6 | 3 | MYDIVNVITPIYHR | Acont_SVMPIII-6a_e457 | SVMP |  |
|  |  |  |  | 842.8 | 2 | GAQCAEGLCCDQCR |  |  |  |
|  |  |  |  | 684.8 | 2 | LYCFPNSPENK |  |  |  |
|  | 0.01 | 31▼ |  | 500.2 | 3 | VVGGDECNINEHR | Acont_SVSP-3a_e242 | SVSP |  |
|  |  |  |  | 559.8 | 2 | TLCAGILEGGK |  |  |  |
| **33** | 0.52 | 61▼ |  | 591.8 | 2 | LYCFPNSPGK | *A. piscivorus* JAS04447 | SVMP |  |
|  | 0.45 | 60▼ |  | 532.7 | 2 | NPLEECFR | Acont_LAAO-1_e495 | LAAO |  |
|  |  |  |  | 524.6 | 3 | DPGLLEYPVKPSEK |  |  |  |
|  |  |  |  | 643.8 | 2 | SAGQLYEESFR |  |  |  |
|  |  |  |  | 557.8 | 2 | VIEIQQNDR |  |  |  |
|  |  |  |  | 562.3 | 2 | HDDIFGYEK |  |  |  |
|  | 0.02 | 60▼ |  | 521.3 | 2 | IPCAPQDIK | Acont_SVMPIII-3_1f45 | SVMP |  |
|  | 0.03 | 53▼ |  | 643.8 | 2 | SAGQLYEESFR | Acont_LAAO-1_e495 | LAAO |  |
|  |  |  |  | 557.8 | 2 | VIEIQQNDR |  |  |  |
|  |  |  |  | 562.3 | 2 | HDDIFGYEK |  |  |  |
|  | 0.02 | 53▼ |  | 521.3 | 2 | IPCAPQDIK | Acont_SVMPIII-3_1f45 | SVMP |  |
|  |  |  |  | 842.8 | 2 | GAQCAEGLCCDQCR | Acont_SVMPIII-6a_e457 | SVMP |  |
|  |  |  |  | 684.8 | 2 | LYCFPNSPENK |  |  |  |
| **34** | 0.22 | 61▼ |  | 527.7 | 2 | GVCCDQCR | Acont_SVMPIII-3_1f45 | SVMP |  |
|  | 0.09 | 60▼ |  | 532.7 | 2 | NPLEECFR | Acont_LAAO-1_e495 | LAAO |  |
|  |  |  |  | 757.8 | 2 | ETDYEEFLEIAR |  |  |  |
|  |  |  |  | 441.7 | 2 | IFLTCTK |  |  |  |
|  |  |  |  | 524.6 | 3 | DPGLLEYPVKPSEK |  |  |  |
|  |  |  |  | 557.8 | 2 | VIEIQQNDR |  |  |  |
|  | 0.04 | 49▼ |  | 462.8 | 2 | IANGPPVEK | Acont_PLB-1_xng | PLB |  |
|  |  |  |  | 571.3 | 2 | NYQMoxILDTK |  |  |  |
|  | 3.11 | 30▼ |  | 460.7 | 2 | QKDFLNR | Acont_SVMPI-6a_e279 | SVMP |  |
|  |  |  |  | 535.8 | 2 | SVGIVEDHSK |  |  |  |
|  |  |  |  | 437.6 | 3 | SHDNAQLLTAIK |  |  |  |
|  | 0.89 | 25▼ |  | 547.8 | 2 | YNSNLNTIR | Acont_SVMPII-5a_e416 | SVMP |  |
|  |  |  |  | 709.9 | 2 | VSLTDLEVWSNR |  |  |  |
|  |  |  |  | 430.2 | 2 | KTDLLNR | Acont_SVMPI-3a_e522 | SVMP |  |
|  |  |  |  | 723.7 | 3 | SHDNAQLLTAIVFDEGIIGR |  |  |  |
|  |  |  |  | 534.3 | 2 | YNGDSDKIR |  |  |  |
|  | 0.04 | 22▼ |  | 430.2 | 2 | KTDLLNR | Acont_SVMPI-3a_e522 | SVMP |  |
|  | 0.04 | 20▼ |  | 534.3 | 2 | YNGDSDKIR | Acont_SVMPI-6a_e279 | SVMP |  |
|  |  |  |  | 460.7 | 2 | QKDFLNR |  |  |  |
| **35** | 0.24 | 60▼ |  | 532.7 | 2 | NPLEECFR | Acont_LAAO-1_e495 | LAAO |  |
|  |  |  |  | 757.8 | 2 | ETDYEEFLEIAR |  |  |  |
|  |  |  |  | 441.7 | 2 | IFLTCTK |  |  |  |
|  |  |  |  | 524.6 | 3 | DPGLLEYPVKPSEK |  |  |  |
|  |  |  |  | 643.8 | 2 | SAGQLYEESFR |  |  |  |
|  |  |  |  | 557.8 | 2 | VIEIQQNDR |  |  |  |
|  |  |  |  | 562.3 | 2 | HDDIFGYEK |  |  |  |
|  | 2.37 | 30▼ |  | 534.3 | 2 | YNGDSDKIR | Acont_SVMPI-6a_e279 | SVMP |  |
|  |  |  |  | 460.7 | 2 | QKDFLNR |  |  |  |
|  |  |  |  | 437.6 | 3 | SHDNAQLLTAIK |  |  |  |
|  |  |  |  | 535.8 | 2 | SVGIVEDHSK |  |  |  |
|  |  |  |  | 492.7 | 2 | LFSDCSKK |  |  |  |
|  |  |  |  | 430.2 | 2 | QTDLLNR | Acont_SVMPI-2a_e44 | SVMP |  |
|  | 0.06 | 30▼ |  | 559.8 | 2 | TLCAGILEGGK | Acont_SVSP-4_e(9,420,515,824...) | SVSP | * |
|  | 8.51 | 25▼ |  | 657.4 | 2 | YVELVIVADHR | Acont_SVMPI-3a_e522 | SVMP |  |
|  |  |  |  | 430.2 | 2 | KTDLLNR |  |  |  |
|  |  |  |  | 575.1 | 4 | KSHDNAQLLTAIVFDEGIIGR |  |  |  |
|  |  |  |  | 1085.1 | 2 | SHDNAQLLTAIVFDEGIIGR |  |  |  |
|  |  |  |  | 755.7 | 3 | DLSTVTSVSHDTLASFENWR |  |  |  |
|  |  |  |  | 709.9 | 2 | VSLTDLEVWSNR | Acont_SVMPII-5a_e416 | SVMP |  |
|  |  |  |  | 438.2 | 3 | YVQLVIVADHR | Acont_SVMPI-5a_e335 | SVMP |  |
|  | 0.12 | 20▼ |  | 657.4 | 2 | YVELVIVADHR | Acont_SVMPI-3a_e522 | SVMP |  |
|  |  |  |  | 723.7 | 3 | SHDNAQLLTAIVFDEGIIGR |  |  |  |
|  |  |  |  | 755.7 | 3 | DLSTVTSVSHDTLASFENWR |  |  |  |
|  | 0.12 | 14▼ |  | 534.3 | 2 | YNGDSDKIR | Acont_SVMPI-3a_e522 | SVMP |  |
|  |  |  |  | 723.7 | 3 | SHDNAQLLTAIVFDEGIIGR |  |  |  |
|  | 0.36 | 10▼ |  | 534.3 | 2 | YNGDSDKIR | Acont_SVMPI-3a_e522 | SVMP |  |
|  |  |  |  | 723.7 | 3 | SHDNAQLLTAIVFDEGIIGR |  |  |  |
|  |  |  |  | 430.2 | 2 | KTDLLNR |  |  |  |
|  | 0.36 | 6▼ |  | 430.2 | 2 | KTDLLNR | Acont_SVMPI-3a_e522 | SVMP |  |
| **36** | 0.03 | 60▼ |  | 757.8 | 2 | ETDYEEFLEIAR | Acont_LAAO-1_e495 | LAAO |  |
|  |  |  |  | 441.7 | 2 | IFLTCTK |  |  |  |
|  |  |  |  | 562.3 | 2 | HDDIFGYEK |  |  |  |
|  |  |  |  | 643.8 | 2 | SAGQLYEESFR |  |  |  |
|  |  |  |  | 557.8 | 2 | VIEIQQNDR |  |  |  |
|  | 0.03 | 31▼ |  | 461.7 | 2 | GVIGSAYGAK | Acont_SVMPII-2_me463 | SVMP |  |
|  |  |  |  | 406.2 | 3 | FSVGIVQDHSK | Acont_SVMPI-2a_e44 | SVMP |  |
|  | 2.76 | 30▼ |  | 430.2 | 2 | QTDLLNR | Acont_SVMPI-2a_e44 | SVMP |  |
|  |  |  |  | 560.8 | 2 | RGDIGIAYGAK |  |  |  |
|  |  |  |  | 406.2 | 3 | FSVGIVQDHSK |  |  |  |
|  |  |  |  | 492.7 | 2 | LFSDCSKK |  |  |  |
|  |  |  |  | 521.8 | 2 | KDYQTFLK |  |  |  |
|  | 0.09 | 25▼ |  | 406.2 | 3 | FSVGIVQDHSK | Acont_SVMPI-2a_e44 | SVMP |  |
|  |  |  |  | 534.3 | 2 | YNGDSDKIR | Acont_SVMPI-3a_e522 | SVMP |  |
|  |  |  |  | 430.2 | 2 | KTDLLNR |  |  |  |
|  |  |  |  | 626.8 | 2 | MVNTINEIYR | Acont_SVMPI-4a_e575 | SVMP |  |
|  |  |  |  | 719.0 | 3 | SHDNAQLLTAIVFDDGIIGR |  |  |  |
| **37** | 3.44 | 25▼ |  | 626.8 | 2 | MVNTINEIYR | Acont_SVMPI-4a_e575 | SVMP |  |
|  |  |  |  | 601.3 | 2 | APLAGMCDPNR |  |  |  |
|  |  |  |  | 529.3 | 2 | SVGTVQDHSK |  |  |  |
|  |  |  |  | 514.3 | 2 | KDYLTFLK |  |  |  |
|  |  |  |  | 719.0 | 3 | SHDNAQLLTAIVFDDGIIGR |  |  |  |

**Table S4.** Identification by MS/MS of the venom components of *Agkistrodon c. contortrix* specimen M83 (unrelated male) (Fig. 2B). *, ambiguous ID.

| **Spot ID** | **%** | **MW (kDa)** | **ESI-MS (ave)** | **m/z** | **z** | **Peptide sequence** | **Transcriptome match** | **Protein family** | **Observations** |
| --- | --- | --- | --- | --- | --- | --- | --- | --- | --- |
|  |  |  |  |  |  |  |  |  |  |
| **2** | 0.92 |  |  | 592.3 | 2 | ZWPPGHHIPP | Acont_BPP-1a_98 | VAP (BPP) |  |
| **3** | 1.02 |  |  | 532.2 | 2 | TPPAGPDVGPR | Acont_BPP-1a_98 | VAP (BIP) |  |
| **5** | 2.82 |  |  | 430.2 | 1 | ZNW | Acont_BPP-1a_98 | SVMPi |  |
| **6** | 2.19 |  |  | 444.2 | 1 | ZBW | Acont_BPP-1a_98 | SVMPi |  |
| **7** | 0.91 |  |  | 444.2 | 1 | ZBW | Acont_BPP-1a_98 | SVMPi |  |
| **7** |  | 21▪ | 12872,9/  13011,1 | 598.8 | 2 | NPCCDAATCK | Acont_DIS-2a_e351 | Dimeric Disintegrin | Acostatin alpha (Q805F7) |
|  |  |  |  | 992.4 | 2 | LTPGSQCAEGLCCDQCK |  |  | Mave calc (homodimer ^1^ZP..KH^61^/^1^QP..K^60^): 13016,5 |
|  |  |  |  | 569.2 | 2 | CTGQSGDCPR |  |  | Mave calc (homodimer ^1^ZP..K^60^/^1^QP..K^60^): 12879,5 |
|  |  | 18▪ |  | 598.8 | 2 | NPCCDAATCK | Acont_DIS-2a_e351 | Dimeric Disintegrin |  |
|  |  | 8▼ |  | 598.8 | 2 | NPCCDAATCK | Acont_DIS-2a_e351 | Dimeric Disintegrin |  |
|  |  |  |  | 992.4 | 2 | LTPGSQCAEGLCCDQCK | Acont_DIS-2a_e351 | Dimeric Disintegrin |  |
|  |  |  |  | 942.9 | 2 | GDDLDDYCNGISAGCPR | Acont_SVMPII-5a_e416 | Dimeric Disintegrin |  |
| **8** | 1.03 | 14▼ | 13359.3/ 13222.3/ 13085.8 | 992.4 | 2 | LTPGSQCAEGLCCDQCK | Acont_DIS-2a_e351 | Dimeric Disintegrin | C-terminal trimming_ F, H, H |
| **9** | 2.26 | 14▼ | 13506.8 | 992.4 | 2 | LTPGSQCAEGLCCDQCK | Acont_DIS-2a_e351 | Dimeric Disintegrin |  |
| **10** | 0.05 |  |  | 992.4 | 2 | LTPGSQCAEGLCCDQCK | Acont_DIS-2a_e351 | Dimeric Disintegrin |  |
| **15** | 0.13 | 31▼ |  | 569.2 | 2 | CTGQSGDCPR | Acont_PLA2-3a_e604 | PLA2 |  |
|  |  |  |  | 460.3 | 2 | MILQETGK |  |  |  |
|  |  |  |  | 401.7 | 2 | AVAICLR |  |  |  |
|  |  |  |  | 498.7 | 2 | ENLDTYNK |  |  |  |
|  |  |  |  | 562.8 | 2 | ENLDTYNKK |  |  |  |
|  | 12.61 | 14▼ | 14043.1 | 707.3 | 3 | NAITSYGFYGCNCGWGHR | Acont_PLA2-3a_e604 | PLA2 | Mav calc: 14043.3 |
|  |  |  |  | 454.6 | 3 | AIICEEKNPCK |  |  |  |
|  |  |  |  | 401.7 | 2 | AVAICLR |  |  |  |
|  |  |  |  | 498.7 | 2 | ENLDTYNK |  |  |  |
|  | 0.53 | 12▼ |  | 468.2 | 2 | MoxILQETGK | Acont_PLA2-3a_e604 | PLA2 |  |
|  |  |  |  | 454.6 | 3 | (AII)CEEKNPCK |  |  |  |
|  |  |  |  | 401.7 | 2 | AVAICLR |  |  |  |
|  |  |  |  | 562.8 | 2 | ENLDTYNKK |  |  |  |
| **16** | 0.20 | 14▼ | 14043.1 | 707.3 | 3 | NAITSYGFYGCNCGWGHR | Acont_PLA2-3a_e604 | PLA2 |  |
|  |  |  |  | 454.6 | 3 | AIICEEKNPCK |  |  |  |
|  |  |  |  | 401.7 | 2 | AVAICLR |  |  |  |
|  |  |  |  | 498.7 | 2 | ENLDTYNK |  |  |  |
|  |  |  |  | 562.8 | 2 | ENLDTYNKK |  |  |  |
|  | 0.07 | 12▼ |  | 707.3 | 3 | NAITSYGFYGCNCGWGHR | Acont_PLA2-3a_e604 | PLA2 |  |
|  |  |  |  | 454.6 | 3 | AIICEEKNPCK |  |  |  |
|  |  |  |  | 401.7 | 2 | AVAICLR |  |  |  |
|  |  |  |  | 498.7 | 2 | ENLDTYNK |  |  |  |
|  |  |  |  | 562.8 | 2 | ENLDTYNKK |  |  |  |
|  |  |  |  | 468.2 | 2 | MoxILQETGK |  |  |  |
| **18** | 1.52 | 31▼ |  | 1082.1 | 2 | STHIAPLSLPSSPPSLGSVCR | Acont_SVSP-5_e565 | SVSP |  |
|  |  |  |  | 507.8 | 2 | AAYPQLPVR |  |  |  |
|  |  |  |  | 559.8 | 2 | TLCAGILEGGK |  |  |  |
|  | 0.02 | 27▼ |  | 565.3 | 2 | FLVALYTFR | Acont_SVSP-5_e565 | SVSP |  |
|  |  |  |  | 721.7 | 3 | STHIAPLSLPSSPPSLGSVCR |  |  |  |
|  |  |  |  | 507.8 | 2 | AAYPQLPVR |  |  |  |
|  |  |  |  | 559.8 | 2 | TLCAGILEGGK |  |  |  |
|  | 0.29 | 23▼ | 24823.1 | 565.3 | 2 | FLVALYTFR | Acont_SVSP-5_e565 | SVSP |  |
|  |  |  |  | 721.7 | 3 | STHIAPLSLPSSPPSLGSVCR |  |  |  |
|  |  |  |  | 507.8 | 2 | AAYPQLPVR |  |  |  |
|  | 0.06 | 14▼ |  | 551.3 | 2 | LDIYTYSVK | Acont_PLA2-1a_e343 | PLA2 |  |
|  |  |  |  | 445.5 | 2 | DVVCGGTNPCKK |  |  |  |
|  |  |  |  | 404.7 | 2 | AAAICFR |  |  |  |
|  |  |  |  | 707.3 | 3 | NAITSYGFYGCNCGWGHR | Acont_PLA2-3a_e604 | PLA2 |  |
|  |  |  |  | 401.7 | 2 | AVAICLR |  |  |  |
|  | 0.00 | 14▼ |  | 565.3 | 2 | FLVALYTFR | Acont_SVSP-5_e565 | SVSP |  |
|  |  |  |  | 507.8 | 2 | AAYPQLPVR |  |  |  |
|  |  |  |  | 559.8 | 2 | TLCAGILEGGK |  |  |  |
|  | 0.01 | 12▼ |  | 507.8 | 2 | AAYPQLPVR | Acont_SVSP-5_e565 | SVSP |  |
|  |  |  |  | 559.8 | 2 | TLCAGILEGGK |  |  |  |
|  | 0.01 | 12▼ |  | 551.3 | 2 | LDIYTYSVK | Acont_PLA2-1a_e343 | PLA2 |  |
| **19** | 0.32 | 36▼ |  | 716.0 | 3 | SAHIAPLSLPSNPPSVGSVCR | Acont_SVSP-12a_98-10M126 | SVSP |  |
|  |  |  |  | 586.9 | 3 | TLCAGVLEGGIDTCHR |  |  |  |
|  |  |  |  | 727.3 | 2 | AAYPEYDLPATSR | Acont_SVSP-15a_e914 | SVSP |  |
|  |  |  |  | 571.8 | 2 | FLVALYNFR | Acont_SVSP-14a_e824 | SVSP |  |
|  | 0.22 | 33▼ |  | 486.3 | 2 | IYLGVHNR | Acont_SVSP-17a_e339 | SVSP |  |
|  |  |  |  | 750.9 | 2 | LPATTLCAGILEGGK |  |  |  |
|  |  |  |  | 716.0 | 3 | SAHIAPLSLPSNPPSVGSVCR | Acont_SVSP-12a_98-10M126 | SVSP |  |
|  |  |  |  | 565.3 | 2 | FLVALYTFR | Acont_SVSP-5_e565 | SVSP |  |
|  |  |  |  | 507.8 | 2 | AAYPQLPVR |  |  |  |
|  |  |  |  | 559.8 | 2 | TLCAGILEGGK |  |  |  |
|  |  |  |  | 500.2 | 3 | VVGGDECNINEHR | Acont_SVSP-3a_e(242,9,339,515) | SVSP | * |
|  | 0.11 | 23▼ |  | 569.7 | 2 | SVDFDSESPR | *A.piscivorus* JAS04550 | CRISP |  |
|  |  |  |  | 589.3 | 2 | SVNPTASNMEK | *~ A.piscivorus* JAS04550 | CRISP |  |
|  | 0.32 | 16▼ |  | 502.5 | 3 | CCFVHDCCYGK | Acont_PLA2-4_e312 | PLA2 |  |
|  |  |  |  | 491.2 | 2 | EICECDR |  |  |  |
|  |  |  |  | 551.3 | 2 | LDIYTYSVK | Acont_PLA2-1a_e343 | PLA2 |  |
|  | 9.72 | 14▼ | 14163.9 | 781.8 | 2 | CCFVHDCCYNK | Acont_PLA2-1a_e343 | PLA2 | Mave calc: 14164,1 |
|  |  |  |  | 630.0 | 3 | VTSCNPKLDIYTYSVK |  |  |  |
|  |  |  |  | 551.3 | 2 | LDIYTYSVK |  |  |  |
|  |  |  |  | 603.8 | 2 | DVVCGGTNPCK |  |  |  |
|  |  |  |  | 490.7 | 2 | QICECDR |  |  |  |
|  |  |  |  | 404.7 | 2 | AAAICFR |  |  |  |
|  |  |  |  | 607.3 | 2 | DNKDTYDSKK |  |  |  |
|  | 0.04 | 12▼ |  | 760.4 | 2 | NVGVPQVVPDNPER | Acont_Vespryn-1_e296 | Ohanin-like_protein |  |
|  |  |  |  | 763.4 | 2 | FDSSPCVLGSPGFR |  |  |  |
|  |  |  |  | 465.8 | 2 | EWAVGLAGK |  |  |  |
|  |  |  |  | 605.9 | 2 | KGGLLLVPEER |  |  |  |
|  |  |  |  | 541.8 | 2 | GGLLLVPEER |  |  |  |
|  | 0.07 | 12▼ |  | 551.3 | 2 | LDIYTYSVK | Acont_PLA2-1a_e343 | PLA2 |  |
|  |  |  |  | 404.7 | 2 | AAAICFR |  |  |  |
| **21** | 0.07 | 36▼ |  | 405.9 | 3 | KVPNEDEQTR | Acont_SVSP-15a_e914 | SVSP |  |
|  |  |  |  | 727.3 | 2 | AAYPEYDLPATSR |  |  |  |
|  |  |  |  | 559.8 | 2 | TLCAGILEGGK |  |  |  |
|  |  |  |  | 595.3 | 2 | WDKDIMLIR | Acont_SVSP-12a_98-10M126 | SVSP |  |
|  |  |  |  | 716.0 | 3 | SAHIAPLSLPSNPPSVGSVCR |  |  |  |
|  |  |  |  | 519.3 | 2 | AFYPGLLEK |  |  |  |
|  |  |  |  | 571.8 | 2 | FLVALYNFR | Acont_SVSP-14a_e824 | SVSP |  |
|  | 0.59 | 33▼ |  | 486.3 | 2 | IYLGVHNR | Acont_SVSP-11a_e515 | SVSP |  |
|  |  |  |  | 502.5 | 2 | WDKDIMLIR |  |  |  |
|  |  |  |  | 759.4 | 2 | GLAASTLCAGILEGGK |  |  |  |
|  |  |  |  | 750.9 | 2 | LPATTLCAGILEGGK |  |  |  |
|  |  |  |  | 766.4 | 2 | GLAATTLCAGILEGGK | Acont_SVSP-16a_e(369,982) | SVSP | * |
|  |  |  |  | 500.2 | 3 | VVGGDECNINEHR | Acont_SVSP-3a_e(242,9,339,515) | SVSP | * |
|  |  |  |  | 559.8 | 2 | TLCAGILEGGK |  |  |  |
|  |  |  |  | 507.8 | 2 | AAYPQLPVR | Acont_SVSP-5_e565 | SVSP |  |
|  |  |  |  | 405.8 | 3 | KVPNEDEQTR | Acont_SVSP-14a_e(824,914) | SVSP | * |
|  | 0.63 | 14▼ | 14163.9 | 630.0 | 3 | VTSCNPKLDIYTYSVK | Acont_PLA2-1a_e343 | PLA2 |  |
|  |  |  |  | 551.3 | 2 | LDIYTYSVK |  |  |  |
|  |  |  |  | 603.8 | 2 | DVVCGGTNPCK |  |  |  |
|  | 0.02 | 14▼ |  | 621.3 | 2 | DFSWEWTDR | Acont_CTL-9a_e4 | CTL |  |
|  |  |  |  | 786.9 | 2 | EFCVELVSLTGYR |  |  |  |
|  | 0.01 | 14▼ |  | 486.3 | 2 | IYLGVHNR | Acont_SVSP-11a_e(515,339,369) | SVSP | * |
| **22a** | 0.63 | 36▼ |  | 544.3 | 2 | VPNEDEQTR | Acont_SVSP-15a_e914 | SVSP |  |
|  |  |  |  | 573.3 | 2 | EKFFCLSSK |  |  |  |
|  |  |  |  | 727.3 | 2 | AAYPEYDLPATSR |  |  |  |
|  |  |  |  | 559.8 | 2 | TLCAGILEGGK |  |  |  |
|  |  |  |  | 550.9 | 3 | TLCAGILEGGKDTCR |  |  |  |
|  |  |  |  | 571.8 | 2 | FLVALYNFR | Acont_SVSP-14a_e824 | SVSP |  |
|  | 2.36 | 31▼ |  | 654.3 | 2 | NFQMLFGVHSK | Acont_SVSP-2a_e957 | SVSP |  |
|  |  |  |  | 559.3 | 2 | ILNEDEQTR |  |  |  |
|  |  |  |  | 497.6 | 3 | DDEKDKDIMLIR |  |  |  |
|  |  |  |  | 604.8 | 2 | IMoxGWGTTTPTK |  |  |  |
|  |  |  |  | 415.9 | 3 | KILNEDEQTR |  |  |  |
|  |  |  |  | 502.3 | 2 | NMPQCILK |  |  |  |
|  |  |  |  | 1126.6 | 2 | AAYPELLAESSTLCAGTQQGGK |  |  |  |
|  | 0.03 | 17▼ |  | 654.3 | 2 | NFQMLFGVHSK | Acont_SVSP-2a_e957 | SVSP |  |
|  |  |  |  | 559.3 | 2 | ILNEDEQTR |  |  |  |
|  |  |  |  | 604.8 | 2 | IMoxGWGTTTPTK |  |  |  |
|  |  |  |  | 403.7 | 2 | FICPNR |  |  |  |
|  | 0.09 | 16▼ |  | 502.5 | 3 | CCFVHDCCYGK | Acont_PLA2-4_e312 | PLA2 |  |
|  |  |  |  | 491.2 | 2 | EICECDR |  |  |  |
|  |  |  |  | 404.7 | 2 | AAAICFR |  |  |  |
|  | 0.00 | 16▼ |  | 403.7 | 2 | FICPNR | Acont_SVSP-2a_e(957,420) | SVSP | * |
|  | 0.03 | 14▼ | 13753.4 | 490.7 | 2 | QICECDR | Acont_PLA2-1a_e343 | PLA2 | Mave calc: 13753,4 |
|  |  |  |  | 404.7 | 2 | AAAICFR |  |  |  |
|  |  |  |  | 502.5 | 3 | (CC)FVHDCCYGK | Acont_PLA2-4_e312 | PLA2 |  |
|  |  |  |  | 491.2 | 2 | EICECDR |  |  |  |
|  | 0.00 | 14▼ |  | 644.8 | 2 | SCTDYLTWDK | Acont_CTL-9a_e4 | CTL |  |
| **22** | 2.78 | 36▼ |  | 486.3 | 2 | IYLGVHNR | Acont_SVSP-11a_e515 | SVSP |  |
|  |  |  |  | 754.1 | 3 | NSAHIAPLSLPSNPPSVGSVCR |  |  |  |
|  |  |  |  | 766.4 | 2 | GLAATTLCAGILEGGK |  |  |  |
|  |  |  |  | 750.7 | 3 | NSAHIAPLSLPSNSPSVGSVCR | Acont_SVSP-18a_e982 | SVSP |  |
|  |  |  |  | 571.8 | 2 | FLVALYNFR | Acont_SVSP-14a_e824 | SVSP |  |
|  |  |  |  | 544.3 | 2 | VPNEDEQTR |  |  |  |
|  |  |  |  | 559.8 | 2 | TLCAGILEGGK |  |  |  |
|  |  |  |  | 550.9 | 3 | TLCAGILEGGKDTCR |  |  |  |
|  |  |  |  | 727.3 | 2 | AAYPEYDLPATSR | Acont_SVSP-15a_e914 | SVSP |  |
|  | 0.25 | 36▼ |  | 491.2 | 2 | EICECDR | Acont_PLA2-4_e312 | PLA2 |  |
|  |  |  |  | 404.7 | 2 | AAAICFR |  |  |  |
|  | 2.40 | 31▼ |  | 559.3 | 2 | ILNEDEQTR | Acont_SVSP-2a_e957 | SVSP |  |
|  |  |  |  | 596.8 | 2 | IMGWGTTTPTK |  |  |  |
|  |  |  |  | 403.7 | 2 | FICPNR |  |  |  |
|  |  |  |  | 559.8 | 2 | TLCAGILEGGK | Acont_SVSP-3a_e(242,9,339,515) | SVSP | * |
|  |  |  |  | 766.4 | 2 | GLAATTLCAGILEGGK | Acont_SVSP-18a_e(982,369) | SVSP | * |
|  | 10.07 | 14▼ | 13753.4 | 502.5 | 3 | CCFVHDCCYGK | Acont_PLA2-4_e312 | PLA2 |  |
|  |  |  |  | 539.9 | 3 | DNKDTYDNKYWR |  |  |  |
|  |  |  |  | 630.8 | 2 | DTYDNKYWR |  |  |  |
|  | 0.32 | 8▼ |  | 491.2 | 2 | EICECDR | Acont_PLA2-4_e312 | PLA2 |  |
|  |  |  |  | 404.7 | 2 | AAAICFR |  |  |  |
|  | 0.16 | 4▼ |  | 491.2 | 2 | EICECDR | Acont_PLA2-4_e312 | PLA2 |  |
|  |  |  |  | 502.5 | 3 | CCFVHDCCYGK |  |  |  |
| **23** | 0.01 | \| 40 \| \| --- \| |  | 803.4 | 2 | ILCAGVLEGGIDTCK | Acont_SVSP-13a_e537 | SVSP |  |
|  |  |  |  | 750.9 | 2 | LPATTLCAGILEGGK |  |  |  |
|  |  |  |  | 766.4 | 2 | GLAATTLCAGILEGGK | Acont_SVSP-18a_e(982,369) | SVSP | * |
|  |  |  |  | 541.8 | 2 | GGLLLVPEER | Acont_Vespryn-1_e296 | Ohanin-like |  |
|  |  |  |  | 500.2 | 3 | VVGGDECNINEHR | Acont_SVSP-3a_e(242,9,339,515) | SVSP | * |
|  |  |  |  | 559.8 | 2 | TLCAGILEGGK | Acont_SVSP-3a_e(242,9,339,515..) | SVSP | * |
|  | 0.02 | \| 26 \| \| --- \| |  | 565.3 | 2 | LLNEDEQIR | Acont_SVSP-4_e9 | SVSP |  |
|  | 0.60 | \| 23 \| \| --- \| |  | 549.3 | 2 | FLALVYTDR | Acont_SVSP-8_e14 | SVSP |  |
|  |  |  |  | 595.8 | 2 | IMGWGTISPTK |  |  |  |
|  |  |  |  | 494.3 | 2 | AAYPELPVK |  |  |  |
|  |  |  |  | 558.3 | 2 | VLNEDEQIR |  |  |  |
|  | 0.11 | \| 21 \| \| --- \| |  | 549.3 | 2 | FLALVYTDR | Acont_SVSP-8_e14 | SVSP |  |
|  |  |  |  | 595.8 | 2 | IMGWGTISPTK |  |  |  |
|  |  |  |  | 494.3 | 2 | AAYPELPVK |  |  |  |
|  |  |  |  | 558.3 | 2 | VLNEDEQIR |  |  |  |
|  |  |  |  | 532.9 | 3 | KLLNEDEQIRNPK | Acont_SVSP-4_e9 | SVSP |  |
|  |  |  |  | 565.3 | 2 | LLNEDEQIR |  |  |  |
|  |  |  |  | 500.2 | 3 | VVGGDECNINEHR | Acont_SVSP-3a_e(242,9,339,515) | SVSP | * |
|  |  |  |  | 485.6 | 3 | VLNEDEQIRNPK | Acont_SVSP-3a_e242 | SVSP |  |
|  | 0.02 | \| 19 \| \| --- \| |  | 500.2 | 3 | VVGGDECNINEHR | Acont_SVSP-3a_e(242,9,339,515) | SVSP | * |
|  |  |  |  | 559.8 | 2 | TLCAGILEGGK | Acont_SVSP-3a_e(242,9,339,515..) | SVSP | * |
|  |  |  |  | 558.3 | 2 | VLNEDEQIR | Acont_SVSP-3a_e242 | SVSP |  |
|  |  |  |  | 485.6 | 3 | VLNEDEQIRNPK | Acont_SVSP-3a_e242 | SVSP |  |
|  |  |  |  | 559.8 | 2 | TLCAGILEGGK | Acont_SVSP-3a_e(242,9,339,515..) | SVSP | * |
| **24** | 0.20 | 53▼ |  | 587.9 | 3 | ILCAGVLEGGIDTCKR | Acont_SVSP-13a_e537 | SVSP |  |
|  |  |  |  | 565.3 | 2 | LLNEDEQIR | Acont_SVSP-4_e9 | SVSP |  |
|  | 0.05 | 51▼ |  | 544.3 | 2 | VPNEDEQTR | Acont_SVSP-14a_e824 | SVSP |  |
|  |  |  |  | 741.4 | 2 | AAYPDYELPVTSR |  |  |  |
|  |  |  |  | 559.8 | 2 | TLCAGILEGGK |  |  |  |
|  | 2.26 | 31▼ |  | 532.9 | 3 | KLLNEDEQIRNPK | Acont_SVSP-4_e9 | SVSP |  |
|  |  |  |  | 490.3 | 3 | LLNEDEQIRNPK |  |  |  |
|  |  |  |  | 401.2 | 3 | LLDKDIMLIK |  |  |  |
|  |  |  |  | 559.8 | 2 | TLCAGILEGGK | Acont_SVSP-3a_e(242,9,339,515..) | SVSP | * |
|  |  |  |  | 500.2 | 3 | VVGGDECNINEHR | Acont_SVSP-3a_e(242,9,339,515) | SVSP | * |
| **25** | 0.19 | 53▼ |  | 773.1 | 3 | NSEHIAPLSLPSSPPIVGSVCR | Acont_SVSP-13a_e537 | SVSP |  |
|  |  |  |  | 803.4 | 2 | ILCAGVLEGGIDTCK |  |  |  |
|  |  |  |  | 587.9 | 3 | ILCAGVLEGGIDTCKR |  |  |  |
|  |  |  |  | 739.7 | 3 | DSGGPLICNGQFQGIVSWGPK |  |  |  |
|  |  |  |  | 405.8 | 3 | KVPNEDEQTR | Acont_SVSP-14a_e824 | SVSP |  |
|  |  |  |  | 741.4 | 2 | AAYPDYELPVTSR |  |  |  |
|  |  |  |  | 559.8 | 2 | TLCAGILEGGK | Acont_SVSP-3a_e(242,9,339,515..) | SVSP | * |
|  | 0.56 | 51▼ |  | 741.4 | 2 | AAYPDYELPVTSR | Acont_SVSP-14a_e824 | SVSP |  |
|  |  |  |  | 559.8 | 2 | TLCAGILEGGK | Acont_SVSP-3a_e(242,9,339,515..) | SVSP | * |
|  |  |  |  | 550.9 | 3 | TLCAGILEGGKDTCR | Acont_SVSP-14a_e(824,914) | SVSP | * |
|  |  |  |  | 803.4 | 2 | ILCAGVLEGGIDTCK | Acont_SVSP-13a_e537 | SVSP |  |
|  | 1.11 | 31▼ |  | 629.3 | 2 | KLLNEDEQIR | Acont_SVSP-4_e9 | SVSP |  |
|  |  |  |  | 532.9 | 3 | KLLNEDEQIRNPK |  |  |  |
|  |  |  |  | 565.3 | 2 | LLNEDEQIR |  |  |  |
|  |  |  |  | 401.2 | 3 | LLDKDIMLIK |  |  |  |
|  |  |  |  | 490.3 | 3 | LLNEDEQIRNPK |  |  |  |
|  |  |  |  | 559.8 | 2 | TLCAGILEGGK |  |  |  |
|  |  |  |  | 500.2 | 3 | VVGGDECNINEHR | Acont_SVSP-3a_e(242,9,339,515) | SVSP | * |
|  |  |  |  | 750.9 | 2 | LPATTLCAGILEGGK | Acont_SVSP-17a_e339 | SVSP |  |
| **26** | 1.83 | 51▼ |  | 741.4 | 2 | AAYPDYELPVTSR | Acont_SVSP-14a_e824 | SVSP |  |
|  |  |  |  | 559.8 | 2 | TLCAGILEGGK | Acont_SVSP-3a_e(242,9,339,515..) | SVSP | * |
|  | 2.06 | 33▼ |  | 476.9 | 3 | GNMLIFLGVHSLK | Acont_SVSP-7_e420 | SVSP |  |
|  |  |  |  | 497.6 | 3 | DDEKDKDIMLIR |  |  |  |
|  |  |  |  | 502.3 | 2 | DKDIMLIR |  |  |  |
|  |  |  |  | 570.8 | 2 | AANPELPATTR |  |  |  |
|  |  |  |  | 559.8 | 2 | TLCAGILEGGK |  |  |  |
|  |  |  |  | 544.3 | 2 | VPNEDEQTR | Acont_SVSP-14a_e824 | SVSP |  |
|  |  |  |  | 741.4 | 2 | AAYPDYELPVTSR |  |  |  |
|  |  |  |  | 766.4 | 2 | GLAATTLCAGILEGGK | Acont_SVSP-16a_e369 | SVSP |  |
|  |  |  |  | 467.9 | 3 | FLVTLHDAWSGR | Acont_SVSP-19_vtb0097-2 | SVSP |  |
|  |  |  |  | 474.7 | 2 | YFFPCSK |  |  |  |
| **28** | 0.07 | 55▼ |  | 557.8 | 2 | VIEIQQNDR | Acont_LAAO-1_e495 | LAAO |  |
|  |  |  |  | 438.7 | 2 | STTDLPSR |  |  |  |
|  | 0.03 | 55▼ |  | 476.9 | 3 | GNMLIFLGVHSLK | Acont_SVSP-7_e420 | SVSP |  |
|  |  |  |  | 570.8 | 2 | AANPELPATTR |  |  |  |
|  |  |  |  | 559.8 | 2 | TLCAGILEGGK |  |  |  |
|  |  |  |  | 500.2 | 3 | VVGGDECNINEHR | Acont_SVSP-3a_e(242,9,339,515) | SVSP | * |
|  |  |  |  | 741.4 | 2 | AAYPDYELPVTSR | Acont_SVSP-14a_e824 | SVSP |  |
|  | 0.90 | 36▼ |  | 757.8 | 2 | ETDYEEFLEIAR | Acont_LAAO-1_e495 | LAAO |  |
|  |  |  |  | 524.6 | 3 | (DPGLLE)YPVKPSEK |  |  |  |
|  |  |  |  | 643.8 | 2 | SAGQLYEESFR |  |  |  |
|  |  |  |  | 562.3 | 2 | HDDIFGYEK |  |  |  |
|  |  |  |  | 557.8 | 2 | VIEIQQNDR |  |  |  |
|  |  |  |  | 583.4 | 2 | IKFEPPLPPK |  |  |  |
|  |  |  |  | 555.8 | 2 | FDEIVGGMDK |  |  |  |
| **29** | 0.31 | 55▼ |  | 757.8 | 2 | ETDYEEFLEIAR | Acont_LAAO-1_e495 | LAAO |  |
|  |  |  |  | 524.6 | 3 | (DPGLLE)YPVKPSEK |  |  |  |
|  |  |  |  | 643.8 | 2 | SAGQLYEESFR |  |  |  |
|  |  |  |  | 557.8 | 2 | VIEIQQNDR |  |  |  |
|  |  |  |  | 583.4 | 2 | IKFEPPLPPK |  |  |  |
|  | 0.16 | 55▼ |  | 529.8 | 2 | IACEPQNVK | Acont_SVMPIII-6a_e457 | SVMP |  |
|  | 1.67 | 51▼ |  | 578.6 | 3 | MYDIVNVITPIYHR | Acont_SVMPIII-6a_e457 | SVMP |  |
|  |  |  |  | 502.3 | 2 | NMPQCILK |  |  |  |
|  |  |  |  | 571.6 | 3 | AAKDECDMADLCTGR |  |  |  |
|  |  |  |  | 684.8 | 2 | LYCFPNSPENK |  |  |  |
|  |  |  |  | 729.8 | 2 | DECDMADLCTGR |  |  |  |
|  |  |  |  | 401.7 | 2 | AVAICLR |  |  |  |
|  | 0.06 | 33▼ |  | 842.8 | 2 | GAQCAEGLCCDQCR | Acont_SVMPIII-6a_e457 | SVMP |  |
|  |  |  |  | 684.8 | 2 | LYCFPNSPENK |  |  |  |
|  |  |  |  | 729.8 | 2 | DECDMADLCTGR |  |  |  |
|  | 0.08 | 33▼ |  | 487.2 | 2 | YFCLNTR | Acont_SVSP-18a_e(982,369) | SVSP | * |
|  |  |  |  | 766.4 | 2 | GLAATTLCAGILEGGK |  |  |  |
|  |  |  |  | 559.8 | 2 | TLCAGILEGGK | Acont_SVSP-3a_e(242,9,339,515..) | SVSP | * |
|  | 0.05 | 21▼ |  | 532.7 | 2 | NPLEECFR | Acont_LAAO-1_e495 | LAAO |  |
| **31** | 0.50 | 55▼ |  | 757.8 | 2 | ETDYEEFLEIAR | Acont_LAAO-1_e495 | LAAO |  |
|  |  |  |  | 643.8 | 2 | SAGQLYEESFR |  |  |  |
|  |  |  |  | 562.3 | 2 | HDDIFGYEK |  |  |  |
|  |  |  |  | 557.8 | 2 | VIEIQQNDR |  |  |  |
|  |  |  |  | 430.2 | 3 | FWEEEGIHGGK |  |  |  |
|  | 0.68 | 51▼ |  | 502.3 | 2 | NMPQCILK | Acont_SVMPIII-6a_e457 | SVMP |  |
|  |  |  |  | 842.8 | 2 | GAQCAEGLCCDQCR |  |  |  |
|  |  |  |  | 684.8 | 2 | LYCFPNSPENK |  |  |  |
|  | 0.07 | 51▼ |  | 524.6 | 3 | (DPGLLE)YPVKPSEK | Acont_LAAO-1_e495 | LAAO |  |
|  |  |  |  | 562.3 | 2 | HDDIFGYEK |  |  |  |
| **32** | 0.92 | 55▼ |  | 587.3 | 2 | IFPCAPQNVK | Acont_SVMPIII-3_1f45 | SVMP |  |
|  |  |  |  | 850.4 | 2 | NQCIYFFGPNAAVAK |  |  |  |
|  |  |  |  | 521.3 | 2 | IPCAPQDIK |  |  |  |
|  | 0.55 | 53▼ |  | 532.7 | 2 | NPLEECFR | Acont_LAAO-1_e495 | LAAO |  |
|  |  |  |  | 757.8 | 2 | ETDYEEFLEIAR |  |  |  |
|  |  |  |  | 643.8 | 2 | SAGQLYEESFR |  |  |  |
|  |  |  |  | 562.3 | 2 | HDDIFGYEK |  |  |  |
|  |  |  |  | 557.8 | 2 | VIEIQQNDR |  |  |  |
|  |  |  |  | 430.2 | 3 | FWEEEGIHGGK |  |  |  |
|  |  |  |  | 524.6 | 3 | (DPGLLE)YPVKPSEK |  |  |  |
|  |  |  |  | 555.8 | 2 | FDEIVGGMDK |  |  |  |
|  |  |  |  | 441.7 | 2 | IFLTCTK |  |  |  |
|  | 0.04 | 53▼ |  | 742.6 | 3 | LHSWVECESGECCEQCR | Acont_SVMPIII-2_me561 | SVMP |  |
|  |  |  |  | 752.3 | 2 | LFCEFNNFPCR |  |  |  |
|  |  |  |  | 658.3 | 2 | YSDDGMVDQGTK |  |  |  |
|  |  |  |  | 578.6 | 3 | MYDIVNVITPIYHR | Acont_SVMPIII-6a_e457 | SVMP |  |
|  | 0.12 | 51▼ |  | 666.8 | 2 | VVPESLFAWER | Acont_PLB-1_xng | PLB |  |
|  |  |  |  | 470.7 | 2 | VTDMESMK |  |  |  |
|  |  |  |  | 453.2 | 2 | VADISMAAK |  |  |  |
|  |  |  |  | 703.9 | 2 | FTAYAINGPPVEK |  |  |  |
|  | 0.02 | 51▼ |  | 562.3 | 2 | HDDIFGYEK | Acont_LAAO-1_e495 | LAAO |  |
|  |  |  |  | 557.8 | 2 | VIEIQQNDR |  |  |  |
|  | 0.01 | 26▼ |  | 460.7 | 2 | QKDFLNR | Acont_SVMPI-6a_e279 | PI-SVMP |  |
|  | 0.00 | 26▼ |  | 557.8 | 2 | VIEIQQNDR | Acont_LAAO-1_e495 | LAAO |  |
|  | 0.02 | 23▼ |  | 850.4 | 2 | NQCIYFFGPNAAVAK | Acont_SVMPIII-3_1f45 | SVMP |  |
|  |  |  |  | 521.3 | 2 | IPCAPQDIK |  |  |  |
|  |  |  |  | 430.3 | 2 | KTDLLNR |  |  |  |
| **34** | 0.41 | 55▼ |  | 590.3 | 2 | LYCFPNSPGK | *A. piscivorus* JAS04447 | SVMP |  |
|  | 0.45 | 53▼ |  | 757.8 | 2 | ETDYEEFLEIAR | Acont_LAAO-1_e495 | LAAO |  |
|  |  |  |  | 643.8 | 2 | SAGQLYEESFR |  |  |  |
|  |  |  |  | 555.8 | 2 | FDEIVGGMDK |  |  |  |
|  |  |  |  | 557.8 | 2 | VIEIQQNDR |  |  |  |
|  |  |  |  | 441.7 | 2 | IFLTCTK |  |  |  |
|  |  |  |  | 472.9 | 3 | KFWEEEGIHGGK |  |  |  |
|  |  |  |  | 438.7 | 2 | STTDLPSR |  |  |  |
|  | 0.14 | 53▼ |  | 752.3 | 2 | LFCEFNNFPCR | Acont_SVMPIII-2_me561 | SVMP |  |
|  |  |  |  | 658.3 | 2 | YSDDGMVDQGTK |  |  |  |
|  | 0.06 | 51▼ |  | 453.2 | 2 | (K)VADISMAAK(F) | Acont_PLB-1_xng | PLB |  |
|  |  |  |  | 506.2 | 2 | TWAETFEK |  |  |  |
|  | 3.66 | 26▼ |  | 657.4 | 2 | YVELVIVADHR | Acont_SVMPI-6a_e279 | SVMP |  |
|  |  |  |  | 534.3 | 2 | YNGDSDKIR |  |  |  |
|  |  |  |  | 655.9 | 2 | SHDNAQLLTAIK |  |  |  |
|  |  |  |  | 535.8 | 2 | SVGIVEDHSK |  |  |  |
|  |  |  |  | 649.3 | 3 | HDGNQCHCGANSCIMGK |  |  |  |
|  |  |  |  | 983.5 | 2 | LNRPTIGIAYTAGMoxCSPK |  |  |  |
|  |  |  |  | 548.1 | 4 | INLLVAVTMAHELGHNLGMR |  |  |  |
|  |  |  |  | 648.9 | 5 | SVGIVEDHSKINLLVAVTMAHELGHNLGMR |  |  |  |
|  |  |  |  | 437.7 | 2 | TLSNQPSK |  |  |  |
|  |  |  |  | 579.3 | 3 | VAVTMAHELGHNLGMR |  |  |  |
|  | 1.18 | 23▼ |  | 547.8 | 2 | YNSNLNTIR | Acont_SVMPII-5a_e416 | SVMP |  |
|  |  |  |  | 709.9 | 2 | VSLTDLEVWSNR |  |  |  |
|  |  |  |  | 430.3 | 2 | KTDLLNR | Acont_SVMPI-3a_e(522,98-1M61) | SVMP | * |
| **35** | 0.11 | 53▼ |  | 532.7 | 2 | NPLEECFR | Acont_LAAO-1_e495 | LAAO |  |
|  |  |  |  | 757.8 | 2 | ETDYEEFLEIAR |  |  |  |
|  |  |  |  | 643.8 | 2 | SAGQLYEESFR |  |  |  |
|  |  |  |  | 562.3 | 2 | HDDIFGYEK |  |  |  |
|  |  |  |  | 557.8 | 2 | VIEIQQNDR |  |  |  |
|  |  |  |  | 583.4 | 2 | IKFEPPLPPK |  |  |  |
|  |  |  |  | 472.9 | 3 | KFWEEEGIHGGK |  |  |  |
|  | 0.01 | 53▼ |  | 430.3 | 2 | KTDLLNR | Acont_SVMPI-3a_e(522,98-1M61) | SVMP | * |
|  | 0.23 | \| 51 \| \| --- \| |  | 664.4 | 2 | YIELVIVADHR | Acont_SVMPII-6a_e592 | SVMP |  |
|  |  |  |  | 753.3 | 2 | AYQECMCFPQR |  |  |  |
|  |  |  |  | 935.8 | 2 | GDDVDDYCNGISAGCPR |  |  |  |
|  |  |  |  | 534.3 | 2 | YNGDSDKIR | Acont_SVMPI-6a_e279 | SVMP |  |
|  | 1.74 | 29▼ |  | 460.7 | 2 | QKDFLNR |  |  |  |
|  |  |  |  | 535.8 | 2 | SVGIVEDHSK |  |  |  |
|  |  |  |  | 430.2 | 2 | QTDLLNR | Acont_SVMPI-2a_e44 | SVMP |  |
|  |  |  |  | 560.8 | 2 | RGDIGIAYGAK |  |  |  |
|  |  |  |  | 406.2 | 3 | FSVGIVQDHSK |  |  |  |
|  | 8.68 | 26▼ |  | 438.6 | 3 | YVELVIVADHR | Acont_SVMPI-3a_e522 | SVMP |  |
|  |  |  |  | 534.3 | 2 | YNGDSDKIR |  |  |  |
|  |  |  |  | 430.3 | 2 | KTDLLNR |  |  |  |
|  |  |  |  | 723.7 | 3 | SHDNAQLLTAIVFDEGIIGR |  |  |  |
|  |  |  |  | 1133.0 | 2 | DLSTVTSVSHDTLASFENWR |  |  |  |
|  |  |  |  | 709.9 | 2 | VSLTDLEVWSNR | Acont_SVMPII-5a_e416 | SVMP |  |
|  | 0.12 | 21▼ |  | 430.3 | 2 | KTDLLNR | Acont_SVMPI-3a_e(522,98-1M61) | SVMP | * |
|  |  |  |  | 723.7 | 3 | SHDNAQLLTAIVFDEGIIGR | Acont_SVMPI-3a_e522 | SVMP |  |
|  | 0.12 | 14▼ |  | 534.3 | 2 | YNGDSDKIR |  |  |  |
|  |  |  |  | 430.3 | 2 | KTDLLNR | Acont_SVMPI-3a_e(522,98-1M61) | SVMP |  |
|  |  |  |  | 723.7 | 3 | SHDNAQLLTAIVFDEGIIGR | Acont_SVMPI-3a_e522 | SVMP |  |
|  | 0.35 | 10▼ |  | 534.3 | 2 | YNGDSDKIR |  |  |  |
|  |  |  |  | 430.3 | 2 | KTDLLNR | Acont_SVMPI-3a_e(522,98-1M61) | SVMP |  |
|  |  |  |  | 723.7 | 3 | SHDNAQLLTAIVFDEGIIGR | Acont_SVMPI-3a_e522 | SVMP |  |
|  |  |  |  | 755.7 | 3 | DLSTVTSVSHDTLASFENWR |  |  |  |
|  |  |  |  | 626.8 | 2 | MVNTINEIYR | Acont_SVMPI-4a_e(575,522) | SVMP | * |
|  | 0.23 | 8▼ |  | 657.8 | 2 | YVELVIVADHR | Acont_SVMPI-3a_e522 | SVMP |  |
|  |  |  |  | 723.7 | 3 | SHDNAQLLTAIVFDEGIIGR |  |  |  |
|  |  |  |  | 492.7 | 2 | LFSDCSKK |  |  |  |
| **35a** | 0.38 | 53▼ |  | 532.7 | 2 | NPLEECFR | Acont_LAAO-1_e495 | LAAO |  |
|  |  |  |  | 757.8 | 2 | ETDYEEFLEIAR |  |  |  |
|  |  |  |  | 524.6 | 3 | (DPGLL)EYPVKPSEK |  |  |  |
|  |  |  |  | 643.8 | 2 | SAGQLYEESFR |  |  |  |
|  |  |  |  | 562.3 | 2 | HDDIFGYEK |  |  |  |
|  |  |  |  | 557.8 | 2 | VIEIQQNDR |  |  |  |
|  |  |  |  | 583.4 | 2 | IKFEPPLPPK |  |  |  |
|  |  |  |  | 472.9 | 3 | KFWEEEGIHGGK |  |  |  |
|  |  |  |  | 571.3 | 3 | DCGDIVINDLSLIHK |  |  |  |
|  | 0.20 | 51▼ |  | 757.8 | 2 | ETDYEEFLEIAR | Acont_LAAO-1_e495 | LAAO |  |
|  |  |  |  | 524.6 | 3 | DPGLLEYPVKPSEK |  |  |  |
|  |  |  |  | 643.8 | 2 | SAGQLYEESFR |  |  |  |
|  |  |  |  | 562.3 | 2 | HDDIFGYEK |  |  |  |
|  |  |  |  | 557.8 | 2 | VIEIQQNDR |  |  |  |
|  |  |  |  | 472.9 | 3 | KFWEEEGIHGGK |  |  |  |
|  |  |  |  | 571.3 | 3 | DCGDIVINDLSLIHK |  |  |  |
|  |  |  |  | 438.7 | 2 | STTDLPSR |  |  |  |
|  | 0.52 | 51▼ |  | 806.9 | 2 | IYACVNTLNLIYR | Acont_SVMPIII-7_98-5M50 | PIII-SVMP |  |
|  |  |  |  | 541.8 | 2 | SAGIIQDHNK |  |  |  |
|  |  |  |  | 844.3 | 3 | LRPGTQCEDGECCEQCQFTR |  |  |  |
|  |  |  |  | 538.3 | 2 | IFPCAPQDK |  |  |  |
|  |  |  |  | 457.7 | 2 | DYQTFLK | Acont_SVMPI-2a_e44 | SVMP |  |
|  | 0.07 | 33▼ |  | 461.7 | 2 | GVIGSAYGAK | Acont_SVMPII-2_me463 | PII-SVMP |  |
|  | 0.03 | 33▼ |  | 487.2 | 2 | YFCLNTR | Acont_SVSP-18a_e982 | SVSP |  |
|  |  |  |  | 766.4 | 2 | GLAATTLCAGILEGGK |  |  |  |
|  |  |  |  | 570.8 | 2 | AANPELPATTR | Acont_SVSP-7_e420 | SVSP |  |
|  |  |  |  | 559.8 | 2 | TLCAGILEGGK |  |  |  |
|  |  |  |  | 482.7 | 2 | GDIGIAYGAK | Acont_SVMPI-2a_e44 | SVMP |  |
|  |  |  |  | 759.4 | 2 | GLAASTLCAGILEGGK | Acont_SVSP-11a_e515 | SVSP |  |
|  | 1.98 | 26▼ |  | 657.4 | 2 | YVELVIVADHR | Acont_SVMPI-2a_e44 | SVMP |  |
|  |  |  |  | 430.2 | 2 | QTDLLNR |  |  |  |
|  |  |  |  | 560.8 | 2 | RGDIGIAYGAK |  |  |  |
|  |  |  |  | 406.2 | 3 | FSVGIVQDHSK |  |  |  |
|  |  |  |  | 521.8 | 2 | KDYQTFLK |  |  |  |
|  |  |  |  | 657.4 | 2 | YVELVIVADHR | Acont_SVMPII-1_me196 (44,279,522,me463) | PII-SVMP | * |
|  | 0.21 | 23▼ |  | 657.4 | 2 | YVELVIVADHR | Acont_SVMPI-2a_e44 | SVMP |  |
|  |  |  |  | 430.2 | 2 | QTDLLNR |  |  |  |
|  |  |  |  | 482.7 | 2 | GDIGIAYGAK |  |  |  |
|  |  |  |  | 626.8 | 2 | MVNTINEIYR | Acont_SVMPI-4a_e575 | SVMP |  |
|  |  |  |  | 634.8 | 2 | MoxVNTINEIYR |  |  |  |
|  |  |  |  | 657.4 | 2 | YVELVIVADHR | Acont_SVMPI-3a_e522 | SVMP |  |
|  |  |  |  | 534.3 | 2 | YNGDSDKIR |  |  |  |
|  | 0.03 | 9▼ |  | 406.2 | 3 | FSVGIVQDHSK | Acont_SVMPI-2a_e44 | SVMP |  |
|  |  |  |  | 521.8 | 2 | KDYQTFLK |  |  |  |
| **36** | 0.32 | 53▼ |  | 532.7 | 2 | NPLEECFR | Acont_LAAO-1_e495 | LAAO |  |
|  |  |  |  | 643.8 | 2 | SAGQLYEESFR |  |  |  |
|  |  |  |  | 562.3 | 2 | HDDIFGYEK |  |  |  |
|  |  |  |  | 557.8 | 2 | VIEIQQNDR |  |  |  |
|  |  |  |  | 430.2 | 3 | FWEEEGIHGGK |  |  |  |
|  |  |  |  | 524.6 | 3 | DPGLLEYPVKPSEK |  |  |  |
|  |  |  |  | 583.3 | 2 | IKFEPPLPPK |  |  |  |
|  |  |  |  | 441.7 | 2 | IFLTCTK |  |  |  |
|  |  |  |  | 652.8 | 2 | SFCYPSMIQR |  |  |  |
|  | 3.99 | 23▼ |  | 443.3 | 3 | YVELVIIADHR | Acont_SVMPI-4a_e575 | SVMP |  |
|  |  |  |  | 517.8 | 2 | IRQWIYR |  |  |  |
|  |  |  |  | 601.3 | 2 | APLAGMCDPNR |  |  |  |
|  |  |  |  | 529.3 | 2 | SVGTVQDHSK |  |  |  |
|  |  |  |  | 514.3 | 2 | KDYLTFLK |  |  |  |
|  |  |  |  | 492.7 | 2 | LFSDCSKK | Acont_SVMPI-2a_e(44,279,335,522...) | SVMP | * |
|  | 0.05 | 13▼ |  | 626.8 | 2 | MVNTINEIYR | Acont_SVMPI-4a_e575 | SVMP |  |
|  |  |  |  | 450.2 | 2 | DYLTFLK |  |  |  |
|  | 0.23 | 9▼ |  | 626.8 | 2 | MVNTINEIYR | Acont_SVMPI-4a_e575 | SVMP |  |
|  |  |  |  | 529.3 | 2 | SVGTVQDHSK |  |  |  |
|  |  |  |  | 514.3 | 2 | KDYLTFLK |  |  |  |
| **37** | 1.15 | 53▼ |  | 615.3 | 2 | LVIVADDVMVR | Acont_SVMPIII-4_98-1M61 | PIII-SVMP |  |
|  |  |  |  | 550.8 | 2 | YIHNLTALR |  |  |  |
|  |  |  |  | 587.3 | 2 | IFPCAPQNVK |  |  |  |
|  |  |  |  | 658.3 | 2 | (L)YNDNTYPCR |  |  |  |
|  | 0.29 | 23▼ |  | 626.8 | 2 | MVNTINEIYR | Acont_SVMPI-4a_e575 | SVMP |  |
|  |  |  |  | 634.8 | 2 | MoxVNTINEIYR |  |  |  |
|  |  |  |  | 450.2 | 2 | DYLTFLK |  |  |  |

Tables S1-S4 toxin key: CTL – C-type lectin; DISI – disintegrin; SVMP – snake venom metalloproteinase; LAO – L-amino acid oxidase; PLA_2_ – phospholipase A_2_; PLB – phospholipase B; SVMP – snake venom metalloproteinase; SVMPi – snake venom metalloproteinase inhibitor; SVSP – snake venom serine protease; VAP – vasoactive peptides (BPP/BIP).

**Table S5.** The distribution of unambiguously assigned proteins in the venom proteomes of 19F, 83M, 204F (parthenogen mother), and M74 (parthenogen; born 03/09/11). 19F and 83M were born 28/08/11 and 03/09/11, respectively, to unrelated females.

| **Peak** | **19F**  **unrelated female** | **83M**  **unrelated male** | **204F**  **mother** | **M74**  **parthenogen** | | | | **Unique gene expression in venom proteome** | |
| --- | --- | --- | --- | --- | --- | --- | --- | --- | --- |
|  |  |  |  |  | | | |  |  |
|  |  |  |  |  | | | | **19F/83M** | **M74** |
| **1** |  |  |  | Acont_BPP-1a_98 | | | |  |  |
| **2** | Acont_BPP-1a_98 | Acont_BPP-1a_98 | Acont_BPP-1a_98 | Acont_BPP-1a_98 | | | |  |  |
| **3** | Acont_BPP-1a_98 | Acont_BPP-1a_98 | Acont_BPP-1a_98 | Acont_BPP-1a_98 | | | |  |  |
| **4** | Acont_BPP-1a_98 | Acont_BPP-1a_98 | Acont_BPP-1a_98 | Acont_BPP-1a_98 | | | |  |  |
| **5** | Acont_BPP-1a_98 | Acont_BPP-1a_98 | Acont_BPP-1a_98 | Acont_BPP-1a_98 | | | |  |  |
| **6** | Acont_BPP-1a_98 | Acont_BPP-1a_98 | Acont_BPP-1a_98 | Acont_BPP-1a_98 | | | |  |  |
|  |  | Acont_BPP-1a_98 |  | Acont_BPP-1a_98 | | | |  |  |
| **7** | **Acont_SVMPII-3a_e261-2** |  |  |  | | | | **Long RGD disintegrin JAS04330** |  |
|  |  | Acont_DIS-2a_e351 |  | Acont_DIS-2a_e351 | | | |  | **Acostatin ab (13369,2 Da)** |
|  |  | Acont_SVMPII-5a_e416 |  |  | | | | **Dimeric RGD/RGD disintegrin e351/e866** |  |
| **8** | Acont_DIS-2a_e351 | Acont_DIS-2a_e351 |  | Acont_SVMPII-5a_e416 | | | |  |  |
|  | Acont_SVMPII-5a_e416 |  |  |  | | | |  |  |
|  | **Acont_SVMPII-1_me196** |  |  |  | | | | **Long RGD disintegrin JAS04331** |  |
| **9** | Acont_DIS-2a_e351 | Acont_DIS-2a_e351 | Acont_DIS-2a_e351 | Acont_DIS-2a_e351 | | | |  |  |
|  | **Acont_DIS-1_e866** |  |  |  | | | | **JAS04370 (RGD Disintegrin 3)** |  |
|  |  |  |  | Acont_SVMPII-5a_e416 | | | |  |  |
| **10** | Acont_DIS-2a_e351 | Acont_DIS-2a_e351 | Acont_DIS-2a_e351 | Acont_DIS-2a_e351 | | | |  |  |
|  | **Acont_DIS-1_e866** |  |  |  | | | | **JAS04370 RGD (Disintegrin 3)** |  |
|  |  |  | Acont_SVMPII-5a_e416 | Acont_SVMPII-5a_e416 | | | |  |  |
| **11** |  |  | Acont_DIS-2a_e351 | Acont_DIS-2a_e351 | | | |  |  |
|  |  |  |  | Acont_SVMPII-5a_e416 | | | |  |  |
| **12** |  |  | Acont_BPP-1a_98 |  | | | |  |  |
| **15** | Acont_PLA2-3a_e604 | Acont_PLA2-3a_e604 | Acont_PLA2-3a_e604 | Acont_PLA2-3a_e604 | | | |  |  |
| **16** | Acont_PLA2-3a_e604 | Acont_PLA2-3a_e604 | Acont_PLA2-3a_e604 | Acont_PLA2-3a_e604 | | | |  |  |
| **17** |  |  |  | Acont_PLA2-3a_e604 | | | |  |  |
| **18** | Acont_SVSP-5_e565 | Acont_SVSP-5_e565 | Acont_SVSP-5_e565 | Acont_SVSP-5_e565 | | | |  |  |
|  | Acont_PLA2-1a_e343 | Acont_PLA2-1a_e343 |  | Acont_PLA2-1a_e343 | | | |  |  |
|  |  |  |  | Acont_SVSP-3a_e242 | | | |  |  |
| **19** | Acont_PLA2-1a_e343 | Acont_PLA2-1a_e343 | Acont_PLA2-1a_e343 | Acont_PLA2-1a_e343 | | | |  |  |
|  | Acont_SVSP-12a_98-10M126 | Acont_SVSP-12a_98-10M126 | Acont_SVSP-12a_98-10M126 |  | | | |  |  |
|  | Acont_PLA2-4_e312 | Acont_PLA2-4_e312 |  |  | | | |  |  |
|  | Acont_SVSP-17a_e339 | Acont_SVSP-17a_e339 |  |  | | | |  |  |
|  | Acont_SVSP-14a_e824 | Acont_SVSP-14a_e824 | Acont_SVSP-14a_e824 |  | | | |  |  |
|  | Acont_SVSP-15a_e914 | Acont_SVSP-15a_e914 | Acont_SVSP-15a_e914 |  | | | |  |  |
|  |  | Acont_SVSP-5_e565 |  | Acont_SVSP-5_e565 | | | |  |  |
|  |  | Acont_Vespryn-1_e296 |  | Acont_Vespryn-1_e296 | | | |  |  |
|  |  |  | Acont_SVSP-7_e420 |  | | | |  |  |
|  |  |  | Acont_CTL-9a_e4 |  | | | |  |  |
|  |  |  | Acont_SVSP-2a_e957 |  | | | |  |  |
| **20** | Acont_PLA2-1a_e343 |  | Acont_PLA2-1a_e343 | Acont_PLA2-1a_e343 | | | |  |  |
|  | Acont_SVSP-12a_98-10M126 |  |  |  | | | |  |  |
|  | Acont_SVSP-14a_e824 |  | Acont_SVSP-14a_e824 |  | | | |  |  |
|  | Acont_SVSP-15a_e914 |  | Acont_SVSP-15a_e914 | Acont_SVSP-15a_e914 | | | |  |  |
|  | Acont_SVSP-2a_e957 |  | Acont_SVSP-2a_e957 |  | | | |  |  |
|  | Acont_CTL-9a_e4 |  |  |  | | | |  |  |
|  |  |  | Acont_SVSP-5_e565 | Acont_SVSP-11a_e565 | | | |  |  |
|  |  |  | Acont_SVSP-4_e9 |  | | | |  |  |
|  |  |  | Acont_PLA2-3a_e604 |  | | | |  |  |
|  |  |  |  | Acont_SVSP-11a_e515 | | | |  |  |
|  |  |  |  | Acont_SVSP-16a_e369 | | | |  |  |
| **20a** |  |  |  | Acont_SVSP-2a_e957 | | | |  |  |
|  |  |  |  | Acont_PLA2-1a_e343 | | | |  |  |
| **21** |  | Acont_SVSP-12a_98-10M126 |  |  | | | |  |  |
|  |  | Acont_SVSP-15a_e914 | Acont_SVSP-15a_e914 | Acont_SVSP-15a_e914 | | | |  |  |
|  |  | Acont_SVSP-14a_e824 | Acont_SVSP-14a_e824 | Acont_SVSP-14a_e824 | | | |  |  |
|  |  | Acont_SVSP-11a_e515 | Acont_SVSP-11a_e515 |  | | | |  |  |
|  |  |  | Acont_SVSP-16a_e369 |  | | | |  |  |
|  |  | Acont_SVSP-5_e565 |  |  | | | |  |  |
|  |  | Acont_CTL-9a_e4 |  |  | | | |  |  |
|  |  | Acont_PLA2-1a_e343 | Acont_PLA2-1a_e343 | Acont_PLA2-1a_e343 | | | |  |  |
|  |  |  | Acont_SVSP-18a_e982 | Acont_SVSP-18a_e982 | | | |  |  |
|  |  |  | Acont_SVSP-4_e9 |  | | | |  |  |
|  |  |  | Acont_PLA2-3a_e604 |  | | | |  |  |
|  |  |  |  | Acont_SVSP-2a_e957 | | | |  |  |
|  |  |  |  | Acont_PLA2-4_e312 | | | |  |  |
|  |  |  | Acont_SVSP-16a_e369 |  | | | |  |  |
| **22** | Acont_SVSP-16a_e369 |  | Acont_SVSP-16a_e369 | Acont_SVSP-16a_e369 | | | |  |  |
|  | Acont_SVSP-15a_e914 | Acont_SVSP-15a_e914 | Acont_SVSP-15a_e914 | Acont_SVSP-15a_e914 | | | |  |  |
|  | Acont_SVSP-14a_e824 | Acont_SVSP-14a_e824 |  | Acont_SVSP-14a_e824 | | | |  |  |
|  | Acont_SVSP-18a_e982 | Acont_SVSP-18a_e982 | Acont_SVSP-18a_e982 | Acont_SVSP-18a_e982 | | | |  |  |
|  | Acont_SVSP-17a_e339 |  |  |  | | | |  |  |
|  | Acont_SVSP-8_e14 |  | Acont_SVSP-8_e14 | Acont_SVSP-8_e14 | | | |  |  |
|  | Acont_SVSP-2a_e957 | Acont_SVSP-2a_e957 |  |  | | | |  |  |
|  |  | Acont_PLA2-4_e312 | Acont_PLA2-4_e312 | Acont_PLA2-4_e312 | | | |  |  |
|  |  | Acont_SVSP-11a_e515 |  | Acont_SVSP-11a_e515 | | | |  |  |
|  |  |  |  |  | | | |  |  |
|  |  |  | Acont_PLA2-1a_e343 |  | | | |  |  |
|  |  |  | Acont_SVMPI-3a_e522 |  | | | |  |  |
| **23** | Acont_SVSP-10_98-10M279 |  |  |  | | | |  |  |
|  | Acont_SVSP-6_98-10M191 |  | Acont_SVSP-6_98-10M191 | Acont_SVSP-6_98-10M191 | | | |  |  |
|  | Acont_PLA2-4_e312 |  |  |  | | | |  |  |
|  | Acont_SVSP-13a_e537 | Acont_SVSP-13a_e537 | Acont_SVSP-13a_e537 | Acont_SVSP-13a_e537 | | | |  |  |
|  |  | Acont_SVSP-16a_e369 | Acont_SVSP-16a_e369 | Acont_SVSP-16a_e369 | | | |  |  |
|  |  | Acont_SVSP-18a_e982 | Acont_SVSP-18a_e982 | Acont_SVSP-18a_e982 | | | |  |  |
|  | Acont_SVSP-8_e14 | Acont_SVSP-8_e14 | Acont_SVSP-8_e14 | Acont_SVSP-8_e14 | | | |  |  |
|  | Acont_SVSP-17a_e339 |  | Acont_SVSP-17a_e339 | Acont_SVSP-17a_e339 | | | |  |  |
|  | Acont_SVSP-7_e420 |  |  |  | | | |  |  |
|  |  |  | Acont_SVSP-3a_e242 | Acont_SVSP-3a_e242 | | | |  |  |
|  | Acont_SVSP-4_e9 | Acont_SVSP-4_e9 | Acont_SVSP-4_e9 | Acont_SVSP-4_e9 | | | |  |  |
|  |  |  | Acont_SVSP-1_e857 |  | | | |  |  |
|  |  |  | Acont_PLA2-3a_e604 |  | | | |  |  |
|  |  |  | Acont_PLA2-1a_e343 |  | | | |  |  |
|  |  |  |  |  | | | |  |  |
|  |  |  |  | Acont_PLA2-4_e312 | | | |  |  |
| **24** | Acont_SVSP-13a_e537 | Acont_SVSP-13a_e537 | Acont_SVSP-13a_e537 | Acont_SVSP-13a_e537 | | | |  |  |
|  | Acont_SVSP-6_98-10M191 | Acont_SVSP-6_98-10M191 | Acont_SVSP-6_98-10M191 |  | | | |  |  |
|  | Acont_SVSP-4_e9 | Acont_SVSP-4_e9 | Acont_SVSP-4_e9 | Acont_SVSP-4_e9 | | | |  |  |
|  | Acont_SVSP-8_e14 |  | Acont_SVSP-8_e14 |  | | | |  |  |
|  |  | Acont_SVSP-14a_e824 |  |  | | | |  |  |
|  |  | Acont_SVSP-3a_e242 | Acont_SVSP-3a_e242 |  | | | |  |  |
|  |  |  | Acont_SVSP-16a_e369 |  | | | |  |  |
|  |  |  | Acont_SVSP-17a_e339 |  | | | |  |  |
|  |  |  | Acont_LAAO-1_e495 |  | | | |  |  |
|  |  |  |  |  | | | |  |  |
| **25** | Acont_SVSP-13a_e537 | Acont_SVSP-13a_e537 |  |  | | | |  |  |
|  | Acont_SVSP-4_e9 | Acont_SVSP-4_e9 |  |  | | | |  |  |
|  | Acont_SVSP-17a_e339 | Acont_SVSP-17a_e339 |  |  | | | |  |  |
|  |  | Acont_SVSP-3a_e242 |  |  | | | |  |  |
|  |  | Acont_SVSP-14a_e824 |  |  | | | |  |  |
| **26** | Acont_SVSP-14a_e824 | Acont_SVSP-14a_e824 |  |  | | | |  |  |
|  | Acont_SVSP-13a_e537 |  |  |  | | | |  |  |
|  | Acont_SVSP-7_e420 | Acont_SVSP-7_e420 |  | Acont_SVSP-7_e420 | | | |  |  |
|  | **Acont_SVSP-19_vtb0097-2** | **Acont_SVSP-19_vtb0097-2** |  |  | | | | **SVSP-19** |  |
|  |  | Acont_SVSP-16a_e369 |  |  | | | |  |  |
|  |  |  |  | Acont_SVSP-6_98-10M191 | | | |  |  |
| **27** |  |  | Acont_LAAO-1_e495 |  | | | |  |  |
| **28** | Acont_LAAO-1_e495 | Acont_LAAO-1_e495 | Acont_LAAO-1_e495 |  | | | |  |  |
|  |  | Acont_SVSP-7_e420 |  |  | | | |  |  |
|  |  | Acont_SVSP-14a_e824 |  |  | | | |  |  |
| **29** | Acont_LAAO-1_e495 | Acont_LAAO-1_e495 | Acont_LAAO-1_e495 | Acont_LAAO-1_e495 | | | |  |  |
|  |  | Acont_SVMPIII-6a_e457 |  | Acont_SVMPIII-6a_e457 | | | |  |  |
|  | Acont_SVSP-2a_e957 |  |  |  | | | |  |  |
| **30** | Acont_LAAO-1_e495 |  |  |  | | | |  |  |
|  | Acont_SVMPIII-6a_e457 |  |  |  | | | |  |  |
| **31** | Acont_LAAO-1_e495 | Acont_LAAO-1_e495 |  | Acont_LAAO-1_e495 | | | |  |  |
|  | Acont_SVMPIII-6a_e457 | Acont_SVMPIII-6a_e457 |  | Acont_SVMPIII-6a_e457 | | | |  |  |
|  |  |  |  | Acont_SVMPII-1_me196 | | | |  |  |
| **32** | Acont_LAAO-1_e495 | Acont_LAAO-1_e495 | Acont_LAAO-1_e495 | Acont_LAAO-1_e495 | | | |  |  |
|  | Acont_SVMPIII-6a_e457 | Acont_SVMPIII-6a_e457 |  | Acont_SVMPIII-6a_e457 | | | |  |  |
|  | Acont_SVSP-3a_e242 |  |  |  | | | |  |  |
|  |  | Acont_SVMPIII-3_1f45 |  |  | | | |  |  |
|  |  | Acont_SVMPIII-2_me561 |  |  | | | |  |  |
|  |  | Acont_SVMPI-6a_e279 |  |  | | | |  |  |
|  |  | Acont_PLB-1_xng |  |  | | | |  |  |
| **33** | Acont_LAAO-1_e495 |  | Acont_LAAO-1_e495 | Acont_LAAO-1_e495 | | | |  |  |
|  | Acont_SVMPIII-3_1f45 |  |  | Acont_SVMPIII-3_1f45 | | | |  |  |
|  | Acont_SVMPIII-6a_e457 |  |  |  | | | |  |  |
|  |  |  |  | Acont_SVMPIII-2_me561 | | | |  |  |
|  |  |  |  | Acont_SVSP-7_e420 | | | |  |  |
|  |  |  |  | Acont_SVSP-10_98-10M279 | | | | |  |
|  |  |  |  | Acont_SVMPI-3a_e575 | | |  | |  |
|  |  |  |  | Acont_LAAO-1_e495 | | |  | |  |
| **34** | Acont_LAAO-1_e495 | Acont_LAAO-1_e495 |  |  | | |  | |  |
|  | Acont_SVMPIII-3_1f45 |  |  |  | | |  | |  |
|  | **Acont_PLB-1_xng** | **Acont_PLB-1_xng** |  |  | | | **Phospoholipase B** | |  |
|  | **Acont_SVMPI-6a_e279** | **Acont_SVMPI-6a_e279** |  |  | | | **JAS04336** | |  |
|  | Acont_SVMPII-5a_e416 | Acont_SVMPII-5a_e416 |  |  | | |  | |  |
|  | Acont_SVMPI-3a_e522 |  |  |  | | |  | |  |
|  |  | Acont_SVMPIII-2_me561 |  |  | | |  | |  |
| **35** | Acont_LAAO-1_e495 | Acont_LAAO-1_e495 | Acont_LAAO-1_e495 |  | | |  | |  |
|  | Acont_SVMPI-6a_e279 | Acont_SVMPI-6a_e279 |  |  | | |  | |  |
|  | Acont_SVMPI-2a_e44 | Acont_SVMPI-2a_e44 | Acont_SVMPI-2a_e44 |  | | |  | |  |
|  | Acont_SVMPI-3a_e522 | Acont_SVMPI-3a_e522 | Acont_SVMPI-3a_e522 | Acont_SVMPI-3a_e522 | | |  | |  |
|  | Acont_SVMPII-5a_e416 | Acont_SVMPII-5a_e416 |  | Acont_SVMPII-5a_e416 | | |  | |  |
|  | Acont_SVMPI-5a_e335 |  |  |  | | |  | |  |
|  | Acont_SVSP-5_e565 |  |  |  | | |  | |  |
|  |  | Acont_SVMPII-6a_e592 |  | Acont_SVMPII-6a_e592 | | |  | |  |
| **35a** |  | Acont_LAAO-1_e495 |  |  | | |  | |  |
|  |  | Acont_SVMPIII-7_98-5M50 |  |  | | |  | |  |
|  |  | Acont_SVMPI-2a_e44 |  |  | | |  | |  |
|  |  | Acont_SVMPII-2_me463 |  |  | | |  | |  |
|  |  | Acont_SVSP-18a_e982 |  |  | | |  | |  |
|  |  | Acont_SVSP-7_e420 |  |  | | |  | |  |
|  |  | Acont_SVSP-11a_e515 |  |  | | |  | |  |
|  |  | Acont_SVMPI-4a_e575 |  |  | | |  | |  |
|  |  | Acont_SVMPI-3a_e522 |  |  | | |  | |  |
| **36** | Acont_LAAO-1_e495 | Acont_LAAO-1_e495 | Acont_LAAO-1_e495 | Acont_LAAO-1_e495 | | |  | |  |
|  | Acont_SVMPII-2_me463 |  | Acont_SVMPII-2_me463 | Acont_SVMPII-2_me463 | | |  | |  |
|  | Acont_SVMPI-2a_e44 |  | Acont_SVMPI-2a_e44 | Acont_SVMPI-2a_e44 | | |  | |  |
|  | Acont_SVMPI-3a_e522 |  | Acont_SVMPI-3a_e522 | Acont_SVMPI-3a_e522 | | |  | |  |
|  | Acont_SVMPI-4a_e575 | Acont_SVMPI-4a_e575 |  | Acont_SVMPI-4a_e575 | | |  | |  |
|  |  |  |  |  | | |  | |  |
|  |  |  |  | Acont_SVMPIII-7_98-5M50 | | |  | |  |
| **37** | Acont_SVMPI-4a_e575 | Acont_SVMPI-4a_e575 | Acont_SVMPI-4a_e575 | Acont_SVMPI-4a_e575 |  | | | |  |
|  |  | Acont_SVMPIII-4_98-1M61 |  | Acont_SVMPIII-4_98-1M61 | | |  | |  |
|  |  |  | Acont_LAAO-1_e495 | Acont_LAAO-1_e495 | |  | | |  |
|  |  |  | Acont_SVSP-6_98-10M191 |  | |  | | |  |
|  |  |  | Acont_SVMPI-3a_e522 |  | |  | | |  |
|  |  |  |  | Acont_SVMPI-2a_e44 | |  | | |  |
|  |  |  |  |  | |  | | |  |
